# Optimizing LaNiO_3_ Perovskite as Catalyst Precursor for the Revalorization of Biogas by Dry Reforming of Methane

**DOI:** 10.3390/molecules31183284

**Published:** 2026-09-16

**Authors:** Álvaro Díaz-Verde, Jonathan Cavazzani, Antonella Glisenti, María José Illán-Gómez

**Affiliations:** 1 Carbon Materials and Environment Research Group, Inorganic Chemistry Department and Materials Institute of the University of Alicante (IUMA), Faculty of Sciences, University of Alicante, Ctra. San Vicente del Raspeig s/n, San Vicente del Raspeig, 03690 Alicante, Spain; alvaro.diaz@ua.es; 2 Innovative Materials and Processes for Advanced Environmental Clean Technologies Research Group, Department of Chemical Sciences, University of Padova, Via F. Marzolo, 1, 35131 Padova, Italy; jonathan.cavazzani@phd.unipd.it (J.C.); antonella.glisenti@unipd.it (A.G.); 3 National Council Research Institute of Condensed Matter Chemistry and Technology for Energy (CNR ICMATE), Via F. Marzolo, 1, 35131 Padova, Italy

**Keywords:** perovskite, nickel, cobalt, methane dry reforming, biogas, syngas

## Abstract

The Dry Reforming of Methane (DRM) is an efficient route to produce syngas (which is the feedstock for the production of synthetic fuels via the Fischer–Tropsch process) or hydrogen from methane and carbon dioxide. This work evaluates several nickel-based perovskite-type mixed oxides (La_x_NiO_3_, La_0.8_Ni_0.9_M_0.1_O_3_ (*M* = Co, Fe and Mn) and La_0.8_Ni_1−y_Co_y_O_3_) as precursors of the active phase (Ni) for the DRM reaction. Although La_0.8_NiO_3_ yields slightly smaller Ni particles after reduction, it promotes the accumulation of a significant amount of carbonaceous material during DRM. Partial Ni substitution with Co, Fe and Mn improves redox stability, with the La_0.8_Ni_0.75_Co_0.25_O_3_ formulation being the most effective for removing the carbonaceous deposits under a 25% CH_4_, 25% CO_2_ (50% He) reactant atmosphere. However, when this formulation is used as a catalyst precursor for DRM under more realistic conditions (i.e., employing a feed that simulates real biogas), CH_4_ conversion and H_2_ yield decrease, and the carbon accumulated increases due to the greater influence of the parallel reactions. As a positive sign for future applications or research, under these conditions, the addition of 0.5% O_2_ to the feed and the use of a CO_2_ regeneration step decreased the amount of carbonaceous material deposited.

## 1. Introduction

Climate change is currently one of the most discussed and complex topics due to its harmful effects on biological systems and human health. Greenhouse gases (GHGs), such as carbon dioxide and methane [1,2], are considered the main cause of this problem. Since the Industrial Revolution, human activities have provoked a continuous increase in the emissions of GHGs into the atmosphere and, to control these emissions, increasingly strict limits have been established [3]. In this scenario, the optimization of industrial processes that promote the use of GHGs for the synthesis of high value-added products is urgently needed to promote a circular economy. Thus, the Dry Reforming of Methane (DRM, Reaction (1)) offers an efficient route for using methane and carbon dioxide [4,5], which can be obtained, among other sources, from biogas, which is a gaseous mixture generated by anaerobic digestion of waste generated by multiple economic sectors [6]. The DRM reaction allows the production of synthesis gas, which is a mixture of hydrogen and carbon monoxide [7,8] used as a raw material for the production of synthetic fuels via the Fischer–Tropsch process [9], or for hydrogen production. However, as the DRM reaction is highly endothermic and kinetically slow, it must be carried out at high operating temperatures and requires the use of catalysts [4,5]. Furthermore, the DRM reaction competes with other (parallel) reactions, such as methane decomposition, the Reverse Water–Gas Shift (RWGS) and the Boudouard reaction (Reactions (2)–(4)), which decrease the selectivity toward the required products (syngas or H_2_) and deactivate the catalyst [10,11]. Therefore, the search for highly efficient and low-cost catalytic formulations is a challenging and highly relevant task.

(1) $$CH_{4}+CO_{2}\to 2\;CO+2\;H_{2}$$

(2) $$CH_{4}\to C+2\;H_{2}$$

(3) $$CO_{2}+H_{2}\to CO+H_{2}O$$

(4) $$2\;CO\to C+CO_{2}$$

Noble metal-based catalytic formulations, in particular those based on Pt, Rh and/or Ru, are the most active for the DRM reaction [12]. However, they are highly expensive (due to their scarcity), and the search for more economical alternatives is a key challenge. In this line, Ni- and Co-based catalysts are proposed as alternatives, but they undergo a significant deactivation due to the sintering of the metallic active phases and the formation of carbonaceous deposits [13]. Thus, in an attempt to obtain solids with optimized catalytic properties, thermally stable mixed oxides containing these two metals have been widely explored [14,15,16]. Specifically, the formation of carbonaceous deposits can be minimized with Ni-based catalysts through the formation of Ni particles with low average sizes (less than 10 nm) and strong metal-support interactions [17]. Additionally, to minimize catalyst deactivation due to the accumulation of carbonaceous materials, other routes have been explored, such as: (i) the Oxidative Dry Reforming of Methane (ODRM) reaction, which requires the presence of small amounts of O_2_ in the reactant atmosphere (CH_4_ + CO_2_) [18] and (ii) the use of a regeneration step with pure CO_2_ to promote the reverse Boudouard reaction [19].

In this context, mixed oxides with perovskite structure (ABO_3_), composed by non-noble metals with suitable ionic radii for occupying the A and B sites (with 12 and 6 coordination, respectively) [20,21], exhibit interesting catalytic properties that make them suitable in a wide variety of reactions, including the DRM reaction. Thus, perovskite-type materials are proposed as precursors of the active phases because, after a pre-reduction treatment, a B/A_x_O_y_ system is formed by the reduction in B cation, which is selectively ex-solved as metallic (active) particles [22,23]. In this regard, LaNiO_3_ perovskite formulation has been extensively used as a precursor of the Ni/La_2_O_3_ catalytic system with improved properties in comparison to catalysts prepared by impregnation of the La_2_O_3_ support with a Ni precursor [24]. To optimize this formulation, different strategies have been proposed, with partial substitution of the metal at the B site being one of the most useful, as it directly modifies the characteristics of the metallic active phase, affecting both CH_4_ activation and the formation of carbonaceous material. In this line, the incorporation of transition metals like Co, Fe and Mn as dopants is expected to enhance the dispersion of the Ni metallic active sites and the CO_2_ adsorption, while minimizing the formation of carbonaceous deposits [25,26,27,28]. Additionally, the use of an under-stoichiometric proportion of A or B cations could affect the catalytic activity; however, to the best of the author’s knowledge, this variable has not been analyzed in detail for the DRM reaction.

Considering this background, in this work, three series of LaNiO_3_ perovskites—La_x_NiO_3_ (*x* = 1, 0.9, 0.8 and 0.7), La_x_Ni_1−y_M_y_O_3_ (*x* = 0.8, *y* = 0.1, *M* = Co, Fe and Mn) and La_x_Ni_1−y_Co_y_O_3_ (*x* = 0.8, *y* = 0.1, 0.25 and 0.5)—were synthesized, characterized and tested as precursors of the active phase (Ni) in the DRM reaction under highly concentrated reactant atmospheres with CH_4_/CO_2_ ratios of 1 and 1.67, with the latter simulating the use of real biogas. Finally, the best formulation was selected for DRM catalytic tests under more realistic reaction conditions, including: (i) the presence of small percentages of O_2_ in the CH_4_ + CO_2_ atmosphere (ODRM reaction) to try to minimize the formation of carbonaceous deposits [18] and (ii) the use of a regeneration step with pure CO_2_ to remove the deposited carbonaceous material.

## 2. Results and Discussion

### 2.1. Optimization of LaNiO_3_ Formulation by Decreasing the La Content (LxN Samples)

The series of samples under study in this section will be denoted as LxN, with “L” and “N” representing La and Ni elements and “x” indicating the La stoichiometry (except for LaNiO_3_, in which “x” is omitted).

#### 2.1.1. Characterization of the Fresh LxN Samples

**Figure 1:**
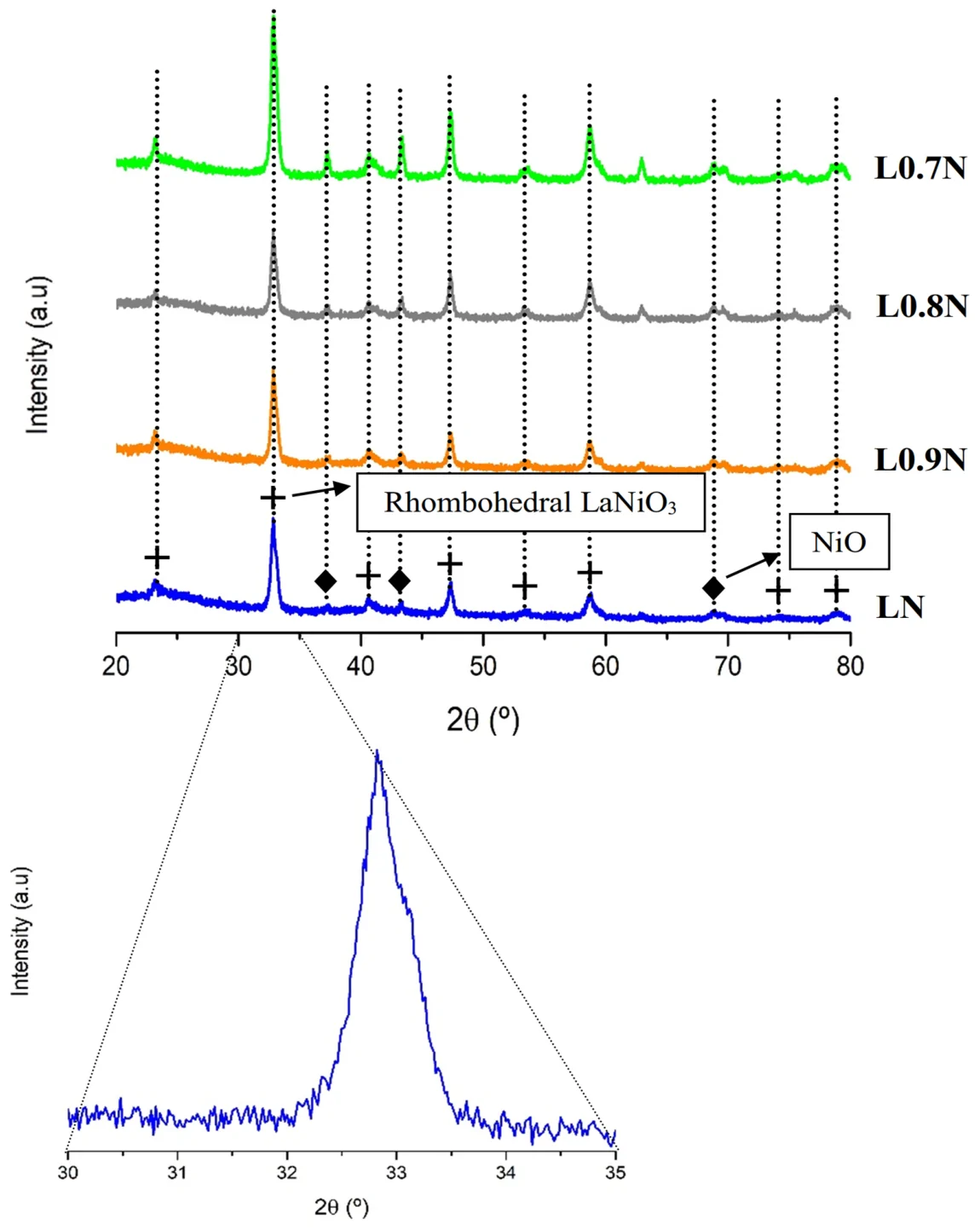
XRD patterns of the fresh LxN samples.

The X-Ray Diffraction (XRD) patterns of the LxN samples are shown in Figure 1. All samples exhibit the perovskite structure as the main crystalline phase; however, as the tolerance factors (*t*, included in Table 1) are lower than 1, the rhombohedral symmetry (0.96 ≤ *t* < 1) [29,30] is expected to be achieved, as revealed by the XRD peaks (JCPDS-ICDD 79-2451). Note that the *t* value diminishes with decreasing La percentage (Table 1), suggesting that the presence of cationic vacancies causes an orthorhombic distortion of the lattice (*t* < 0.96) [29,30]. In fact, in the magnification of the 2θ range between 30° and 35° shown in Figure 1, asymmetry in the main diffraction peak in the LaNiO_3_ phase, as expected for the R$\bar{3}$c rhombohedral symmetry, is observed [31,32,33]. On the other hand, the NiO face-centered cubic structure (JCPDS-ICDD 78-0643) is detected only as a minor crystalline phase in all the samples, and its formation is probably related to heterogeneous interactions among the metallic precursors during the formation of the LaNiO_3_ structure [34], specifically the Ni species with the La precursor. In fact, the intensity of the NiO signals seems to increase as the La content diminishes, as also reported by S. Singh et al., who observed the inclusion of NiO in local regions of the LaNiO_3_ perovskite with La impoverishment [35].

**Figure 2:**
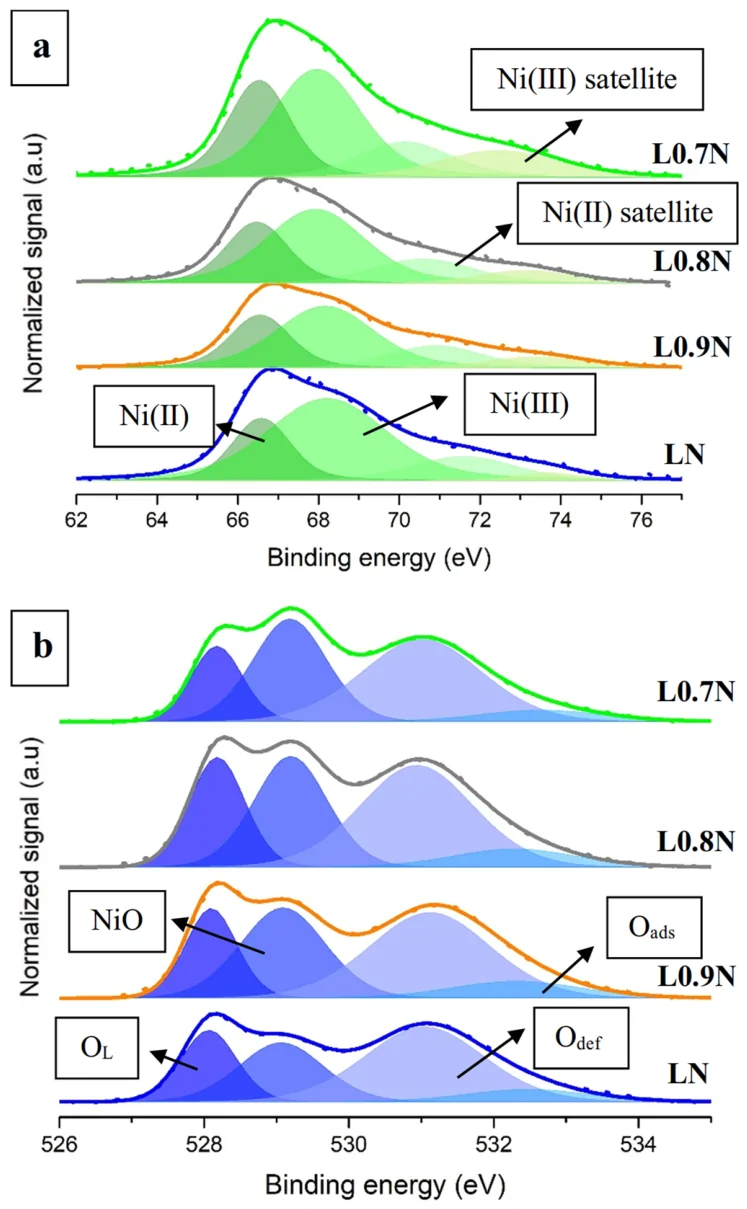
XPS spectra of the Ni 3p (**a**) and O 1s (**b**) transitions for the fresh LxN samples.

Regarding the chemical surface composition, Figure 2 and Table 1 display the main XPS (X-ray Photoelectron Spectroscopy) data for the Ni 3p and O 1s transitions.

To determine the Ni species present on the surface, the Ni 2p transition is commonly analyzed [36,37,38] but, for the LxN formulations, the Ni 2p transition overlaps with the La 3d signal and, consequently, the Ni 3p transition (Figure 2a) was employed instead [24,39]. According to the literature [40,41,42], the Ni 3p signal can be deconvoluted into the following contributions: (i) Ni(II) at approximately 66 eV, (ii) Ni(III) at around 68 eV, and (iii) Ni(II) and Ni(III) satellites at approximately 70 and 72 eV, respectively. The XPS data suggest that, due to the La deficiency, the amount of Ni(II) on the surface (see Ni(III)/Ni(II) ratio values) increases, which is the opposite of the expected trend of counteracting the positive charge deficiency due to the under-stoichiometric amount of La. However, this finding aligns with the bulk XRD results, which reveal the existence of a higher proportion of NiO as the amount of La decreases. Additionally, the Ni/(La + Ni) XPS ratios (calculated using the areas of the deconvoluted signals) provide information about the distribution of the Ni species on the surface, because an experimental (XPS) ratio higher than the nominal one (determined based on the perovskite formulation) indicates an accumulation of Ni species on the surface, whereas the opposite suggests a depletion of these species. Note that, even though the Ni/(La + Ni) XPS ratios do not follow a clear trend with the La content, they reveal that L0.7N presents the highest surface Ni enrichment.

The O 1s signal (Figure 2b) shows the four contributions usually found for perovskite-type mixed oxides [43,44]: (i) at 528 eV, the oxide anions present in the LaNiO_3_ lattice (O_L_), (ii) at 529 eV, the oxygen from the NiO phase, (iii) at ca. 531 eV, oxygen with low coordination that corresponds to the oxygen vacancies formed on the surface (O_def_), and (iv) at 533 eV, adsorbed oxygen, carbonates and hydroxyl groups (O_ads_). To indirectly estimate the amount of oxygen vacancies on the surface, the O_L_/(La + Ni) XPS ratio is usually calculated [45], and in the current context, O_L_ contribution only considers lattice oxygen from the perovskite phase, thus excluding that corresponding to NiO. As the XPS ratios (obtained from the area of the corresponding deconvolutions) are lower than the theoretical ones (calculated from the samples composition), the presence of oxygen vacancies on the surface is confirmed and, since the difference between the XPS ratio and the theoretical value increases as the percentage of La decreases, it seems that the formation of a higher amount of oxygen vacancies is promoted. So, this compensation mechanism seems to contribute to counteracting the decrease in the positive charge due to the progressive lowering in the La content.

**Figure 3:**
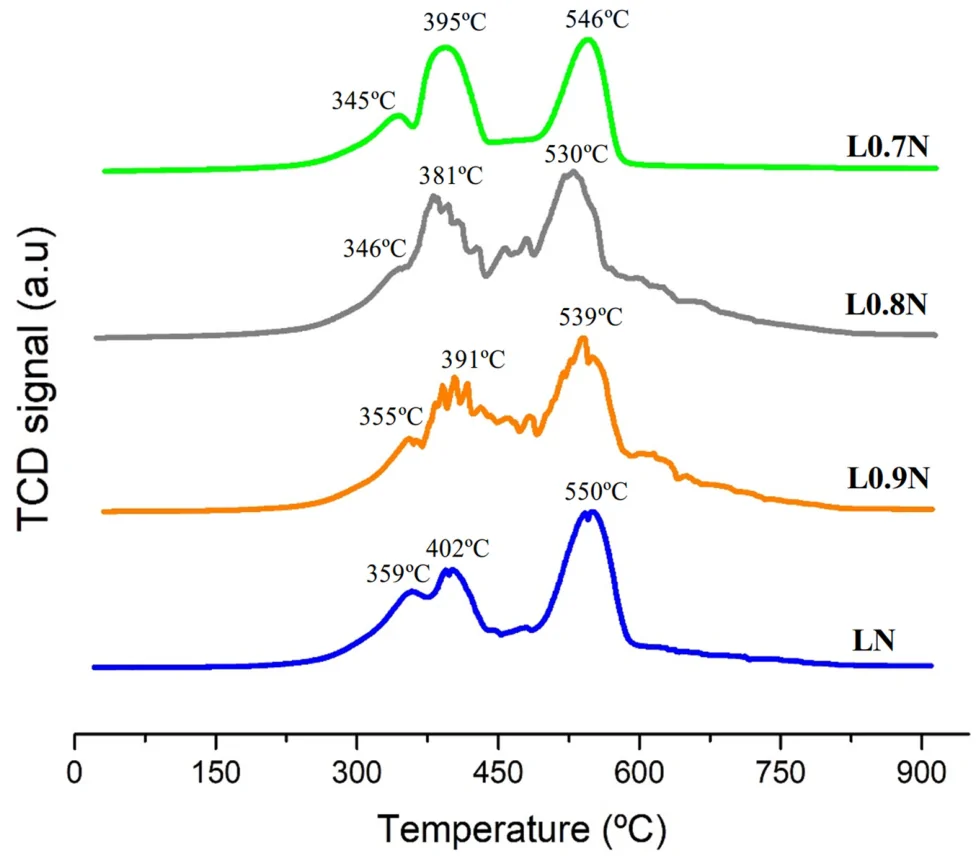
H_2_-TPR profiles of the fresh LxN samples.

On the other hand, to estimate the reducibility and the redox properties of the LxN samples, Temperature-Programmed Reduction with H_2_ (H_2_-TPR) tests were developed, being the H_2_ consumption profiles shown in Figure 3. The different reduction steps undergone by the Ni species can be distinguished by the temperature of their reduction peak maxima [46]: (i) the peak located between 200 °C and 500 °C corresponds to the reduction of the LaNiO_3_ perovskite to La_2_Ni_2_O_5_ (also formulated as LaNiO_2.5_); and (ii) the peak appearing between 500 °C and 700 °C is due to the reduction of La_2_Ni_2_O_5_ to Ni/La_2_O_3_. Note that, in all the H_2_-TPR profiles, a shoulder at low temperatures is additionally detected, which seems to correspond to the reduction in the NiO species (previously identified by XRD) with weak interactions with the perovskite phase [47]. Indeed, the temperatures at which this low-intensity signal appears are consistent with that for the reduction in bulk NiO [48,49]. As observed in Figure 3, the temperature at which the maximum occurs shifts to lower values for the La-deficient samples, suggesting that the presence of a higher amount of oxygen vacancies facilitates the reduction processes [50]. Considering the amount of H_2_ consumed (included in Table 2), it is evident that these values also follow the trend observed for the O_L_/(La + Ni) XPS ratio. Finally, note that this effect is more pronounced for the L0.8N sample, not only because it presents the highest population of oxygen vacancies on the surface, but also as it features the highest population of reducible NiO species, according to the V_NiO_ values presented in Table 2.

#### 2.1.2. Characterization of the LxN Reduced Samples

As described in Section 3, the samples were subjected to a pre-reduction step (under a 5% H_2_/He atmosphere at 500 °C for 2 h) prior to the catalytic tests to obtain the active phase for the DRM reaction. The reduction temperature (500 °C) was selected based on the results shown in Figures S2 and S3 and Table S1, included in the Supplementary Information file.

To determine the state of the samples at the beginning of the DRM experiments, the reduced samples were characterized by XRD, XPS, FE-SEM (Field Emission Scanning Electron Microscopy) and TEM (Transmission Electron Microscopy), with the main results being shown below.

**Figure 4:**
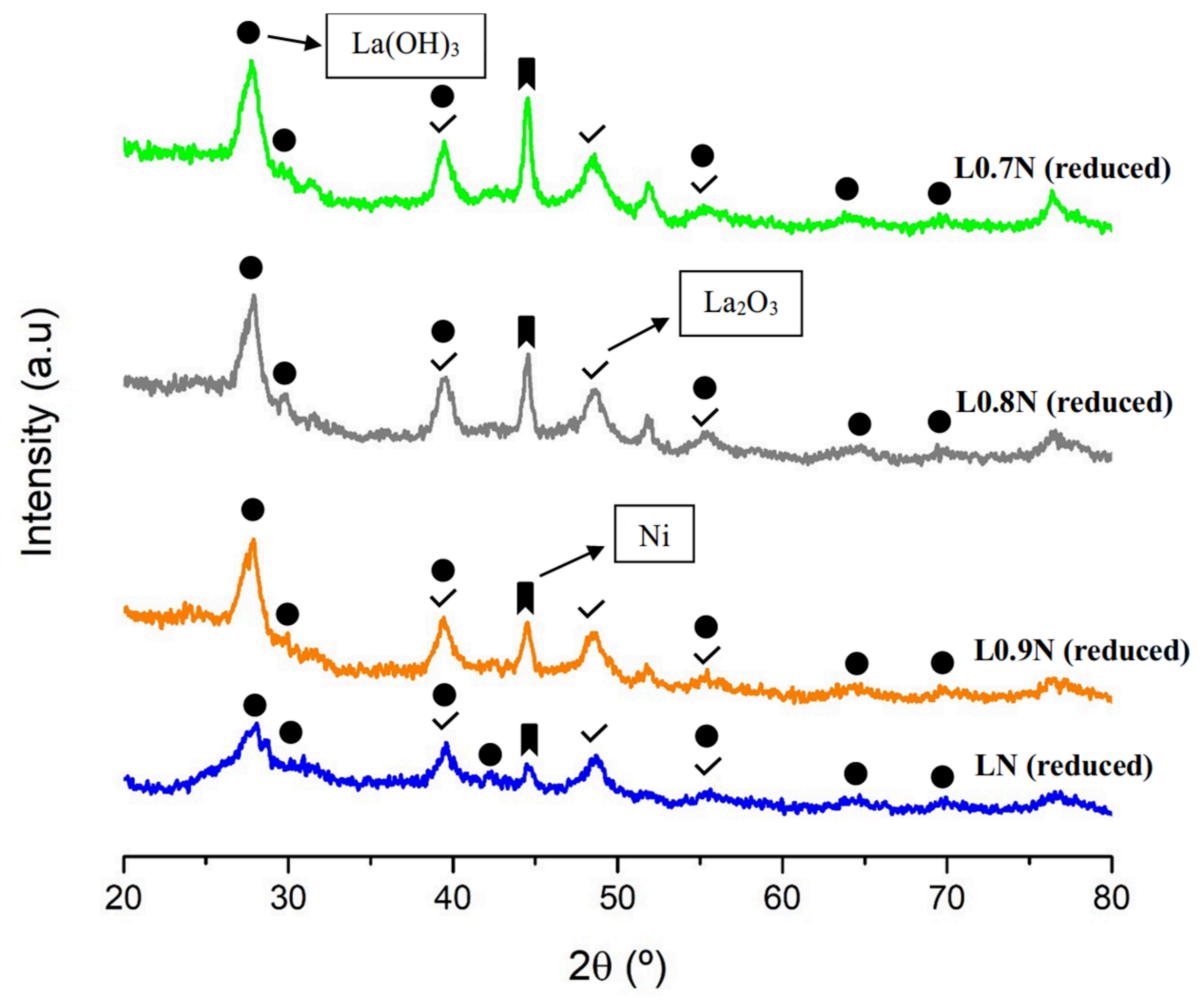
XRD patterns of the LxN samples reduced at 500 °C.

In the XRD diffractograms of the reduced samples (see Figure 4), the following crystalline phases were detected: (i) La_2_O_3_ (JCPDS-ICDD 74-1144), (ii) Ni (JCPDS-ICDD 04-0850) and (iii) La(OH)_3_ species (JCPDS-ICDD 36-1481). Thus, the presence of the Ni/La_2_O_3_ system is confirmed for all the samples.

**Figure 5:**
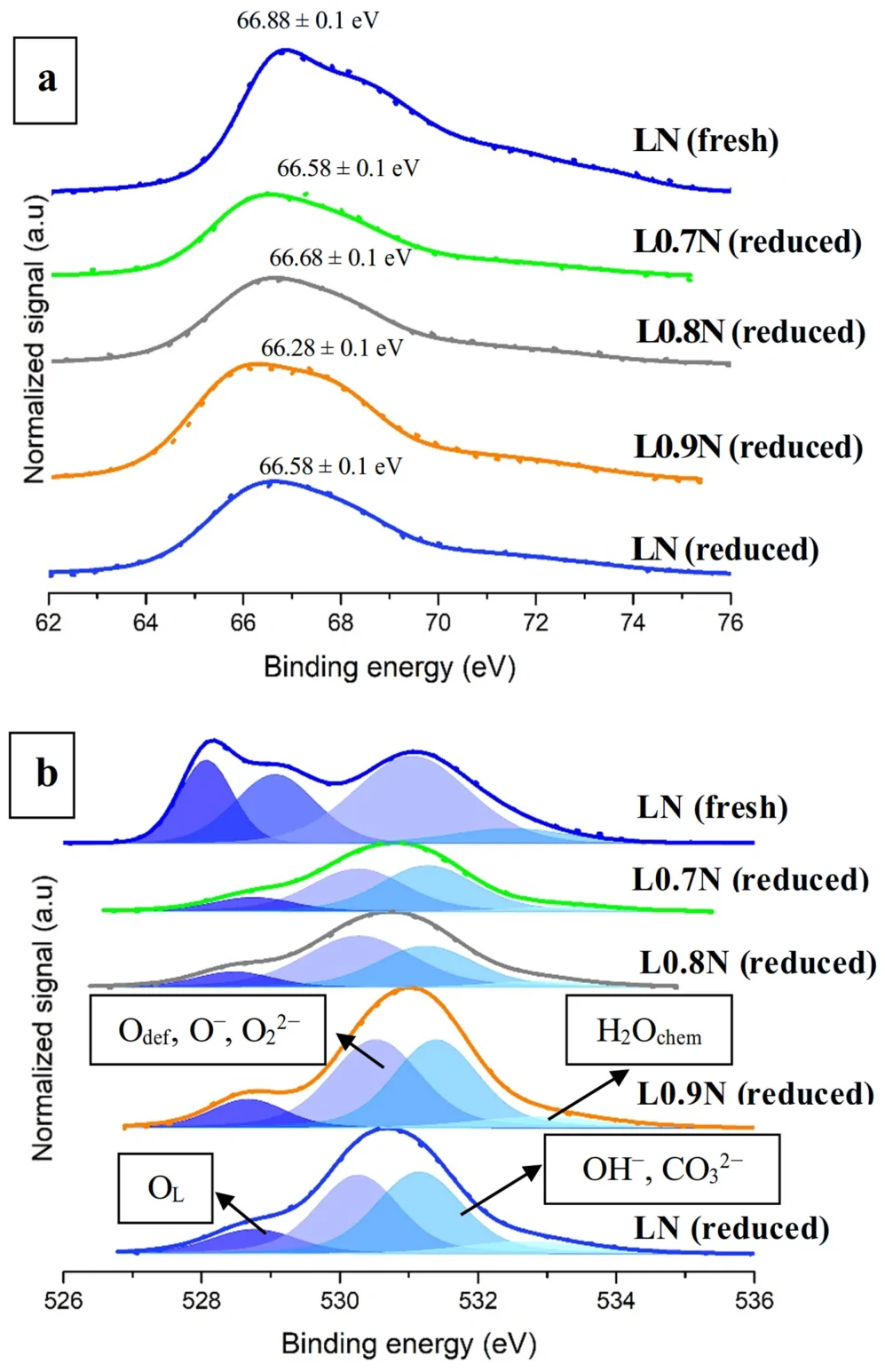
XPS spectra of the Ni 3p (**a**) and O 1s (**b**) transitions for the reduced LxN samples, together with the fresh LN formulation (included as a reference).

The XPS analysis of the Ni 3p transition spectra of the reduced samples (Figure 5a) reveals a chemical shift towards lower binding energies in comparison to the fresh LN sample. In the literature, the presence of Ni(0) is proposed if the Ni 3p signal appears at approximately 66.6 eV [51]. Note that the maximum of the Ni 3p spectra of the reduced samples is close to this value, suggesting that reduced Ni should be present on the surface. However, as the Ni 3p signal is extended towards higher binding energies, some contribution of Ni(II) and Ni(III) species is suggested. Consequently, the LxN samples could not be completely reduced before the DRM tests, and it is possible that a fraction of reduced Ni would be oxidized due to air exposition prior the XPS analysis [52]. Finally, the assignment of the O 1s deconvolutions is slightly different in the reduced samples compared to the fresh ones, as the lattice oxygen corresponds to the oxygen of the La_2_O_3_ support. Thus, the different contributions correspond to [51,53,54,55]: (i) oxide ions of the La_2_O_3_ support, which appear at approximately 529 eV (O_L_), (ii) low-coordination oxygen species present on the surface of the remaining LaNiO_3_ at around 530 eV (O_def_), (iii) hydroxyl and carbonate groups between 531 eV and 532 eV, and iv) chemisorbed water (which was generated during the reducing treatment) and peroxide groups at approximately 533 eV. Note that the contribution from the lattice oxygen of the NiO phase was not clearly detected, which may also suggest the presence of Ni(0) species on the surface.

**Figure 6:**
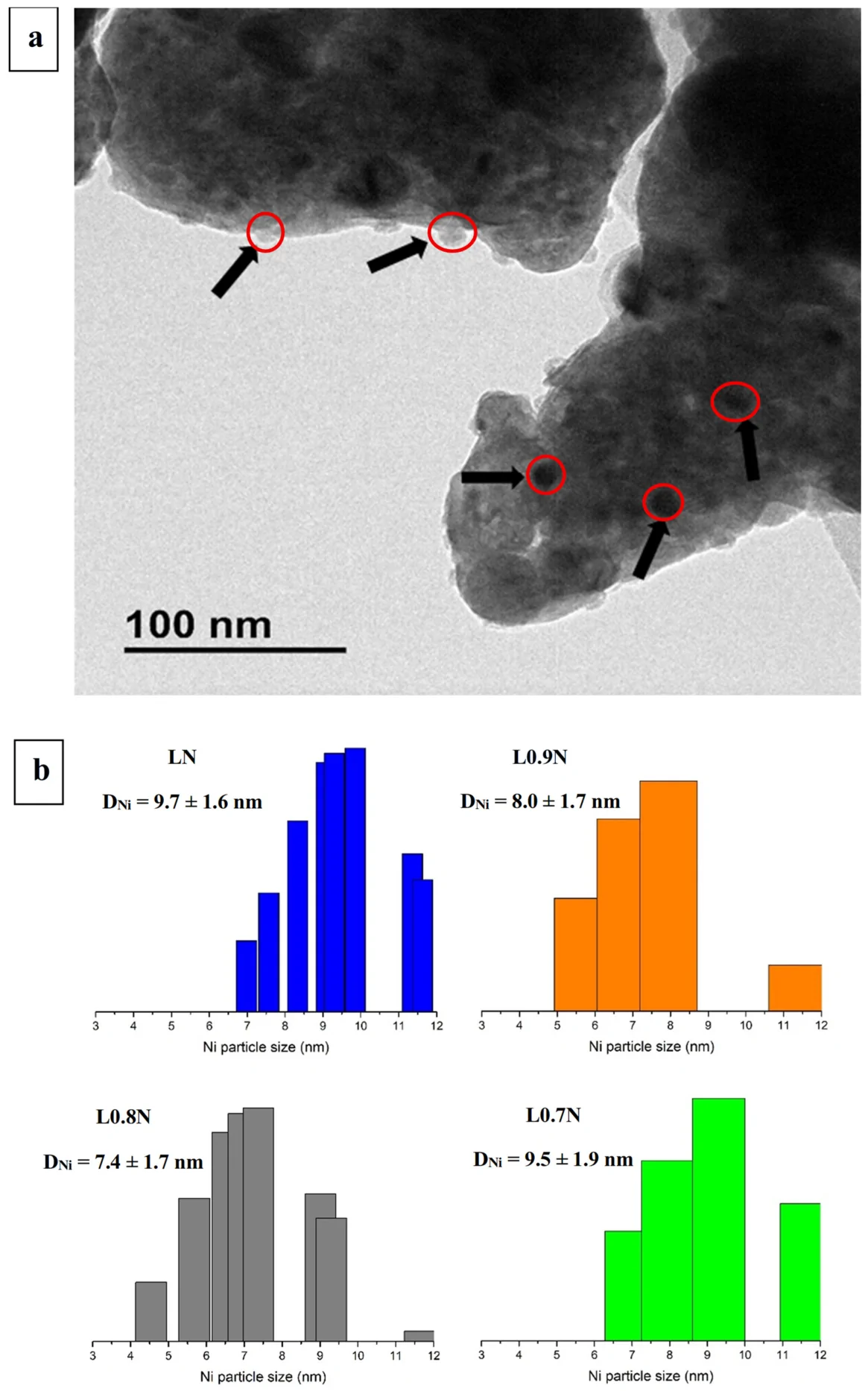
TEM image of the reduced LN formulation (**a**) and Ni particle size distribution of the reduced LxN samples, including the average Ni particle sizes (D_Ni_) (**b**).

The FE-SEM images of the LN reduced sample (Figure S4 in the Supplementary Information file) show the presence of irregular-shaped agglomerates [56]. The Ni average particle size and the Ni particle size distribution of the reduced samples were estimated by TEM. As an example, Figure 6 shows the TEM image of the reduced LN sample, in which the Ni particles (red circles) on the surface are distinguished by their darker color and circular shape, whereas those located at the boundaries of the La_2_O_3_ phase are distinguished by their higher transparency. According to the Ni particle size distribution, the L0.8N sample presents a slightly lower Ni average particle size (D_Ni_) than the other samples in the series.

#### 2.1.3. Catalytic Activity for DRM Reaction and Characterization of the LxN Used Samples

**Figure 7:**
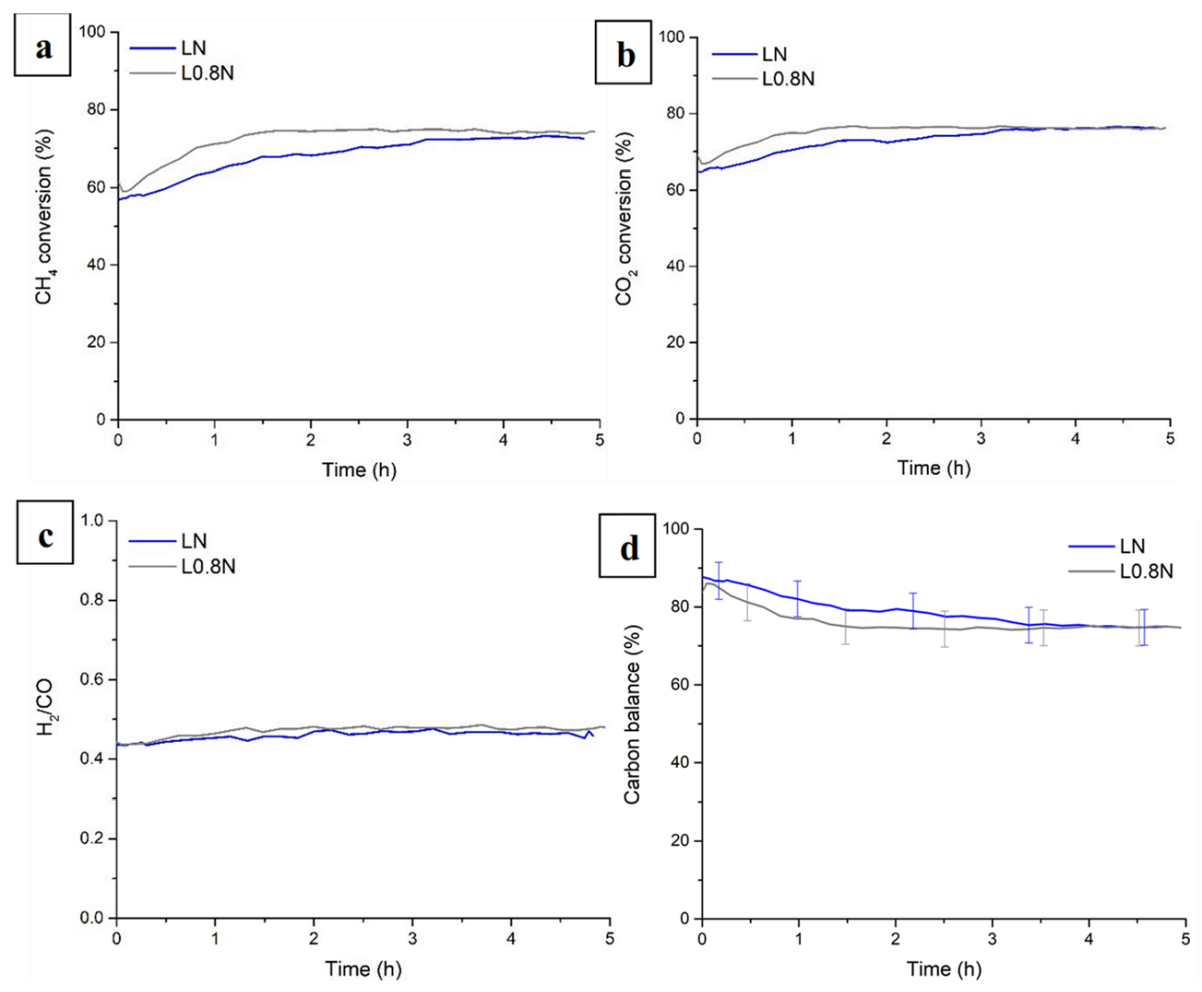
CH_4_ (**a**) and CO_2_ (**b**) conversions, H_2_/CO ratio (**c**) and carbon balance (**d**) during the DRM reaction at 700 °C (25:25 CH_4_:CO_2_) for the LN and L0.8N samples.

The catalytic tests for the DRM reaction were performed under 25% CH_4_ and 25% CO_2_ (denoted as 25:25, and using 50% He as a balance gas) reactant atmosphere at 700 °C using two representative samples: LN as reference, and L0.8N as it presents a slightly lower average Ni particle size and a higher reducibility than the other LxN samples. The most significant activity data obtained for both formulations are displayed in Figure 7 and Table 3. Additionally, Figure S5 and Table S3 present the catalytic results for all the samples of the LxN series.

First of all, the CH_4_ and CO_2_ conversions after 5 h of reaction are between 70 and 77%, being close to the thermodynamic values at 700 °C (83% for both reactants) [57] and in the range of the data published in the literature. In fact, using a LaAl_0.2_Ni_0.8_O_3_ perovskite at 850 °C, and under a 10:10 (CH_4_:CO_2_) atmosphere, the registered conversions are similar to those shown for the LN and L0.8N formulations [58], which are also close to the conversions at 700 °C (CH_4_/CO_2_ = 1) for Rh- and Ru-supported formulations (being superior than those registered for a Pt/Al_2_O_3_ catalyst) [59]. Moreover, the catalytic performance of these two formulations improves compared to that reported for a Ni/La_2_O_3_ catalyst (synthesized by direct impregnation of Ni over La_2_O_3_) under similar reaction conditions (15:15 (CH_4_:CO_2_) at 650 °C) [60]. Furthermore, the positive values of ΔΧ_CH4_ and ΔΧ_CO2_ parameters (included in Table S5 and calculated as the difference between the reactant conversions at 5 h and at 30 min of reaction) reveal an increase in both conversions during time on stream, which is likely attributable to a further reduction in the nickel species during the DRM test promoted by the reducing nature of CH_4_ [61]. Thus, this observation seems to confirm that LxN are partially reduced before the DRM tests, as the reduction treatment took place in situ and, consequently, reoxidation due to air exposition did not occur. In fact, L0.8N shows the lowest ΔΧ_CH4_ and ΔΧ_CO2_ values due to its higher reducibility (see H_2_-TPR results), suggesting a higher degree of reduction at the beginning of the reaction. Regarding the H_2_ yields, the registered values are close to those published for other perovskite-based formulations (such as LaNi_0.75_Fe_0.25_O_3_/SiO_2_ [62] or La_0.8_Sr_0.2_Ni_0.8_Zr_0.2_O_3_ [63]) and for Ni/La_2_O_3_ catalysts [60] tested under similar conditions, even though the values are far from the thermodynamic values (82%) [64]. This difference suggests that a high fraction of hydrogen is consumed through the RWGS reaction, which also justifies the low H_2_/CO ratios, which differ significantly from those reported for Ni/La_2_O_3_ and for Pt-, Ru- and Rh-supported formulations (that are equal to or higher than unity) [59,60]. Finally, note that the amount of carbonaceous material deposited during the reaction increases for the La-deficient sample and, as expected, is significantly higher than those values reported for Pt- and Ru-supported formulations [65], and also higher than those registered for Ni/La_2_O_3_ catalysts [66] due to the improved performance of the exsoluted Ni particles from the perovskite structure. Furthermore, according to the carbon balance profiles featured in Figure 7d, this carbonaceous material seems to accumulate in slightly greater amounts on the L0.8N sample than on LN during the first 4 h on stream.

The TEM characterization of the used samples (see Figure S6 in the Supplementary Information file) reveals the presence of carbon nanofibers where only several Ni particles were encapsulated, thus ensuring stable catalytic behavior. Additionally, as revealed by the Ni average particle sizes included in Table 4, the sintering of the Ni particles takes place during the DRM reaction, since the used samples present higher values than the reduced ones. Note that the increase in the average Ni particle size is more pronounced for L0.8N, which likely justifies the larger accumulation of carbonaceous deposits found after the reaction [67].

Thus, even though a larger amount of carbonaceous material is deposited on L0.8N than on LN, this sample was selected for optimization, as its features a slightly lower average Ni particle size and higher reducibility than the LN formulation.

### 2.2. Effect of the Partial Substitution of Ni by Co, Fe and Mn in the La_0.8_NiO_3_ Formulation (L0.8NM0.1 Series)

In this section, the substituted samples will be denoted as L0.8NM0.1, representing “M” the dopant (Co, Fe or Mn), and “0.1” the degree of substitution of Ni by each metal.

#### 2.2.1. Characterization of the L0.8NM0.1 Fresh Samples

Table 5 displays the surface weight percentages of Co, Fe and Mn (determined by X-Ray Microfluorescence (μ-XRF)) and the bulk weight percentages (obtained by Inductively Coupled Plasma Optical Emission Spectroscopy (ICP-OES)) for the L0.8NM0.1 series of samples. For Co and Fe, both techniques reveal similar contents that are close to the theoretical percentage. However, the surface Mn percentage is significantly lower than the bulk content, suggesting that Mn is located in inner positions of the perovskite lattice than Co or Fe.

**Figure 8:**
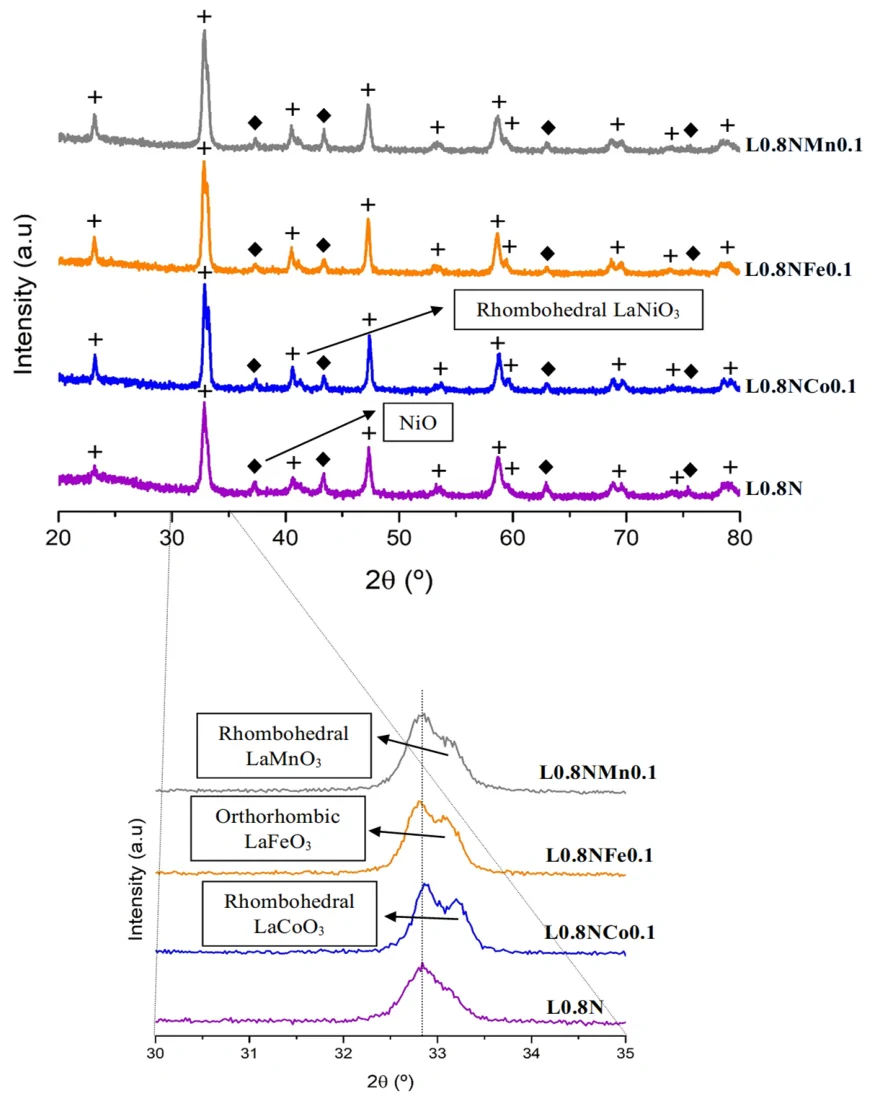
XRD patterns of the fresh L0.8N and L0.8NM0.1 samples.

Regarding the structural characterization, Figure 8 displays the XRD profiles of the L0.8NM0.1 series of samples, including that corresponding to the L0.8N perovskite for comparison. Like L0.8N sample, rhombohedral LaNiO_3_ perovskite (in which Ni is partially replaced by Mn, Fe or Co) and NiO crystalline phases have been identified. However, the splitting of the XRD signal located at approximately 33° suggests the presence of perovskite-type phases corresponding to LaCoO_3_ (JCPDS-ICDD 48-0123), LaFeO_3_ (JCPDS-ICDD 75-0439) and LaMnO_3_ (JCPDS-ICDD 75-0440) [68,69,70]. The presence of the rhombohedral LaNiO_3_ perovskite structure agrees with the tolerance factor values included in Table 5, which are not significantly modified by the presence of dopants, and the formation of Co-, Fe- and Mn-containing perovskites is justified by the higher *t* values of LaCoO_3_ (0.997), LaFeO_3_ (1.009) and LaMnO_3_ (0.993) perovskites [71]. Moreover, it is important to note that the L0.8NCo0.1 sample shows a slight shift in the main XRD signal towards higher 2θ values, probably because of a small modification of the LaNiO_3_ cell parameters as consequence of the insertion of Co in the structure, which occurs due to the similar ionic radii of Ni and Co [72].

**Figure 9:**
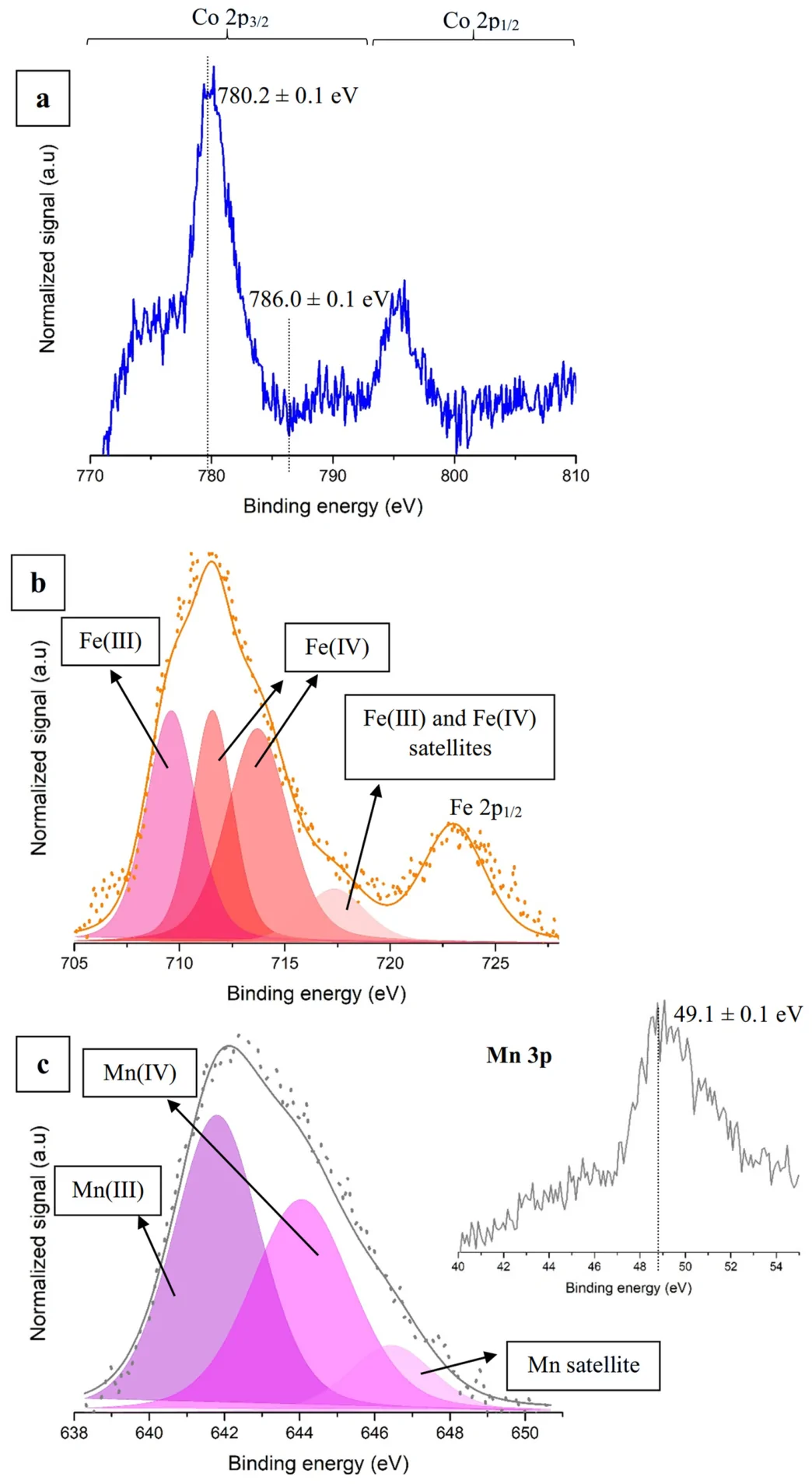
XPS spectra of Co 2p (**a**), Fe 2p (**b**) and Mn 2p^3/2^/Mn 3p transitions (**c**) of the fresh L0.8NM0.1 samples.

The relevant XPS data for these samples are featured in Figure 9 and Table 5. The Ni 3p transition reveals that Ni(II) and Ni(III) oxidation states coexist on the surface of all the samples and, according to the Ni(III)/Ni(II) ratios, the amount of Ni(III) is lower on the doped samples than on the L0.8N perovskite, as reported in the literature after the partial substitution of Ni by Fe [73,74]. Note that the partial substitution with Co causes a lower decrease in the amount of Ni(III) than that observed for the Fe- and Mn-doped samples, since these two metals could present higher oxidation states than Co (M(IV) or M(III)). Furthermore, the O_L_/(La + Ni(+M)) ratios are higher for the doped samples than for the L0.8N perovskite due to the contribution of the lattice oxygen from the dopant-based perovskite phases.

Regarding the dopants, Co 2p, Fe 2p and Mn 2p^3/2^ transitions were analyzed. For Co, the XPS Co(III)/Co(II) ratio is not usually estimated because the corresponding XPS signals are so close that they do not allow for an accurate identification of both oxidation states. Alternatively, it has been proposed that the presence of a Co 2p^3/2^ satellite peak at approximately 786 eV indicates that Co(II) is the sole oxidation state, whereas the absence of this peak suggests that Co(II) and Co(III) oxidation states coexist on the surface [75]. Thus, as the satellite peak is not found in the Co 2p spectrum displayed in Figure 9a, the Co(II) and Co(III) oxidation states seem to coexist on the surface of the sample.

On the other hand, Figure 9b shows the Fe 2p_1/2_ and Fe 2p^3/2^ core levels detected for L0.8NFe0.1. According to N. S. McIntyre and colleagues [76], the assignment of the different Fe contributions to the surface of mixed oxides is challenging, as the Fe 2p spectral profile depends on the chemical environment. However, for perovskite-type mixed oxides, the following assignment of the Fe 2p^3/2^ core level deconvolutions is highly accepted in the literature [77,78]: (i) the Fe(III) peak at a binding energy lower than that of Fe(IV), and (ii) the Fe(IV) and Fe(III) satellite peaks located at approximately 713 eV and 718 eV, respectively. Additionally, the irregular Fe 2p^3/2^ signal also suggests the presence of a Fe(IV) oxidation state. However, as this signal appears at a higher binding energy than expected, weaker interactions of this species with perovskite should exist.

Finally, the deconvolution of the Mn 2p^3/2^ core level (Figure 9c) reveals the presence of Mn(III) and Mn(IV) ions, with the area corresponding to the Mn(III) deconvolution being higher than that assigned to Mn(IV) peak. Note that the satellite peak corresponding to Mn(III) species (detected at higher binding energies) confirms the existence of this oxidation state [79,80,81]. Additionally, the Mn 3p signal location (49.1 ± 0.1 eV) seems to confirm that Mn(III) is the main oxidation state on the L0.8NMn0.1 surface.

**Figure 10:**
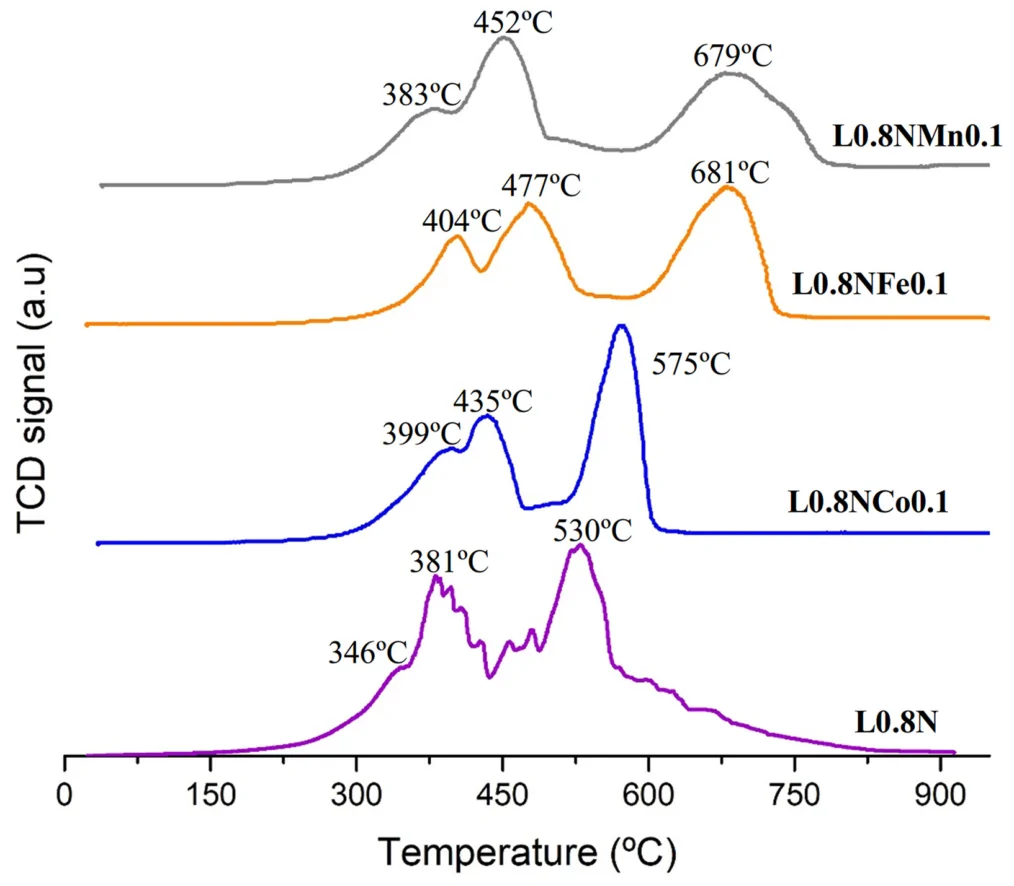
H_2_-TPR profiles of the fresh L0.8N and L0.8NM0.1 samples.

Figure 10 shows the H_2_-TPR profiles of the samples, including L0.8N as reference. As described in the previous section, the main H_2_ consumption peaks correspond to the reduction in the NiO and LaNiO_3_ phases. To identify the peaks corresponding to the possible reduction in the dopants, the following information was used: (i) the reduction of Co(III) to Co(II) in the perovskite lattice occurs between 400 °C and 450 °C, while the reduction in CoO particles (located on the surface) takes place at approximately 600 °C [82]; (ii) the reduction of Fe(IV) to Fe(III) is completed at 320 °C, that of Fe(III) to Fe(II) occurs between 420 °C and 740 °C, and the reduction in the FeO surface particles takes place at approximately 815 °C [83]; (iii) for Mn, the reduction of Mn(IV)/Mn(III) to Mn(II) is expected between 400 °C and 500 °C and, finally, the bulk Mn(III) species are reduced to Mn(II) at around 900 °C [84]. These data suggest that, for L0.8NM0.1 samples, the reduction to Co(0), Fe(0) and Mn(II) occurs at lower temperatures (owing to the Ni–dopant interaction) and, consequently, the corresponding peaks overlap with the reduction peak assigned to the formation of Ni/La_2_O_3_. However, both transformations of the perovskite structure, namely, that from the perovskite phase to the partially reduced one and from the latter to the Ni-M/La_2_O_3_ system (for Fe- and Co-doped samples) or to Ni-Mn_2_O_3_/La_2_O_3_ (for the Mn-doped formulation), are shifted towards higher temperatures compared to the L0.8N perovskite. This shift, previously reported in the literature [85,86], reveals that the reduction in Ni(II) is hindered by the presence of the dopants (mainly Fe and Mn) as a consequence of the strong Ni-M interaction. Note that the intensity of this interaction is expected to improve the stability of the metallic particles against sintering. Finally, to quantify the different contributions to the total H_2_ consumption listed in Table 6, the H_2_-TPR profiles were deconvoluted (see Figure S7) using the same criteria as for the LxN series, since a reliable differentiation of the reduction processes involving the dopants could not be achieved. The result shows that the V_NiO_ values of the doped samples are lower than that calculated for the undoped sample L0.8N, revealing a lower contribution from NiO species with low interactions with the perovskite phase. Additionally, the decrease in the XPS Ni(III)/Ni(II) ratios reported in the presence of the dopants seems to be attributable to charge compensation mechanisms in the perovskite phase, rather than to an increase in the amount of NiO species.

#### 2.2.2. Characterization of the L0.8NM0.1 Reduced Samples

**Figure 11:**
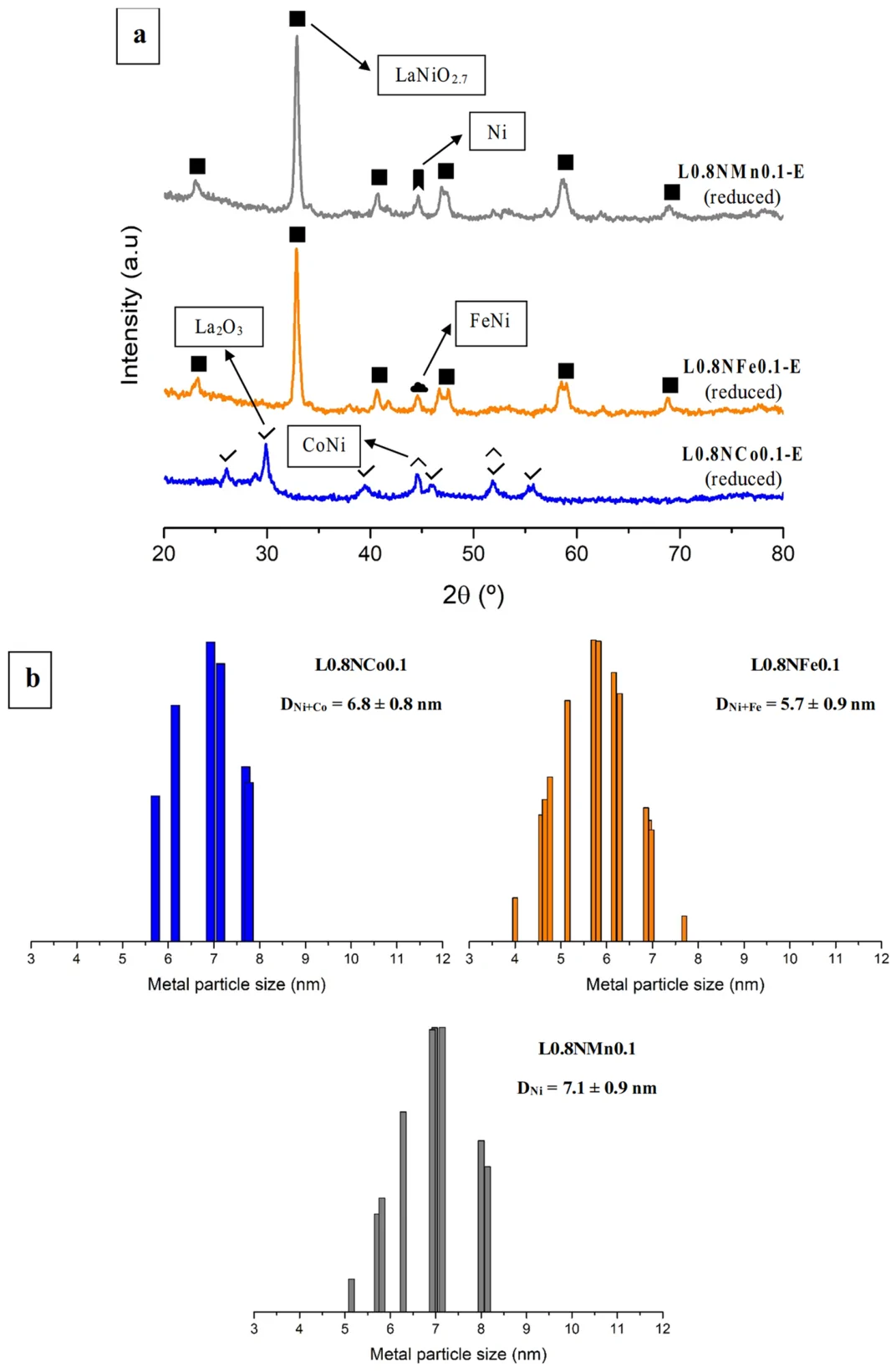
XRD patterns of the reduced L0.8NM0.1 samples (**a**), and metal particle size distribution, including the average particle sizes (D_Ni+M_) (**b**).

The XRD patterns of the reduced L0.8NM0.1 samples (under the conditions previously described for the LxN formulations), shown in Figure 11a, indicate that the Ni-Co/La_2_O_3_ system should be formed for the Co-containing sample. Since the Co reflections (JCPDS-ICDD 01-1255) overlap with those of the Ni phase, the CoNi alloy is expected to appear in the XRD profile [87,88]. Meanwhile, for the Fe- and Mn-doped samples, only a partial reduction to an oxygen-deficient perovskite (LaNiO_2.7_, JCPDS-ICDD 37-0928) supporting the Ni metallic particles is achieved. Additionally, for the reduced L0.8NFe0.1 sample, Fe(0) particles (identified as the FeNi phase, JCPDS-ICDD 03-1044) seem to be present. For L0.8NMn0.1, however, no Mn-based phases are observed in the XRD profile, which is likely attributable either to the formation of amorphous phases or to the Mn content being below the detection limit of this technique. The formation of the Ni-Co/La_2_O_3_ system in the L0.8NCo0.1 sample agrees with its higher reducibility, previously shown by H_2_-TPR (Figure 10).

Finally, the average metallic particle sizes, estimated by TEM (some representative TEM images are displayed in Figure S8 as examples) and included in Figure 11b, do not reveal a significant effect of the presence of dopants, as they are not significantly different from the value estimated for the L0.8N sample (7.4 nm), even though a narrower distribution of the average metallic particle sizes seems to be achieved for the doped samples.

#### 2.2.3. Catalytic Activity and Characterization of the L0.8NM0.1 Used Samples

**Figure 12:**
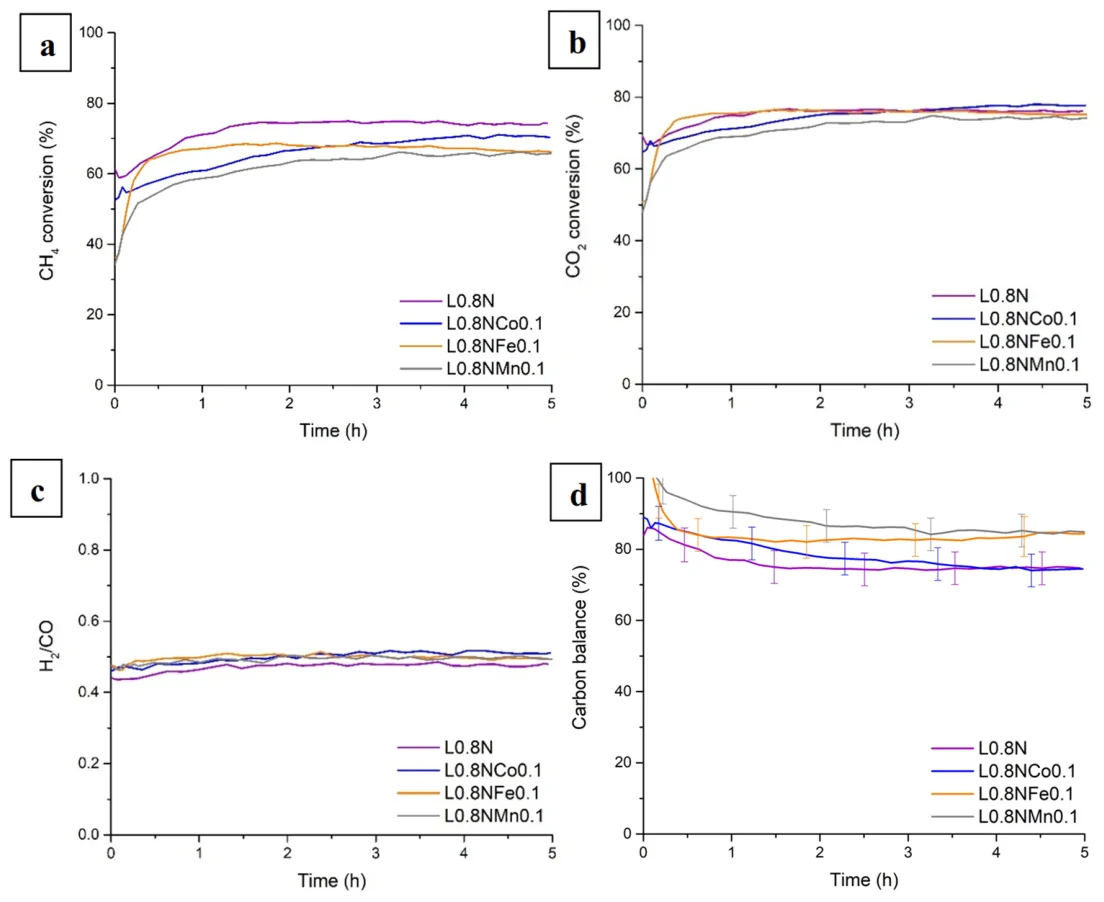
CH_4_ (**a**) and CO_2_ (**b**) conversions, H_2_/CO ratio (**c**) and carbon balance (**d**) during the DRM reaction at 700 °C (25:25 CH_4_:CO_2_) for the L0.8N and L0.8NM0.1 samples.

The results of the DRM tests at 700 °C under the 25:25 reactant atmosphere (25% CH_4_, 25% CO_2_ and 50% He) are compiled in Figure 12 and Table 7. In the presence of the dopants, the CO_2_ conversion is not significantly modified, but the CH_4_ conversion (and, consequently, the CH_4_-specific activity) is lower for the Fe- and Mn-doped samples [89,90], which is likely related to the decrease in the amount of Ni active sites, evidenced by the lower V_NiO_ values obtained by H_2_-TPR. The observed CH_4_ conversions for L0.8NCo0.1 are lower than those reported for the analogous Ni-Co/La_2_O_3_ bimetallic sample (CH_4_/CO_2_ = 1 at 800 °C) [91], and similar to the Ni-Fe/La_2_O_3_ formulations (CH_4_/CO_2_ = 1 at 750 °C) [92]. However, L0.8NMn0.1 overcomes the observed CH_4_ conversions found for Ni-Mn bimetallic samples at 700 °C (CH_4_/CO_2_ = 1) [93]. The ΔΧ_CH4_ and ΔΧ_CO2_ values (see Table S5) reveal an improved stability for the Fe-doped sample, which presents the most constant performance [94]. Note that the L0.8NCo0.1 sample presents higher CH_4_-specific activity than the undoped perovskite, which is likely attributable both to the participation of Co in the activation of methane and to the presence of a higher amount of reduced Ni species on the surface of this sample, as it is more reducible than the others. Moving to the reaction products, the H_2_ yield (which is lower than the thermodynamic value, at 82%, due to the effect of secondary reactions such as, mainly, the RWGS reaction) and the H_2_/CO ratio feature similar values for all the samples, which are lower than the values reported for analogous bimetallic samples [91,92,93]. Finally, for all the doped formulations, a decrease in the amount of deposited carbonaceous material is detected. L0.8NFe0.1 and L0.8NMn0.1 samples accumulate the lowest amount of carbon, in agreement with their lower catalytic activity, and, for the Fe-doped sample, also because the FeO_x_ species are able to decrease the amount of deposited carbonaceous material [95]. However, the value is still higher than those reported for the three analogous bimetallic formulations [91,92,93].

Finally, TEM analyses of the used samples (the corresponding TEM images are displayed in Figure S9) allow for the conclusion that the presence of the dopants does not prevent the sintering of metallic particles during the DRM reaction, as shown by the average particle sizes summarized in Table 8.

In conclusion, Co seems to be the most interesting dopant for the L0.8N formulation, as it allows for a certain increase in the CH_4_-specific activity and a decrease in the amount of deposited carbonaceous material during the DRM reaction. Consequently, in the next section, the optimization of the amount of Co (x) in the La_0.8_Ni_1−x_Co_x_O_3_ formulation will be explored.

### 2.3. Optimizing the Dopant Amount (x) in the La_0.8_Ni_1−x_Co_x_O_3_ Formulation

In this section, the samples will be denoted as L0.8NCox, with “x” representing the degree of substitution of Ni by Co.

#### 2.3.1. Characterization of the La_0.8_Ni_1−x_Co_x_O_3_ Fresh Samples

To optimize the cobalt content, two samples with different substitution degrees, La_0.8_Ni_0.75_Co_0.25_O_3_ and L_0.8_Ni_0.5_Co_0.5_O_3_ (denoted as L0.8NCo0.25 and L0.8NCo0.5, respectively), were synthesized, characterized and tested for the DRM reaction. The Co weight percentages, determined by μ-XRF and ICP-OES and included in Table 9, are consistent with the theoretical values. For the sample with the highest Co content, surface Co enrichment is suggested by its higher percentage, as determined by μ-XRF, compared to the bulk content (obtained by ICP-OES).

**Figure 13:**
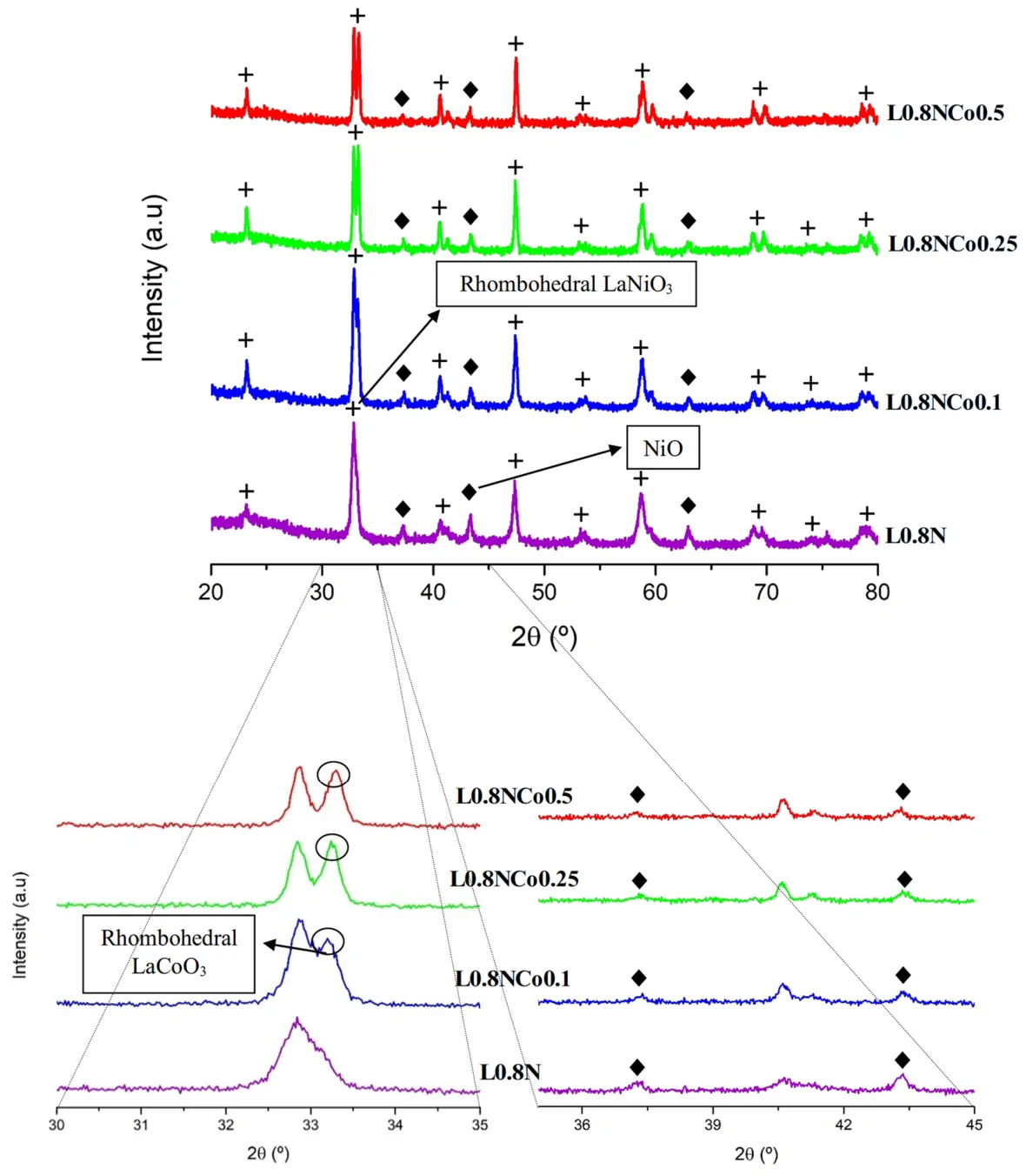
XRD patterns of the fresh L0.8N and L0.8NCox samples.

The XRD profiles featured in Figure 13 reveal that, similarly to L0.8NCo0.1, the rhombohedral LaNiO_3_ and LaCoO_3_ perovskite phases coexist with the NiO phase. Notably, as the Co content increases, the LaCoO_3_ peak intensity increases, whereas the intensity (and resolution) of the NiO signals decreases. The comparison between the tolerance factor of the samples (included in Table 9) seems to justify the stabilization of the LaCoO_3_ phase as the Co content increases, since the *t* value for the LaCoO_3_ phase (0.997) is closer to 1 (corresponding to the ideal cubic structure) than that for the LaNiO_3_ phase (0.989).

The XPS data for these samples are displayed in Table 9. Similarly to L0.8NCo0.1, the analysis of the Ni 3p profiles reveals the coexistence of Ni(II) and Ni(III) on the surface of the two samples with higher cobalt contents, and that Ni(III) is the most abundant oxidation state for the L0.8N and L0.8NCo0.25 samples. However, the Ni(III)/Ni(II) ratio does not follow a clear trend with the Co percentage, suggesting that these ratios are likely related to the Co(III)/Co(II) ratio, which was not estimated as the Co(III) and Co(II) peaks are too much close to obtain a reliable result. Nevertheless, a higher proportion of Co(II) is expected for L0.8NCo0.25, as it presents with more Ni(III) on the surface. Additionally, as the Co/(La + Ni + Co) XPS ratio becomes closer to the nominal one as the Co content increases, the insertion of Co into the lattice likely becomes more difficult as the cobalt content is higher. Finally, as the O_L_/(La + Ni(+Co)) XPS ratios are lower than the nominal value, oxygen vacancies should exist on the surface of all the samples.

**Figure 14:**
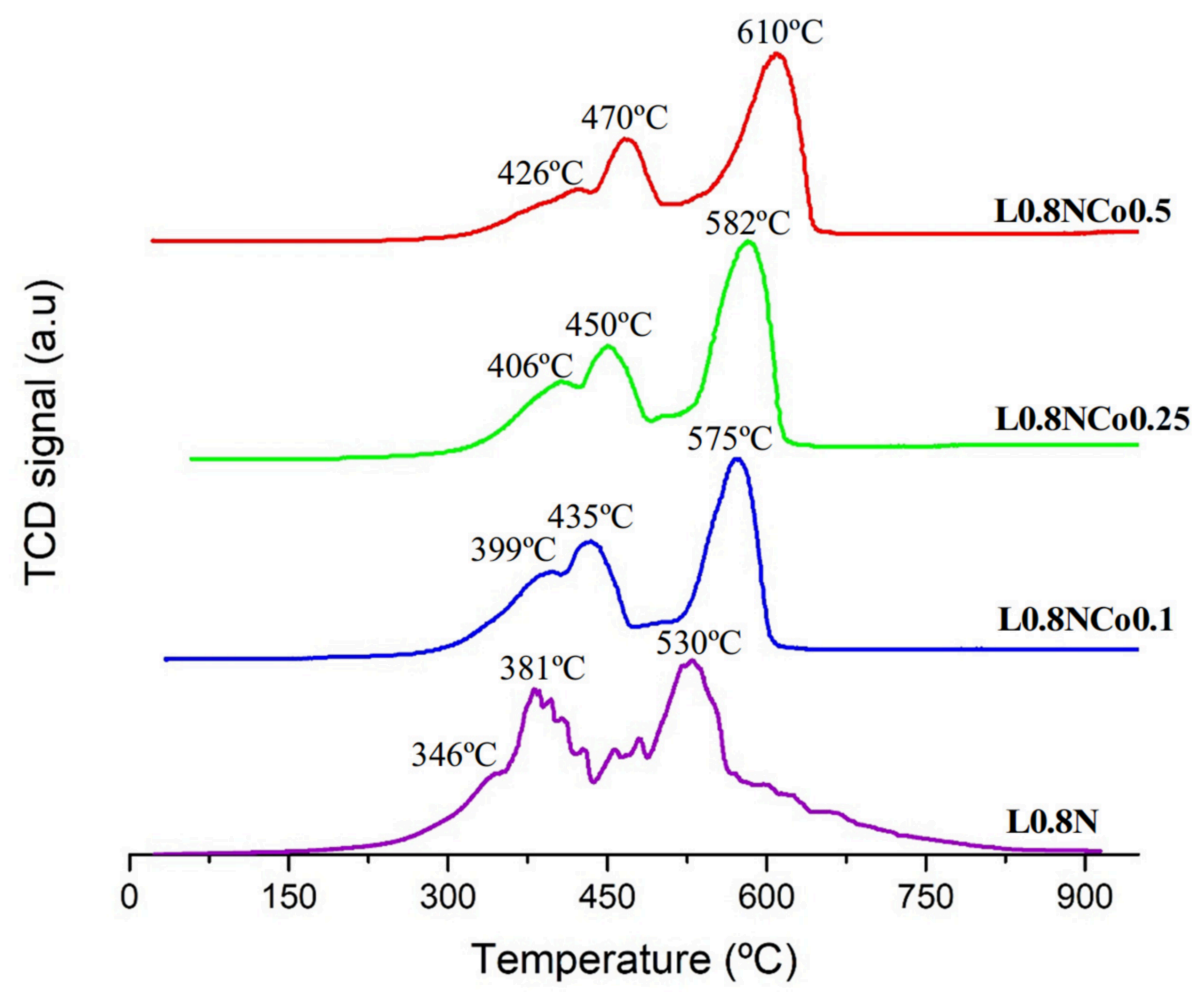
H_2_-TPR profiles of the fresh L0.8N and L0.8NCox samples.

The H_2_-TPR profiles shown in Figure 14 exhibit the expected peaks in the above-described Ni and Co redox processes. These profiles reveal a gradual increase in the temperature for all reduction processes (so the reducibility decreases) with the cobalt percentage, probably attributable both to an increase in the intensity of the Ni-Co interaction [96] and to the higher stability of the LaCoO_3_ perovskite [96]. This stronger Ni-Co interaction also seems to hinder the formation of NiO species, as shown by to the lower V_NiO_ volumes (Table 10) calculated after deconvoluting the corresponding H_2_-TPR profiles (see Figure S10).

#### 2.3.2. Characterization of the La_0.8_Ni_1−x_Co_x_O_3_ Reduced Samples

**Figure 15:**
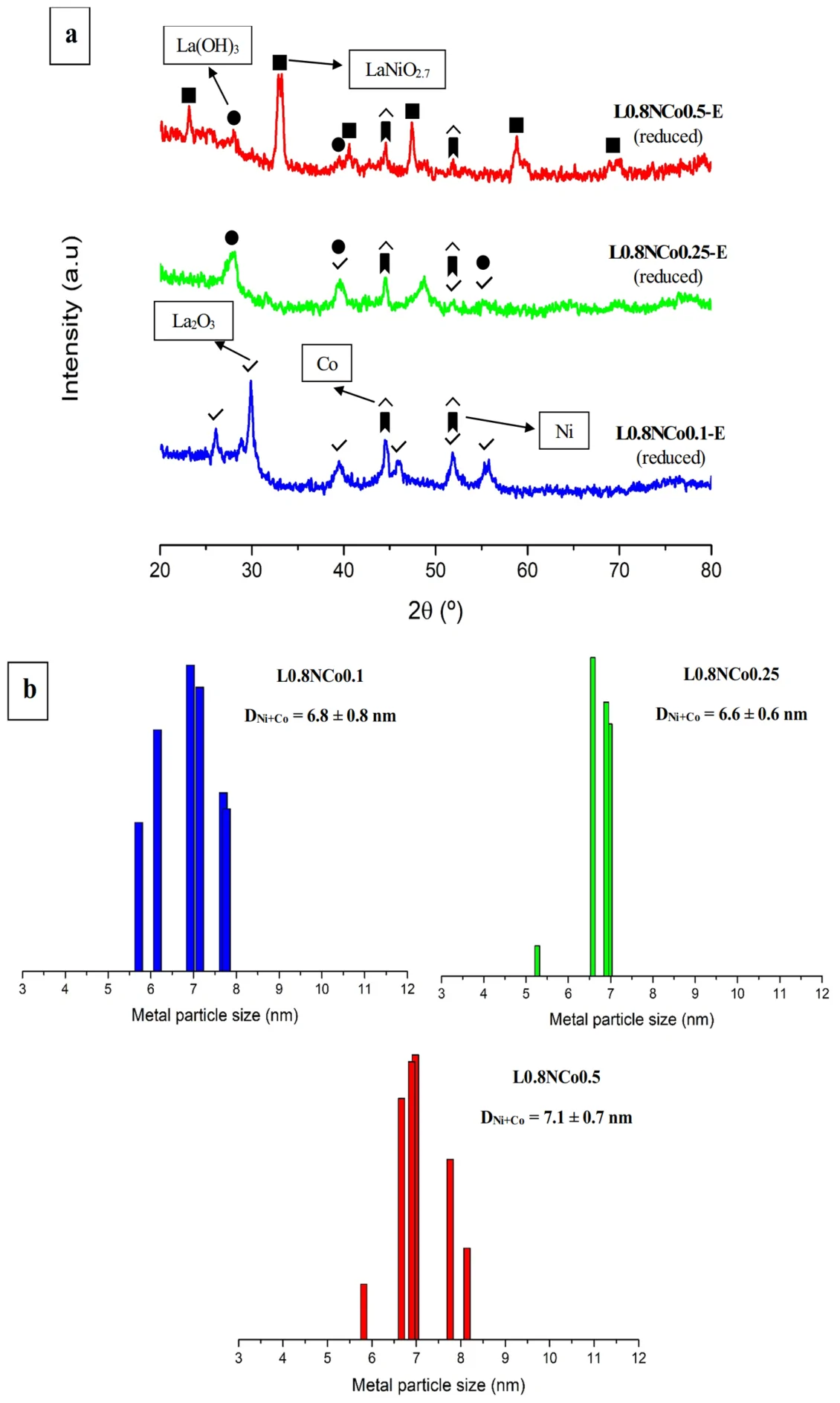
XRD patterns of the reduced L0.8NCox samples (**a**) and metal particle size distribution, including the average particle sizes (D_Ni+Co_) (**b**).

Figure 15a presents the XRD profiles of the reduced L0.8NCox samples. For the L0.8NCo0.25 sample, the main La_2_O_3_ XRD signal is not detected, whereas the other peaks expected for this phase are present, suggesting, as already stated in the related literature, that the main La_2_O_3_ XRD signal is the most affected by the change in the composition [97,98]. Thus, the L0.8NCo0.25 sample likely consists of a Ni-Co/La_2_O_3_ system that coexists with the crystalline La(OH)_3_ phase. However, for the L0.8NCo0.5 sample, partially reduced LaNiO_2.7_ perovskite is detected, in agreement with its lower reducibility (see H_2_-TPR profiles). Finally, as revealed by the data in Figure 15b, even though the average metallic particle size of the reduced samples does not seem to be significantly modified by the Co content, the L0.8NCo0.25 sample presents the narrowest distribution of particle sizes (as examples, several TEM images are shown in Figure S11).

#### 2.3.3. Catalytic Activity and Characterization of the La_0.8_Ni_1−x_Co_x_O_3_ Used Samples

**Figure 16:**
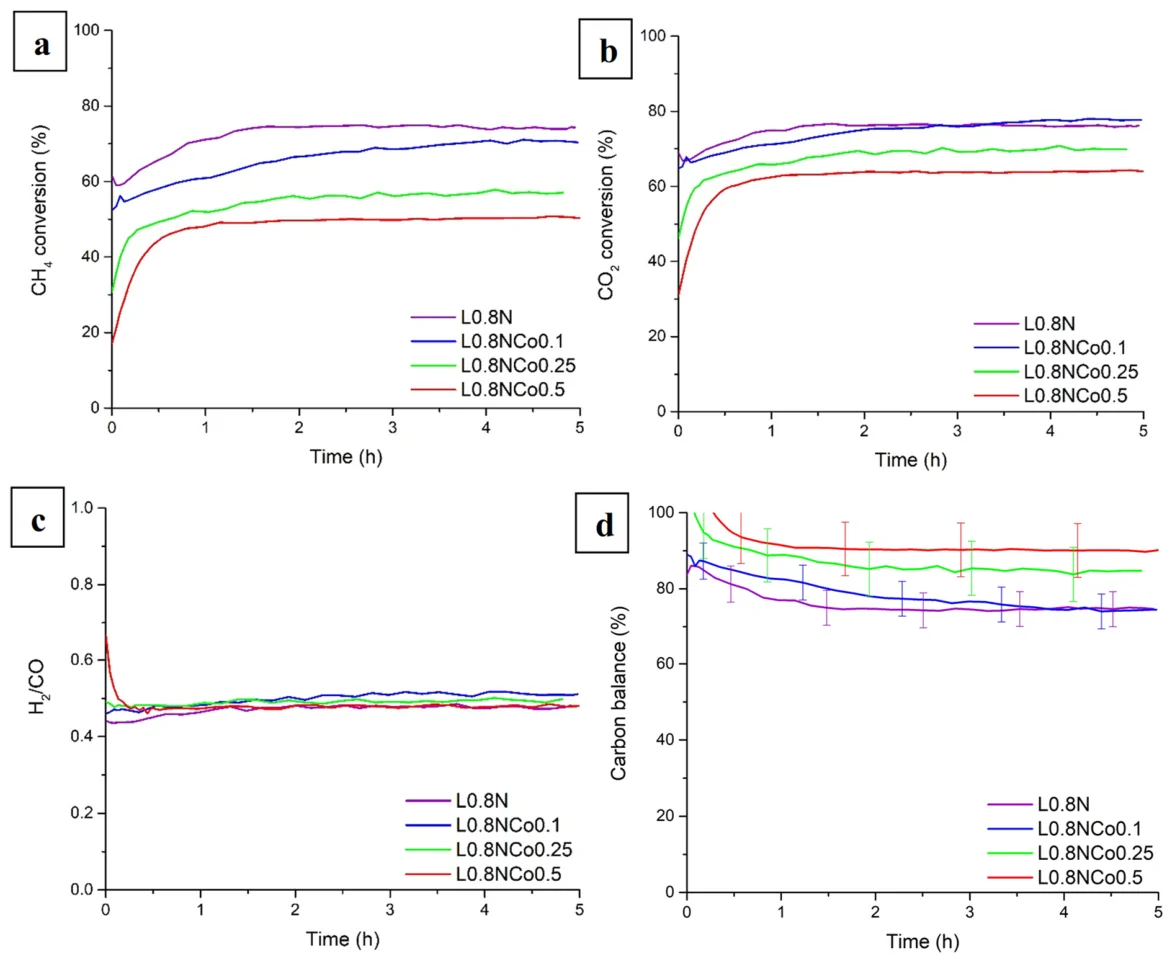
CH_4_ (**a**) and CO_2_ (**b**) conversions, H_2_/CO ratio (**c**) and carbon balance (**d**) during the DRM reaction at 700 °C (25:25 CH_4_:CO_2_) for the L0.8N and L0.8NCox samples.

Table 11 and Figure 16 compile the catalytic data for the DRM reaction at 700 °C (under a 25% CH_4_, 25% CO_2_, 50% He (25:25) reactant atmosphere) for the three Co-containing samples. A significant decrease in the conversion of the reactants and in the H_2_ yields is registered for the two samples with higher cobalt percentages. Additionally, the difference between the CH_4_ and CO_2_ conversions increases, suggesting that the RWGS reaction is being promoted. In the literature [31,99], it is reported that the presence of a high amount of cobalt hinders methane activation [31,86] because the energy for the Co-CH_4_ interaction (2.0 kcal mol^−1^) is lower than that corresponding to the Ni-CH_4_ interaction (3.2 kcal mol^−1^) [99]. However, as the ΔΧ_CH4_ and ΔΧ_CO2_ values (Table S5) for the L0.8NCo0.25 and L0.8NCo0.5 samples are lower than those for L0.8NCo0.1, both samples present a more stable catalytic performance. Finally, as expected from the lower CH_4_ conversions achieved for the L0.8NCo0.25 and L0.8NCo0.5 samples, the amount of deposited carbonaceous material is substantially lower. This is further supported by their near-100% carbon balance values and by the carbon amounts determined from the area under the molar carbon balance profiles (see Table S5 and Section 3 for details). The comparison of these results with other studies confirms that L0.8NCo0.25 and L0.8NCo0.5 formulations feature an improved resistance against carbon deposition than Ni-Co/La_2_O_3_ formulations [91]. In the literature, it is proposed that the high affinity of Co for CO_2_ promotes its activation [100,101], favoring the gasification of the carbon material generated during the reaction. Note that the improvement in the carbon deposition resistance overcomes the values reported for LaNi_1−x_Co_x_O_3_ formulations (*x* near 0.25 or 0.5) with a similar substitution degree of Ni by Co, but preserving the original La stoichiometry [31,102].

Finally, the average metallic particle sizes obtained from TEM data for the used samples (included in Table 12 and, as examples, representative TEM images are shown in Figure S12) reveal that the metallic particles are more resistant to sintering in the samples with higher Co contents. This suggests improved Ni-Co interactions, which are also likely to contribute to the lower amount of deposited carbonaceous material registered [103,104].

Thus, as the L0.8NCo0.25 formulation presents a higher conversion of reactants (and higher CH_4_-specific activity) than L0.8NCo0.5, and a negligible amount of deposited carbonaceous material, this sample was selected to be tested in the DRM reaction under a simulated biogas reactant atmosphere.

**Figure 17:**
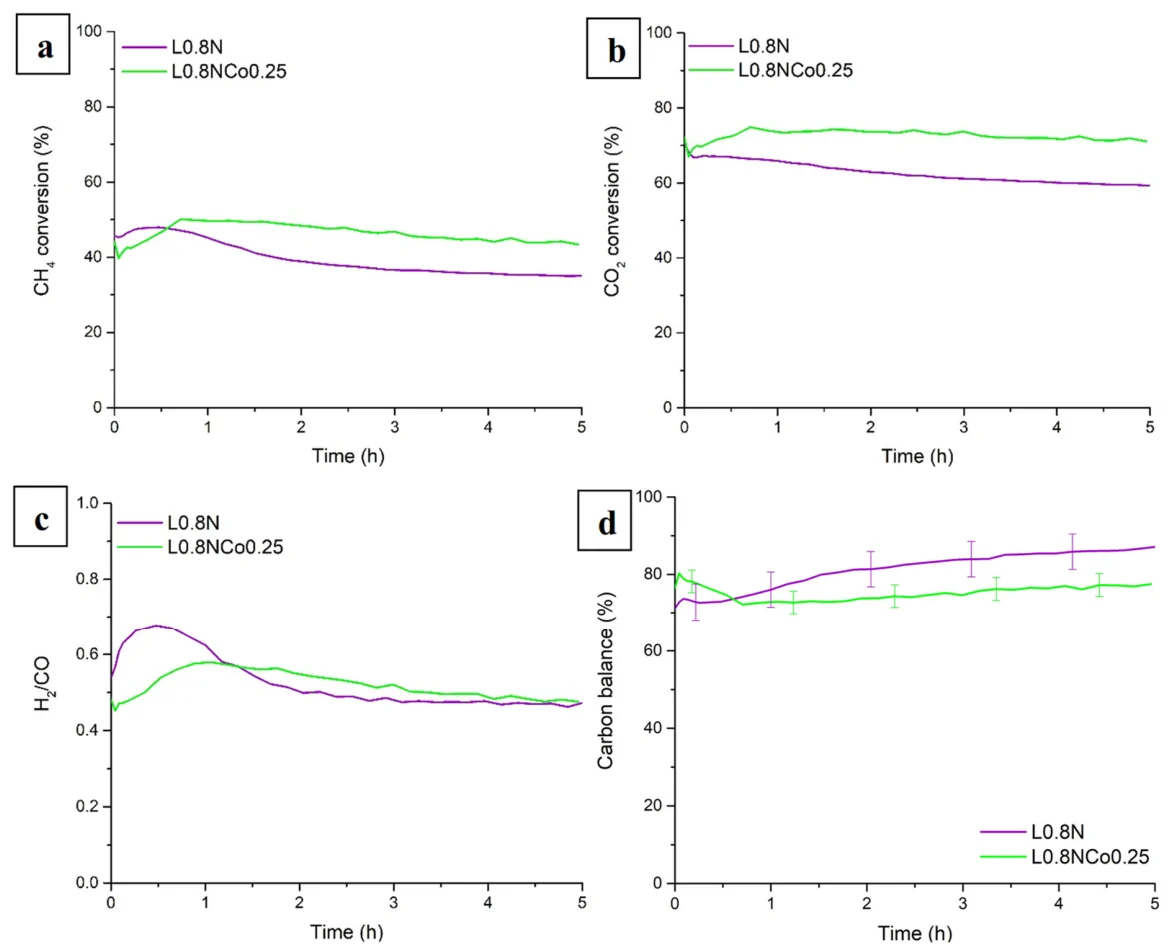
CH_4_ (**a**) and CO_2_ (**b**) conversions, H_2_/CO ratio (**c**) and carbon balance (**d**) during the DRM reaction at 700 °C (65:35 CH_4_:CO_2_) for the L0.8N and L0.8NCo0.25 samples.

The results displayed in Table 13 and Figure 17 reveal that, as expected from the high CH_4_/CO_2_ ratio of the simulated biogas reactant atmosphere (65% CH_4_, 35% CO_2_, denoted as 65:35, and without using He as a balance gas), CH_4_ conversion is lower (43%) than for the 25:25 composition, whereas CO_2_ conversion is similar (71%, which is almost coincident with the equilibrium value (70%)). Thus, the widening difference between the two reactant conversions is likely attributable not only to the lower CO_2_ percentage (which is the limiting reactant) but also to the strong promotion of the RWGS reaction under the simulated biogas reactant atmosphere. Note that both reactant conversions are lower than those found for a Ni-Co/La_2_O_3_ bimetallic formulation tested at 800 °C under a reactant atmosphere with a CH_4_/CO_2_ ratio of 1.5 [91] and, also, to those reported for Pt- and Ru-supported catalysts (CH_4_/CO_2_ = 2 at 650 °C) [65]. On the other hand, the H_2_/CO ratio is similar to that obtained for the 25:25 composition (see above) because CO is also produced by the RWGS side reaction. Additionally, since the amount of CO_2_ is lower, the methane decomposition is favored and, consequently, the CH_4_-specific activity and the amount of carbonaceous material increase in comparison to the values registered for the 25:25 atmosphere. Thus, even though the L0.8NCo0.25 sample exhibits improved stability for the metallic particles (the average metallic particle size for the used L0.8NCo0.25 sample, determined by TEM, was 7.3 ± 0.9 nm; see Figure S13 for a representative TEM image), the high amount of deposited carbonaceous material after the DRM reaction under simulated biogas conditions remains the primary drawback of this formulation.

In summary, the L0.8NCo0.25 formulation, which combines both compositional modifications tested, seems to be the best sample of the three previous analyzed series, as it features the following improvements: (i) the La deficiency (x = 0.8) caused the formation of slightly smaller Ni particles than LaNiO_3_, (ii) the partial substitution of Ni by Co improved the stability of the metallic particles during the catalytic tests, and (iii) the increase in the Ni substitution degree (corresponding to L0.8NCo0.25 formulation) further increased the resistance against sintering, minimizing the production of carbonaceous deposits.

### 2.4. Exploring the Catalytic Performance of L0.8NCo0.25 for DRM Under More Realistic Conditions

#### 2.4.1. Effect of the Operating Regime

**Figure 18:**
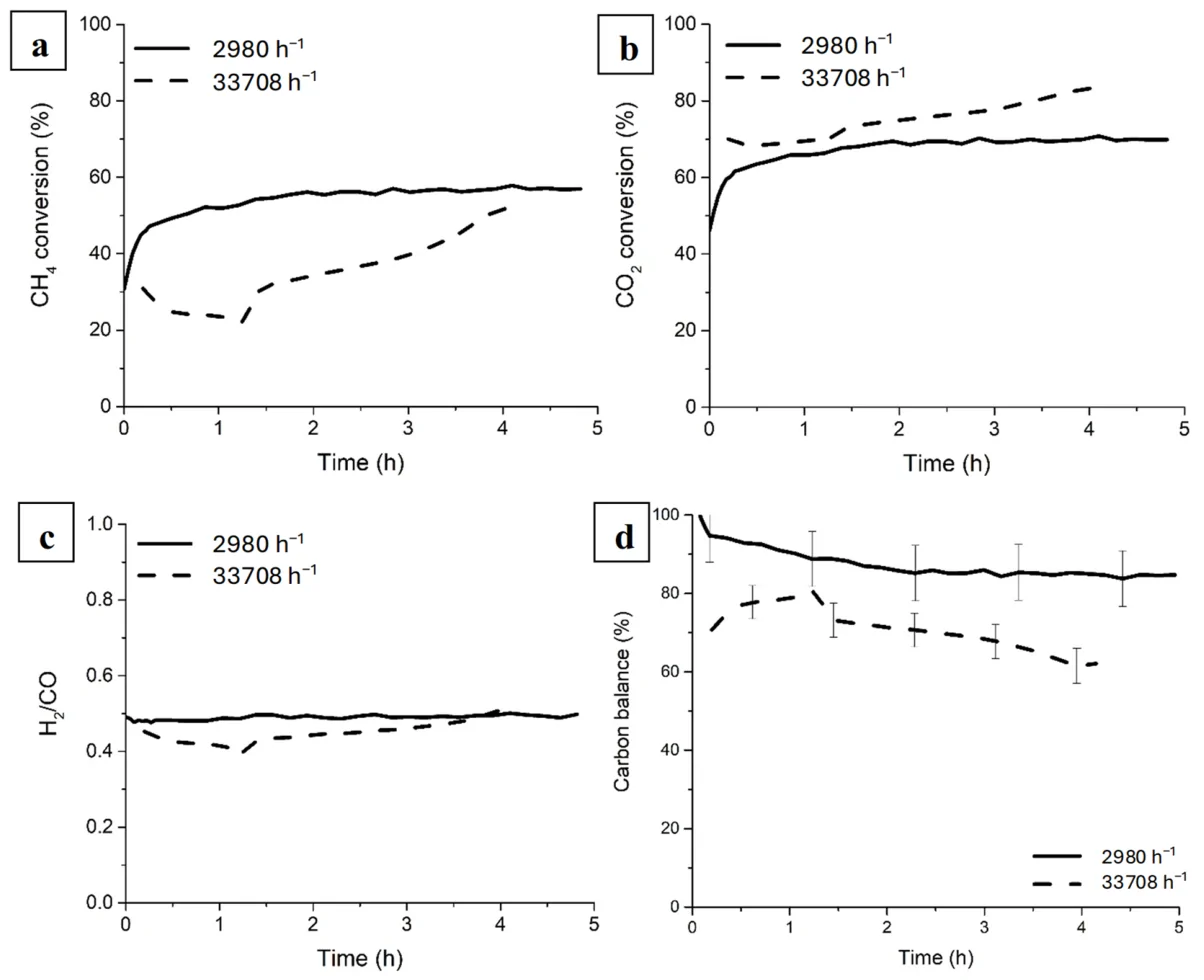
CH_4_ conversion (**a**), CO_2_ conversion (**b**), H_2_/CO ratio (**c**) and carbon balance (**d**) during the DRM reaction at 700 °C (25:25 CH_4_:CO_2_) under different operating regimes (low-GHSV (2980 h^−1^) and high-GHSV (33,708 h^−1)^).

To explore its potential for industrial application, in this section the catalytic performance of the L0.8NCo0.25 sample (as catalyst precursor for the DRM reaction) under high Gas Hourly Space Velocity (GHSV) conditions is analyzed [105,106]. However, it should be noted that the experiments at different GHSVs not only involved the use of different contact times but also different reactor configurations and the use (or absence) of SiC as a diluent. Consequently, the catalytic trends should be a consequence of these different operating regimes and should not be attributed solely to GHSV variation. Accordingly, L0.8NCo0.25 was tested under two operating regimes (described in detail in the Materials and Methods section) labeled with their respective GHSV values (33,708 h^−1^ and 2980 h^−1^). The relevant catalytic data obtained under the 25:25 reactant atmosphere, displayed in Table 14 and Figure 18, reveal a pronounced effect on catalytic performance upon a shift in the global operating conditions. This effect can be attributed not only to the shorter contact time between the reactants and the catalyst, which makes activation less effective [107], but also to the larger temperature gradients in the absence of SiC [108]. Firstly, focusing attention on the conversion profiles of the reactants, the difference between the CO_2_ and CH_4_ conversions increases under the high-GHSV configuration, suggesting a more relevant impact of the RWGS reaction, as was also concluded by Y. J. Su et al. using a CH_4_/CO_2_ ratio equal to 1 and a GHSV value of 10,000 h^−1^ [56]. It is important to note that although the high-GHSV configuration yields a slightly lower CH_4_ conversion, the CH_4_-specific activity increases. This surprising result is caused by the higher influence of the increased inlet molar CH_4_ ratio. The increased relevance of the RWGS reaction in the high-GHSV configuration is further supported by the low H_2_/CO ratio and H_2_ yield registered under these conditions. Additionally, a significant increase in the amount of carbonaceous material deposited was detected [109,110,111,112], consequently clogging the reactor and stopping the flow of reactants (see Figure 18).

**Figure 19:**
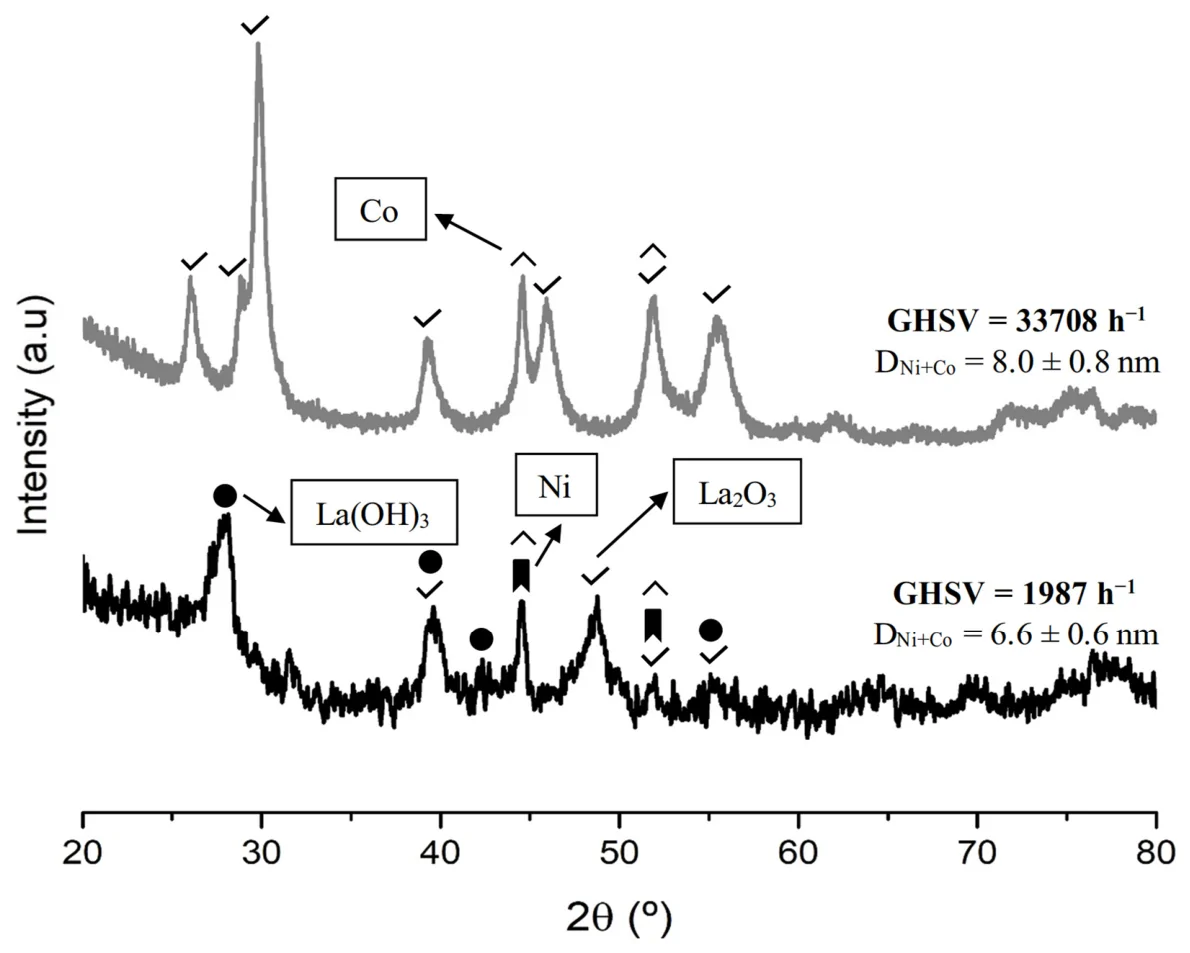
XRD patterns and average metal particle size of the L0.8NCo0.25 sample reduced in the different operating regimes.

The different catalytic performance of L0.8NCo0.25 in the high-GHSV configuration is also likely attributable to the different conditions used in the reduction in the sample (GHSV of 33,708 h^−1^ versus 1987 h^−1^). To check this hypothesis, the sample reduced under the high-GHSV conditions was analyzed by XRD and TEM, with the diffractograms and the average particle sizes being compared in Figure 19 using the data related to the sample reduced applying the low-GHSV configuration. Thus, after the reduction step in the high-GHSV system, the La_2_O_3_ peaks are more intense, whereas the peaks assigned to the La(OH)_3_ phase disappear, as concluded by G. P. Figueredo et al. [107] for Ni/LaAlO_3_ and Ni/α-Al_2_O_3_ formulations. In the literature, it is reported that the formation of the La(OH)_3_ phase is hindered at high GHSVs, since it is formed through the interaction between water and La_2_O_3_, which is reduced under these conditions [113]. This finding supports the previously described hypothesis, as it was proved that the La(OH)_3_ phase promotes the removal of the carbonaceous deposits because hydroxyl groups hinder the transformation of CH_x_ intermediates into C_x_ species [114]. So, the increase in the amount of carbonaceous material accumulated during the DRM test is likely attributable to the absence of the La(OH)_3_ phase after the reduction step in the high-GHSV configuration. This interpretation is further supported by the resistance against carbon deposition found for hydroxyapatite-based formulations, in which hydroxyl groups react with CO_2_ to produce formiates and oxycarbonates that promote the removal of carbonaceous deposits [115].

As a final remark, the main drawback of using the L0.8NCo0.25 formulation for the DRM reaction in the high-GHSV configuration seems to be the pronounced increase in the amount of carbonaceous material accumulated. However, future works are needed in order to decouple the effects of the different variables affecting the operating regime.

#### 2.4.2. Oxidative Dry Reforming of Methane

**Figure 20:**
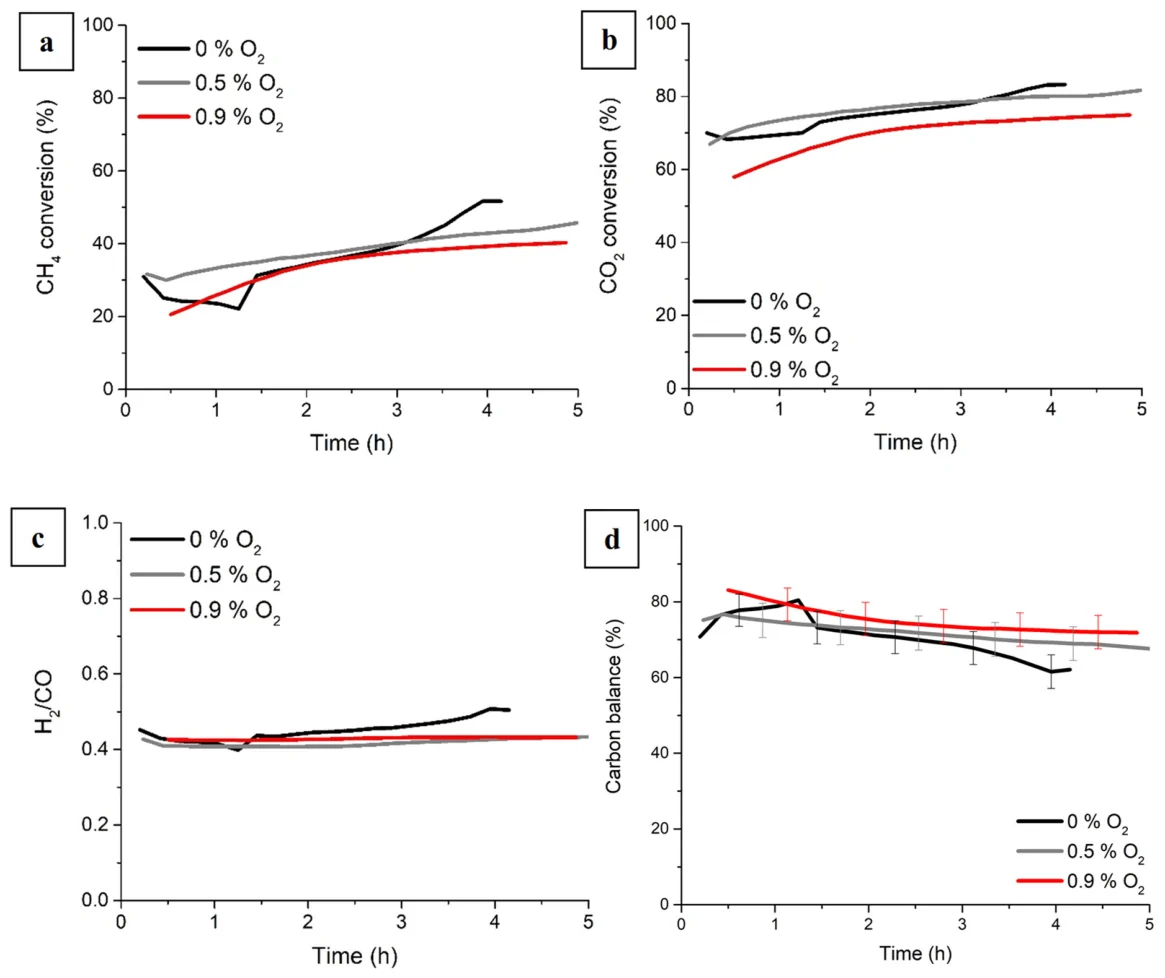
CH_4_ (**a**) and CO_2_ (**b**) conversions, H_2_/CO ratio (**c**) and carbon balance (**d**) for the ODRM reaction at 700 °C under the 25:25 reactant atmosphere using different O_2_ percentages (GHSV = 33,708 h^−1^).

As stated in the Introduction section, in the presence of oxygen in the reactant atmosphere (ODRM process), the amount of deposited carbonaceous material decreases in comparison to that found under DRM conditions due to its removal by gasification with oxygen. Thus, considering that a certain amount of O_2_ is present in real biogas [116], two representative oxygen percentages (0.5 and 0.9%) were added to the 25:25 reactant atmosphere (the gaseous mixtures are balanced with 49.5% and 49.1% He, respectively). Under these conditions, the catalytic activity of the L0.8NCo0.25 formulation at 700 °C using the high-GHSV configuration was evaluated; the results are shown in Table 15 and Figure 20.

First, the conversion profiles, which are similar to those recorded for the DRM test, reveal that the ODRM reaction is still ongoing after 4 h on stream, as the flow of reactants is sustained due to the continuous supply of O_2_ [117,118,119]. Note that the CH_4_ conversion decreases as the O_2_ percentage increases, likely due to the oxidation of the Co metallic particles [18,112,120,121], as this metal is more easily oxidized than Ni [122,123]. In the literature, the opposite trend (namely, increased CH_4_ conversions in the presence of oxygen) has been reported for noble metal catalysts (Pt, Ru and Rh), which is attributed to their resistance to oxidation [59]. On the other hand, as the H_2_/CO ratios and H_2_ yields are not significantly modified in the presence of O_2_, the RWGS reaction still takes place under ODRM conditions. Thus, even though the reactant conversions are somewhat lower, the presence of O_2_ significantly decreases the amount of deposited carbonaceous material after 5 h of ODRM reaction under the high-GHSV configuration.

**Figure 21:**
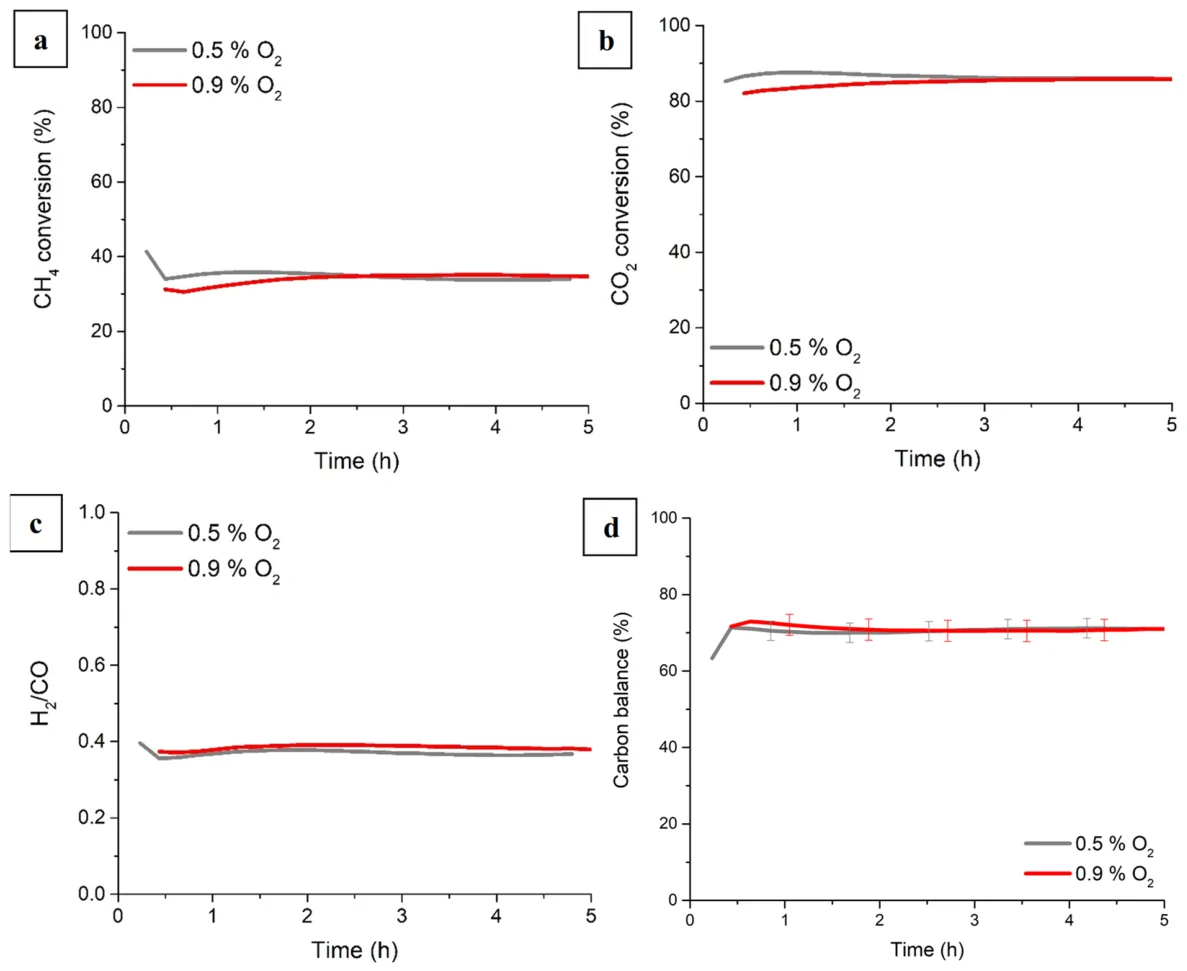
CH_4_ (**a**) and CO_2_ (**b**) conversions, H_2_/CO ratio (**c**) and carbon balance (**d**) for the ODRM reaction at 700 °C under the simulated biogas reactant atmosphere using different O_2_ percentages (GHSV = 33,708 h^−1^).

Considering these results, the effect of oxygen under simulated biogas conditions was explored. In this reactant atmosphere, the addition of O_2_ implies a decrease in the CH_4_ and CO_2_ percentages, resulting in 58.5:31.5:0.5 (9.5% He as balance gas) and 53.4:28.7:0.9 (17% He as balance gas) compositions. These volume percentages were calculated, keeping the CH_4_/CO_2_ ratio equal to 1.67). The data corresponding to the ODRM tests are presented in Figure 21 and Table 16. Note that, to the best of the author’s knowledge, this is the first time that the ODRM reaction was carried out under a simulated biogas atmosphere using a perovskite-type sample as a precursor of the active phase. As for the 25:25 reactant atmosphere, the reaction works over the 5 h on stream without interruptions and the reactants’ conversion percentages, the H_2_/CO ratio and the H_2_ yield are almost similar, whereas the amount of deposited carbonaceous material is lower for the reactant atmosphere containing the highest percentage of oxygen.

Finally, as the data shown in Figure S14 and Table S4 demonstrates, and in agreement with the results discussed above, a further decrease in the amount of deposited carbonaceous material can be achieved if the ODRM process under simulated biogas conditions is carried out in the low-GHSV configuration.

#### 2.4.3. Regeneration of the L0.8NCo0.25 Formulation in the ODRM Reaction

The results in the previous section show that, despite the presence of oxygen in the reactant atmosphere, the accumulation of carbonaceous material remains a limitation for the use of the L0.8NCo0.25 sample in the ODRM reaction under conditions close to those found in a potential real application (simulated biogas under a high-GHSV regime). Thus, to overcome this limitation, the use of a regeneration step after the DRM/ODRM reaction to remove the accumulated carbonaceous material by gasification with CO_2_ was included in the reaction scheme [124,125,126,127]. CO_2_ was selected because, even though O_2_ would be more efficient to remove the carbonaceous deposits, the high exothermicity of the gasification would provoke hot spots that could cause significant sintering of the metallic particles [128]. This problem is likely minimized when using a soft oxidant, such as CO_2_ [129], which promotes the reverse Boudouard equilibrium [130].

**Figure 22:**
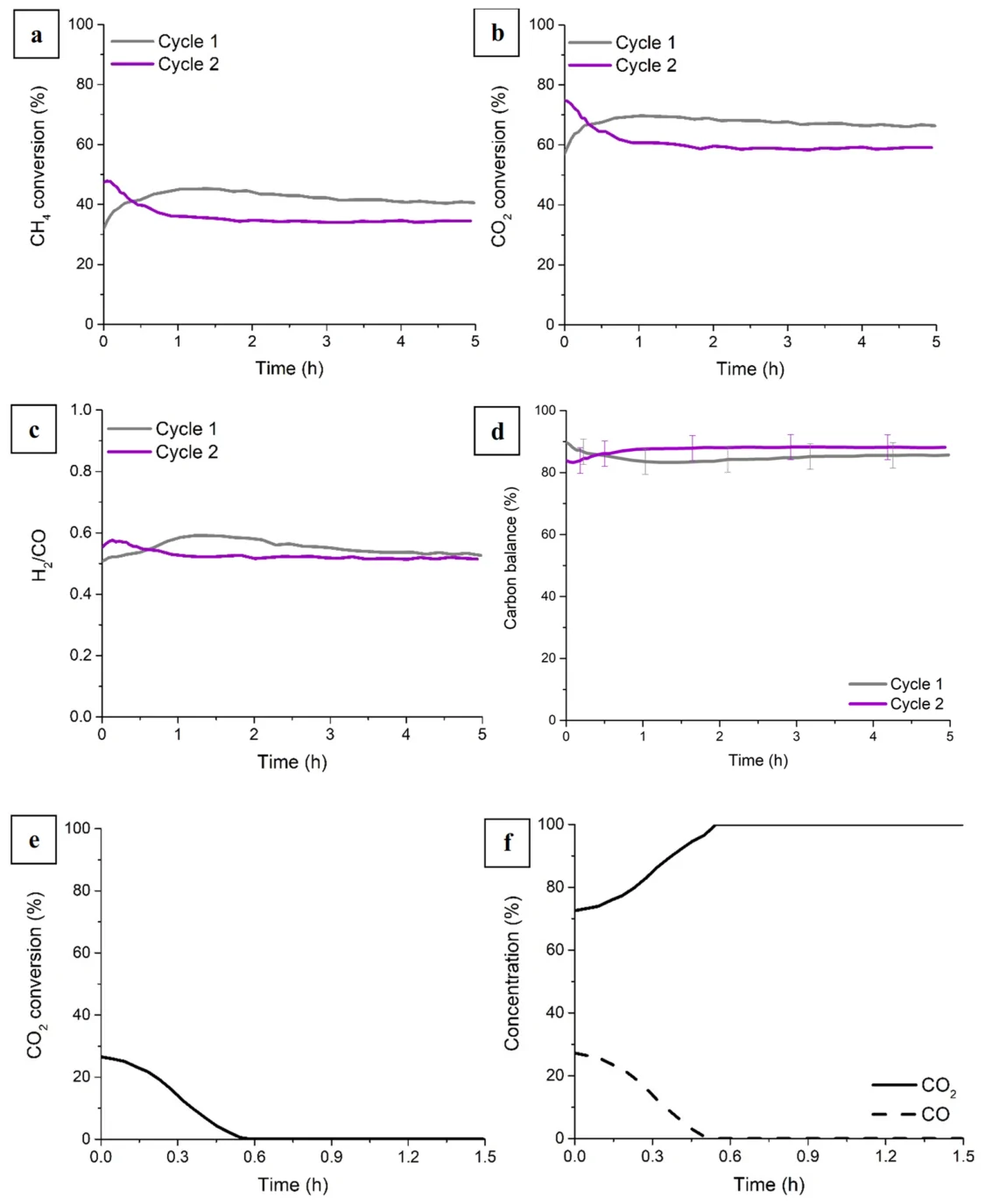
Effect of the regeneration step (pure CO_2_, 700 °C, 1.5 h) in the ODRM reaction: CH_4_ (**a**) and CO_2_ (**b**) conversions, H_2_/CO ratio (**c**), carbon balance (**d**), CO_2_ conversion during regeneration (**e**), and CO/CO_2_ profiles during regeneration (**f**).

The regeneration test was performed using pure CO_2_ at 700 °C for 1.5 h after the ODRM reaction in the presence of the L0.8NCo0.25 sample, using the simulated biogas reactant atmosphere containing 0.5% O_2_ and a low-GHSV configuration. Considering that the sample has to be reduced before the ODRM reaction, the sequence of steps to develop is: 1st step: first reduction → 2nd step: first ODRM cycle → 3rd step: regeneration with CO_2_ → 4th step: second reduction → 5th step: second ODRM cycle. This sequence will determine the effectiveness of the regeneration step by comparing the data of the first and second ODRM cycles. The data are shown in Table 17 and Figure 22 and, additionally, Figure 22e,f display the CO_2_ conversion and the CO and CO_2_ evolution profiles registered during the regeneration step. Note that, as the CO_2_ consumption decreased to zero and the production of CO stopped after 30 min of the regeneration time, it seems that the total removal of the deposited carbonaceous material during the first ODRM cycle was achieved during the regeneration step. The reactants’ conversion and the H_2_ production recorded during the second ODRM cycle are lower than those achieved during the first ODRM cycle, but the amount of deposited carbonaceous material notably decreased (more than five times). Thus, after the regeneration step with CO_2_, the sample is likely more resistant to the accumulation of carbonaceous material. Two reasons seem to explain this fact: (i) the CH_4_ conversion is lower during the second ODRM cycle (and therefore also the amount of carbon generated) and (ii) the sintering of the metallic particles takes place to a lower extent during the second cycle, as revealed by the average particle size of the used samples in comparison to those of the reduced one (that is, 6.6 ± 0.6 nm): 9.9 ± 0.1 nm and 10.3 ± 0.7 nm for the first and second cycles, respectively (representative TEM images are included in Figure S15). Thus, the increase in average particle size is much lower during the second ODRM cycle (from 9.9 nm to 10.3 nm) than during the first one (from 6.6 nm to 9.9 nm).

In summary, the use of a regeneration step with pure CO_2_ at 700 °C after the ODRM reaction allows for the removal of the deposited carbonaceous material during the reaction, offering a new potential application for CO_2_ that will contribute to the revalorization of biogas into valuable products such as H_2_ or syngas.

## 3. Materials and Methods

### 3.1. Synthesis and Characterization

La_x_NiO_3_ and La_x_Ni_1−y_M_y_O_3_ samples (denoted as LxN and LxNMy, with M = Co, Fe or Mn) were synthesized by the sol–gel method adapted to aqueous medium [131,132]. First, the metal precursors (lanthanum nitrate hydrate (La(NO_3_)_3_ · H_2_O, Apollo Scientific, 99.9 wt%), nickel(II) nitrate hexahydrate (Ni(NO_3_)_2_ · 6 H_2_O, Panreac, 98.0 wt%), cobalt(II) nitrate hexahydrate (Co(NO_3_)_2_ · 6 H_2_O, Panreac, 98.0 wt%), iron(III) nitrate nonahydrate (Fe(NO_3_)_3_ · 9 H_2_O, Sigma-Aldrich, 98.0 wt%) and manganese(II) nitrate tetrahydrate (Mn(NO_3_)_2_ · 4 H_2_O, Sigma-Aldrich, 99.0 wt%)) were added, under continuous stirring, to a solution of EDTA (C_10_H_16_N_2_O_8_, Sigma-Aldrich, St. Louis, MI, USA, 98.5 wt%) at 60 °C, with the amount of chelating agent corresponding to a 1:2 molar Ni:EDTA ratio. Subsequently, citric acid (C_6_H_8_O_7_, Sigma-Aldrich, 99.0 wt%) was incorporated in the same molar ratio used for EDTA and, after total dissolution of the reactants, the temperature increased to 80 °C to achieve gel formation. During synthesis, the pH was maintained at 9 using an ammonia solution (NH_3_, Panreac, Castellar del Valles, Spain, 30 wt%). Finally, the obtained gel was dried at 150 °C for 12 h and calcined at 850 °C for 6 h (the calcination process was carried out in static air under ambient atmospheric conditions and using a heating rate of 5 °C min^−1^).

The characterization of the samples was carried out using the following techniques:

- The surface composition of the fresh formulations was determined by μ-XRF, using an Orbis Micro-XRF Analyzer from EDAX.
- Bulk Co, Fe and/or Mn contents for the La_x_Ni_1−y_M_y_O_3_ samples were obtained by ICP-OES using the Perkin-Elmer device model Optimal 4300 DV. The extraction of the metals for these analyses consisted of mineralization of the samples with a diluted aqua regia solution at 60 °C and stirring for 1 h.
- A crystalline structure of the samples (fresh and reduced) was obtained using XRD. The X-ray profiles were obtained between 20° and 80° 2θ angles with step rates between 0.4 and 3.4° min^−1^ using Cu K_α_ (0.15418 nm) radiation in a Bruker D8-Advance device.
- The morphology of selected reduced samples was analyzed by FE-SEM, using a ZEISS Merlin VP Compact device. To obtain the FE-SEM images, a voltage of 2 kV (resolution of 1.5 nm) was applied.
- To identify the metallic particles in the reduced and used samples, and to estimate their average sizes, TEM was employed using a JEOL model JEM-1400 Plus microscope provided with a GATAN model ORIUS image acquisition camera. To obtain the particle size distributions, 300 particles were used.
- A surface characterization of the fresh and some selected reduced samples was conducted by XPS, using a K-Alpha Photoelectron Spectrometer by Thermo-Scientific, with an Al K_α_ radiation source (1486.7 eV). During the analyses, the pressure of the analysis chamber was maintained at 5·10^−10^ mbar. The binding energy and kinetic energy scales were calibrated, setting the C 1s transition at 284.6 eV, and the XPS profiles were deconvoluted using the Thermo Avantage software (v5.9929). For the analyses, La 3d^5/2^, Ni 3p, Co 2p, Fe 2p, Mn 2p, Mn 3p and O 1s transitions were studied.
- The reducibility of the samples was explored by developing H_2_-TPR tests that were carried out in a ChemBET *Pulsar* TPR/TPD device from Quantachrome Instruments (Anton Paar Austria GmbH, Graz, Austria) equipped with a Thermal Conductivity Detector (TCD). In a typical experiment, 30 mg of the sample was heated at 10 °C min^−1^ from 25 °C to 1000 °C under a 5% H_2_/Ar atmosphere (40 mL min^−1^). The amount of H_2_ consumed during the H_2_-TPR tests was determined using copper(II) oxide (CuO, Sigma-Aldrich, 99.9 wt%) as a reference sample, which was reduced according to Reaction (5) [133], and the deconvolution of the H_2_-TPR profiles was performed using OriginPro 2016 b9.3.226 software, applying a Lorentzian peak function and a Levenberg–Marquardt iteration algorithm [134].

(5) $$CuO+H_{2}\to Cu+H_{2}O$$

### 3.2. Activity Tests

To estimate the catalytic activity for the DRM reaction, isothermal experiments at 700 °C over 5 h were performed using the following gaseous mixtures as the reactants’ feed: (i) 25% CH_4_ and 25% CO_2_, with 50% He as a balance gas to test the samples under concentrated reactant streams, using a CH_4_/CO_2_ ratio equal to unity (to ensure the most favorable conditions); (ii) 65% CH_4_ and 35% CO_2_ without a balance gas, as an approximation of the average composition of a real biogas [135,136]. Additionally, for the ODRM experiments, different O_2_ percentages (under 1%) were added to both CH_4_ + CO_2_ mixtures. The composition of the inlet gaseous stream was checked before each catalytic experiment using an Agilent ADM1000 flowmeter. To perform these tests, two conditions were used: (i) for the experiments developed under a low-GHSV regime (2980 h^−1^), 50 mg of sample and 100 mg of SiC (used as a diluent to minimize temperature gradients and hotspots [137]) were loaded into a U-shaped quartz reactor, which was subjected to a 60 mL min^−1^ flow of the gas mixture, and (ii) for the experiments under a high-GHSV regime (33,708 h^−1^), a 100 mL min^−1^ flow of the reactant stream and 50 mg of sample (in the absence of SiC) were loaded into a tubular vertical quartz reactor. The GHSV values for both experimental systems were calculated using Equations (6) and (7) [138]:

(6) $$GHSV\left(h^{-1}\right)=\frac{F}{V_{C}}$$

(7) $$V_{C}=\pi r^{2}h$$

where *F* is the volumetric flowrate expressed in mL min^−1^, and *V_C_* is the volume of the catalytic bed, expressed in mL and calculated assuming a cylindrical geometry. Note that low- and high-GHSV experiments were conducted under different reactor configurations (and with and without SiC dilution), so GHSV is only used as label for identifying each configuration.

Prior to the DRM/ODRM tests, the samples were reduced in situ for 2 h at 500 °C, under a 5% H_2_/He gas mixture (40 and 100 mL min^−1^ for GHSV values of 1987 and 33,708 h^−1^, respectively), to obtain the active phase. Additionally, for selected samples, a regeneration step with pure CO_2_ was performed at 700 °C for 1.5 h (GHSV = 2980 h^−1^). The composition of the outlet gas stream was determined using a gas chromatograph (Agilent 8860 or 7890A) equipped with a TCD and two packed columns (Porapak-Q and MolSieve-13X).

The CH_4_ and CO_2_ conversions (Χ_CH4_ and Χ_CO2_) were calculated using Equations (8) and (9):

(8) $$X_{{CH}_{4}}=\frac{{\bar{F}}_{CH_{4},in}-{\bar{F}}_{CH_{4},out}}{{\bar{F}}_{CH_{4},in}}\cdot 100$$

(9) $$X_{{CO}_{2}}=\frac{{\bar{F}}_{CO_{2},in}-{\bar{F}}_{CO_{2},out}}{{\bar{F}}_{CO_{2},in}}\cdot 100$$

with ${\bar{F}}_{CH_{4},in}$/${\bar{F}}_{CO_{2},in}$ and ${\bar{F}}_{CH_{4},out}$/${\bar{F}}_{CO_{2},out}$ being the inlet and outlet molar flowrates of CH_4_/CO_2_, respectively.

The H_2_ yield (*Y_H_*_2_), the CH_4_-specific activity (*a_CH_*_4_), the H_2_/CO ratio and the amount of carbonaceous material deposited (C.D) were obtained based on (10) to (13) [139,140]:

(10) $$Y_{H_{2}}=\frac{{\bar{F}}_{H_{2},out}}{2{\bar{F}}_{CH_{4},in}}\cdot 100$$

(11) $$a_{CH_{4}}=\frac{{\bar{F}}_{CH_{4},in}-{\bar{F}}_{CH_{4},out}}{n_{Ni}+n_{M}}\cdot 100$$

(12) $$H_{2}/CO=\frac{{\bar{F}}_{H_{2},out}}{{\bar{F}}_{CO,out}}$$

(13) $$C.D=\frac{m_{re}-m_{rb}}{m_{s}}$$

with ${\bar{F}}_{H_{2},out}$ and ${\bar{F}}_{CO,out}$ being the outlet molar flowrates of H_2_ and CO, respectively, *n_Ni_* being the number of moles of Ni (active phase for CH_4_ activation [141,142]), *n_M_* being the number of moles of Co or Fe, *m_rb_* and *m_re_* being the mass of the reactor (containing the sample–SiC mixture) at the beginning and end of the reaction, respectively, and *m_s_* being the mass of the sample. Note that, for the calculation of *n_Ni_* and *n_M_*, the weight percentages obtained by μ-XRF were employed. Additionally, the amount of carbon deposited was calculated, for comparison purposes (see the results in Table S5), through the evaluation of the area under the molar carbon balance [143], according to Equation (14):

(14) $$C.D=\frac{1000\;Mm_{C}}{m_{s}}\int_{t_{i}}^{t_{f}}\left({\bar{F}}_{CH_{4},in}+{\bar{F}}_{CO_{2},in}\right)-({\bar{F}}_{CH_{4},out}+{\bar{F}}_{CO_{2},out}+{\bar{F}}_{CO,out})dt$$

where *Mm_C_* is the molar mass of carbon (12 g mol^−1^), and *t_i_* and *t_f_* are the initial and final times of the catalytic experiment. Finally, the carbon balance (C.B) was evaluated using (15) [144,145]:

(15) $$C.B=\frac{{\bar{F}}_{{CH}_{4},out}+{\bar{F}}_{{CO}_{2},out}+{\bar{F}}_{CO,out}}{{\bar{F}}_{CH_{4},in}+{\bar{F}}_{{CO}_{2},in}}\cdot 100$$

Thus, according to this expression, the C.B should be lower than 100% if carbonaceous material is accumulated during the reaction. Note that the outlet molar flowrates were corrected considering the volumetric expansion of the gaseous mixture during the reaction, according to Equation (16) [146,147]:

(16) $${\bar{F}}_{i,out}={\bar{F}}_{i,in}+\upsilon_{i}\;\xi$$

where *υ_i_* is the stoichiometric coefficient of *i* species (positive for products and negative for reactants) and *ξ* the extent of the reaction, calculated using Equation (17) [147].

(17) $$\xi =\frac{{\bar{F}}_{lr,in}-(\chi_{lr,out}\times {\bar{F}}_{total,in})}{1+\delta \chi_{lr,out}}$$

where ${\bar{F}}_{lr,in}$ is the molar flowrate of the limiting reactant, $\chi_{lr,out}$ its outlet molar fraction and *δ* the change in gaseous moles considering the pure DRM process (thus, *δ* = 2). The Supplementary Information file includes a section where this assumption is explored further.

## 4. Conclusions

In this paper, the La_x_NiO_3_, La_0.8_Ni_0.9_M_0.1_O_3_ and La_0.8_Ni_1−y_Co_y_O_3_ (*x* = 1, 0.9, 0.8 and 0.7; *y* = 0.1, 0.25 and 0.5; *M* = Co, Fe and Mn) series of samples were synthesized, characterized and used as precursors of the active phases for the DRM process under reactant atmospheres with different compositions, including those corresponding to the average composition of a real biogas. The selected formulation (La_0.8_Ni_0.75_Co_0.25_O_3_) was also subjected to DRM catalytic tests under more realistic conditions, which included the use of a high-GHSV operating regime, the addition of O_2_ to the reactant mixture (ODRM), and the incorporation of a regeneration step with pure CO_2_ after the DRM/ODRM reactions. According to the results that were presented and discussed, the following conclusions can be drawn:

(i) The percentage of La in the perovskites of the La_x_NiO_3_ series determines the average size of the Ni particles obtained after a reducing treatment at 500 °C, with the La_0.8_NiO_3_ formulation showing slightly smaller particles than the other samples. The decrease in the amount of lanthanum does not modify the conversion of the reactants but increases the amount of deposited carbonaceous material during the DRM tests at 700 °C.
(ii) The partial substitution of Ni by Mn, Fe or Co improves the redox properties of the La_0.8_NiO_3_ perovskite, allowing for the stabilization of the Ni (or Ni + M) particles. The La_0.8_Ni_0.75_Co_0.25_O_3_ formulation allows for the removal of the carbonaceous deposits formed during the DRM reaction under an atmosphere with 25% CH_4_ and 25% CO_2_, as it already features improved resistance to sintering of the metallic particles. However, significant accumulation of carbonaceous material was observed under simulated biogas conditions.
(iii) Using a high-GHSV operating regime, as an approximation of the potential use of the La_0.8_Ni_0.75_Co_0.25_O_3_ formulation in a real application, the conversion of CH_4_ and the production of H_2_ decrease, which is likely attributable to the higher impact of the two parallel reactions (methane decomposition and RWGS), and the amount of deposited carbon increases.
(iv) When O_2_ is present in the reaction atmosphere (ODRM) and using a reaction atmosphere that simulates the average composition of a real biogas, the accumulation of carbonaceous material is not totally avoided. However, although these deposits do not provoke a significant deactivation of the La_0.8_Ni_0.75_Co_0.25_O_3_ catalyst, they can be efficiently eliminated during a regeneration step with CO_2_ at 700 °C.

## Figures and Tables

**Table 1:** Characterization data of the fresh LxN samples.

Sample	Goldschmidt Tolerance Factor (*t*)	μ-XRF La Content (wt%)	XPS		
			Ni(III)/Ni(II)	Ni/(La + Ni) (Nominal)	O_L_/(La + Ni) (Nominal)
LN	0.989	49.5 ± 0.1	2.70 ± 0.03	0.64 ± 0.01 (0.50)	1.01 ± 0.01 (1.50)
L0.9N	0.956	46.7 ± 0.1	1.88 ± 0.02	0.52 ± 0.01 (0.53)	0.99 ± 0.01 (1.57)
L0.8N	0.905	44.9 ± 0.3	1.92 ± 0.02	0.53 ± 0.01 (0.56)	0.81 ± 0.01 (1.67)
L0.7N	0.855	41.7 ± 0.2	1.59 ± 0.02	0.70 ± 0.01 (0.59)	0.97 ± 0.01 (1.76)

**Table 2:** H_2_ consumption volumes of the fresh LxN samples.

Sample ^1,2^	V_NiO_ (mL g^−1^)	V_LaNiO3_ (mL g^−1^)	V_La2Ni2O5_ (mL g^−1^)	V_total_ (mL g^−1^)
LN	47.5 ± 0.2	45.7 ± 0.2	102.6 ± 0.2	195.7 ± 0.6
L0.8N	127.0 ± 0.2	106.8 ± 0.2	73.9 ± 0.2	307.6 ± 0.6
L0.7N	21.5 ± 0.6	86.8 ± 0.2	76.9 ± 0.2	185.2 ± 1.0

^1^ V_NiO_, V_LaNiO3_ and V_La2Ni2O5_ nomenclatures correspond to the H_2_ volumes consumed by each indicated phase. The deconvoluted H_2_-TPR profiles are presented in Figure S1. ^2^ The deconvolution of the L0.9N profile cannot be considered reliable due to the high error associated with its more complex profile.

**Table 3:** CH_4_ and CO_2_ conversions (Χ_CH4_, Χ_CO2_), CH_4_-specific activity (a_CH4_), H_2_ yield (Y_H2_), H_2_/CO product ratio, and amount of carbonaceous material deposited (C.D) during the DRM reaction at 700 °C (25:25 CH_4_:CO_2_) for the LN and L0.8N samples.

Sample	Χ_CH4_ (5 h, %)	Χ_CO2_ (5 h, %)	a_CH4_ (5 h) ^1^	Y_H2_ (5 h, %) ^2^	H_2_/CO (5 h)	C.D (mg C g^−1^)
LN	72 ± 2	76 ± 1	1655 ± 47	44 ± 3	0.46 ± 0.01	580 ± 49
L0.8N	74 ± 2	76 ± 1	1504 ± 42	45 ± 3	0.48 ± 0.01	1687 ± 70

^1^ As mmol of CH_4_ converted per mol of Ni and per minute. ^2^ H_2_ produced through the DRM reaction. The thermodynamic value at 700 °C is 82%.

**Table 4:** Average Ni particle size of the reduced and used LN and L0.8N samples.

Sample	D_Ni_ Reduced (nm)	D_Ni_ Used (nm)
LN	9.7 ± 1.6	27.7 ± 1.4
L0.8N	7.4 ± 1.7	25.7 ± 1.8

**Table 5:** Characterization data of the fresh L0.8N and L0.8NM0.1 samples.

Sample	Goldschmidt Tolerance Factor (*t*)	Dopant Content (wt%)		XPS		
		μ-XRF	ICP-OES	Ni(III)/Ni(II)	O_L_/(La + Ni(+M)) (Nominal = 1.67)	M/(La + Ni + M) (Nominal = 0.056)
L0.8N	0.905	-	-	1.92 ± 0.02	0.81 ± 0.01	-
L0.8NCo0.1	0.906	3.1 ± 0.1	3.14 ± 0.24	1.06 ± 0.01	1.37 ± 0.01	0.046 ± 0.001
L0.8NFe0.1	0.906	3.1 ± 0.1	2.90 ± 0.03	0.63 ± 0.01	1.28 ± 0.01	0.074 ± 0.001
L0.8NMn0.1	0.904	2.4 ± 0.1	2.71 ± 0.03	0.41 ± 0.01	1.28 ± 0.01	0.070 ± 0.003

**Table 6:** H_2_ consumption volumes of the fresh L0.8N and L0.8NM0.1 samples.

Sample ^1^	V_NiO_ (mL g^−1^)	V_LaNiO3_ (mL g^−1^)	V_La2Ni2O5_ (mL g^−1^)	V_total_ (mL g^−1^)
L0.8N	127.0 ± 0.2	106.8 ± 0.2	73.9 ± 0.2	307.6 ± 0.6
L0.8NCo0.1	82.3 ± 0.3	34.0 ± 0.8	98.7 ± 0.2	215.0 ± 1.3
L0.8NFe0.1	44.6 ± 0.4	84.8 ± 0.2	116.6 ± 0.2	246.0 ± 0.8
L0.8NMn0.1	46.9 ± 0.4	84.6 ± 0.2	117.3 ± 0.2	248.9 ± 0.8

^1^ V_NiO_, V_LaNiO3_ and V_La2Ni2O5_ correspond to the consumed H_2_ volumes of each indicated phase. The deconvoluted H_2_-TPR profiles are presented in Figure S7.

**Table 7:** CH_4_ and CO_2_ conversions, CH_4_-specific activity (a_CH4_), H_2_/CO product ratio, H_2_ yield and amount of carbonaceous material deposited (C.D) during the DRM reaction at 700 °C (25:25 CH_4_:CO_2_) for the L0.8N and L0.8NM0.1 samples.

Sample	Χ_CH4_ (5 h, %)	Χ_CO2_ (5 h, %)	a_CH4_ (5 h) ^1^	Y_H2_ (5 h, %) ^2^	H_2_/CO (5 h)	C.D (mg C g^−1^)
L0.8N	74 ± 2	76 ± 1	1504 ± 42	45 ± 3	0.48 ± 0.01	1687 ± 70
L0.8NCo0.1	70 ± 2	78 ± 1	1603 ± 47	47 ± 3	0.51 ± 0.01	512 ± 46
L0.8NFe0.1	66 ± 2	75 ± 1	1478 ± 47	43 ± 3	0.49 ± 0.01	400 ± 45
L0.8NMn0.1	66 ± 2	74 ± 1	1397 ± 44	43 ± 3	0.49 ± 0.01	451 ± 43

^1^ As mmol of CH_4_ converted per mol of Ni (+Co/Fe) and per minute. Mn is not included in this calculation since it is not reduced into Mn(0); thus, it does not contribute to CH_4_ activation. ^2^ H_2_ produced through the DRM reaction. The thermodynamic value at 700 °C is 82%.

**Table 8:** Average metallic particle size of the reduced and used L0.8N and L0.8NM0.1 samples.

Sample	D_Ni(+M)_ Reduced (nm)	D_Ni(+M)_ Used (nm)
L0.8N	7.4 ± 1.7	25.7 ± 1.8
L0.8NCo0.1	6.8 ± 0.8	22.1 ± 1.5
L0.8NFe0.1	5.7 ± 0.9	20.3 ± 1.5
L0.8NMn0.1	7.1 ± 0.9	19.7 ± 1.4

**Table 9:** Characterization data of the fresh L0.8N and L0.8NCox samples.

Sample	Goldschmidt Tolerance Factor (*t*)	Dopant Content (wt%)		XPS		
		μ-XRF	ICP-OES	Ni(III)/Ni(II)	O_L_/(La + Ni(+Co)) (Nominal = 1.67)	Co/(La + Ni + Co) (Nominal)
L0.8N	0.905	-	-	1.92 ± 0.02	0.81 ± 0.01	-
L0.8NCo0.1	0.906	3.1 ± 0.1	3.14 ± 0.24	1.06 ± 0.01	1.37 ± 0.01	0.046 ± 0.001 (0.056)
L0.8NCo0.25	0.907	7.9 ± 0.1	7.81 ± 0.56	3.02 ± 0.04	1.54 ± 0.03	0.118 ± 0.003 (0.139)
L0.8NCo0.5	0.909	16.0 ± 0.3	14.40 ± 0.55	0.93 ± 0.02	1.36 ± 0.03	0.278 ± 0.003 (0.278)

**Table 10:** H_2_ consumption volumes for the fresh L0.8N and L0.8NM0.1 samples.

Sample ^1^	V_NiO_ (mL g^−1^)	V_LaNiO3_ (mL g^−1^)	V_La2Ni2O5_ (mL g^−1^)	V_total_ (mL g^−1^)
L0.8N	127.0 ± 0.2	106.8 ± 0.2	73.9 ± 0.2	307.6 ± 0.6
L0.8NCo0.1	82.3 ± 0.3	34.0 ± 0.8	98.7 ± 0.2	215.0 ± 1.3
L0.8NCo0.25	42.3 ± 0.7	68.6 ± 0.4	142.2 ± 0.2	253.1 ± 1.3
L0.8NCo0.5	41.5 ± 0.7	48.8 ± 0.5	117.9 ± 0.2	208.2 ± 1.4

^1^ V_NiO_, V_LaNiO3_ and V_La2Ni2O5_ correspond to the volume of consumed H_2_ in each indicated phase. The deconvoluted H_2_-TPR profiles are presented in Figure S10.

**Table 11:** CH_4_ and CO_2_ conversions, CH_4_-specific activity (a_CH4_), H_2_/CO product ratio, H_2_ yield and amount of carbonaceous material deposited (C.D) during the DRM reaction at 700 °C (25:25 CH_4_:CO_2_) for the L0.8N and L0.8NCox samples.

Sample	Χ_CH4_ (5 h, %)	Χ_CO2_ (5 h, %)	a_CH4_ (5 h) ^1^	Y_H2_ (5 h, %) ^2^	H_2_/CO (5 h)	C.D (mg C g^−1^) ^3^
L0.8N	74 ± 2	76 ± 1	1504 ± 42	45 ± 3	0.48 ± 0.01	1687 ± 70
L0.8NCo0.1	70 ± 2	78 ± 1	1603 ± 47	47 ± 3	0.51 ± 0.01	512 ± 46
L0.8NCo0.25	57 ± 2	70 ± 1	1362 ± 50	37 ± 3	0.50 ± 0.01	<LOD
L0.8NCo0.5	50 ± 2	64 ± 1	1397 ± 48	33 ± 3	0.48 ± 0.01	<LOD

^1^ As mmol of CH_4_ converted per mol of Ni (+Co) and per minute. ^2^ H_2_ produced through the DRM reaction. The thermodynamic value at 700 °C is 82%. ^3^ “LOD” refers to the limit of detection.

**Table 12:** Average metallic particle size of the reduced and used L0.8N and L0.8NCox samples.

Sample	D_Ni(+Co)_ Reduced (nm)	D_Ni(+Co)_ Used (nm)
L0.8N	7.4 ± 1.7	25.7 ± 1.8
L0.8NCo0.1	6.8 ± 0.8	22.1 ± 1.5
L0.8NCo0.25	6.6 ± 0.6	8.9 ± 0.5
L0.8NCo0.5	7.1 ± 0.7	9.5 ± 0.8

**Table 13:** CH_4_ and CO_2_ conversions, CH_4_-specific activity (a_CH4_), H_2_/CO product ratio, H_2_ yield and the amount of carbonaceous material deposited (C.D) during the DRM reaction at 700 °C (65:35 CH_4_:CO_2_) for the L0.8N and L0.8NCo0.25 samples.

Sample	Χ_CH4_ (5 h, %)	Χ_CO2_ (5 h, %)	a_CH4_ (5 h) ^1^	Y_H2_ (5 h, %) ^2^	H_2_/CO (5 h)	C.D (mg C g^−1^)
L0.8N	35 ± 2	59 ± 1	1893 ± 110	20 ± 1	0.47 ± 0.01	3584 ± 109
L0.8NCo0.25	43 ± 2	71 ± 1	2241 ± 115	19 ± 1	0.48 ± 0.01	3616 ± 108

^1^ As mmol of CH_4_ converted per mol of Ni (+ Co) and per minute. ^2^ H_2_ produced through the DRM reaction. The thermodynamic value at 700 °C is higher than 82%.

**Table 14:** CH_4_ and CO_2_ conversions, CH_4_-specific activity (a_CH4_), H_2_/CO product ratio, H_2_ yield and amount of carbonaceous material deposited (C.D) during the DRM reaction at 700 °C (25:25 CH_4_:CO_2_) under different operating regimes (low-GHSV (2980 h^−1^) and high-GHSV (33,708 h^−1^)).

GHSV (h^−1^)	Χ_CH4_ (ft, %) ^1^	Χ_CO2_ (ft, %) ^1^	a_CH4_ (ft) ^1,2^	Y_H2_ (ft, %) ^1,3^	H_2_/CO (ft) ^1^	C.D (mg C g^−1^) ^4^
2980	57 ± 2	70 ± 1	1362 ± 50	37 ± 3	0.50 ± 0.01	<LOD
33,708	52 ± 4	83 ± 4	1655 ± 128	15 ± 1	0.50 ± 0.01	3302 ± 90

^1^ The term “ft” refers to the final time of the experiment. ^2^ As mmol of CH_4_ converted per mol of Ni + Co and per minute. ^3^ H_2_ produced through the DRM reaction. The thermodynamic value at 700 °C is 82%. ^4^ “LOD” refers to the limit of detection.

**Table 15:** CH_4_ and CO_2_ conversions, CH_4_-specific activity (a_CH4_), H_2_/CO product ratio, H_2_ yield and amount of carbonaceous material deposited (C.D) during the ODRM reaction at 700 °C under the 25:25 atmosphere using different O_2_ percentages (GHSV = 33,708 h^−1^).

O_2_ (%)	Χ_CH4_ (ft, %) ^1^	Χ_CO2_ (ft, %) ^1^	a_CH4_ (ft) ^1,2^	Y_H2_ (ft, %) ^1^	H_2_/CO (ft) ^1^	C.D (mg C g^−1^)
0	52 ± 4	83 ± 4	1655 ± 128	15 ± 1	0.50 ± 0.01	3302 ± 90
0.5	46 ± 4	82 ± 4	1653 ± 81	14 ± 1	0.43 ± 0.01	1350 ± 54
0.9	40 ± 4	75 ± 4	1406 ± 75	13 ± 1	0.43 ± 0.01	1520 ± 65

^1^ The term “ft” refers to the final time of the experiment. ^2^ As mmol of CH_4_ converted per mol of Ni + Co and per minute.

**Table 16:** CH_4_ and CO_2_ conversions, CH_4_-specific activity (a_CH4_), H_2_/CO product ratio, H_2_ yield and amount of carbonaceous material deposited (C.D) during the ODRM reaction at 700 °C under the simulated biogas atmosphere using different O_2_ percentages (GHSV = 33,708 h^−1^).

O_2_ (%) ^1^	Χ_CH4_ (5 h, %)	Χ_CO2_ (5 h, %)	a_CH4_ (5 h) ^2^	Y_H2_ (5 h, %)	H_2_/CO (5 h)	C.D (mg C g^−1^)
0.5	34 ± 4	86 ± 4	2918 ± 136	7 ± 1	0.37 ± 0.01	2117 ± 81
0.9	35 ± 4	86 ± 4	2740 ± 128	7 ± 1	0.38 ± 0.01	1561 ± 70

^1^ The DRM test in the absence of O_2_ and in the high-GHSV configuration was not carried out for safety reasons since, under the 25:25 atmosphere, the high amount of carbonaceous deposits collapsed the reactor. ^2^ As mmol of CH_4_ converted per mol of Ni + Co and per minute.

**Table 17:** CH_4_ and CO_2_ conversions, CH_4_-specific activity (a_CH4_), H_2_/CO product ratio, H_2_ yield and amount of carbonaceous material deposited (C.D) during two ODRM cycles at 700 °C under the simulated biogas atmosphere (0.5% O_2_ and GHSV = 2980 h^−1^).

Cycle ^1^	Χ_CH4_ (5 h, %)	Χ_CO2_ (5 h, %)	a_CH4_ (5 h) ^2^	Y_H2_ (5 h, %)	H_2_/CO (5 h)	C.D (mg C g^−1^)
1	41 ± 2	66 ± 1	1746 ± 87	18 ± 1	0.53 ± 0.01	1821 ± 68
2	35 ± 2	59 ± 1	1487 ± 89	15 ± 1	0.51 ± 0.01	345 ± 42

^1^ The obtained catalytic data during the first cycle is very similar (but not identical) to the presented results in Table 16 for the same operating regime, as the data in this table corresponds to a different experiment carried out under the same conditions. ^2^ As mmol of CH_4_ converted per mol of Ni + Co and per minute.

## Data Availability

The raw data supporting the conclusions of this article will be made available by the authors on request.

## Supplementary Materials

The following supporting information can be downloaded at: https://www.mdpi.com/article/10.3390/molecules31183284/s1, Figure S1: Deconvoluted H_2_-TPR profiles of the LxN series; Table S1: CH_4_ and CO_2_ conversions, CH_4_-specific activity (a_CH4_), H_2_/CO product ratio, H_2_ yield and amount of carbonaceous material deposited (C.D) during the DRM reaction at 700 °C (25:25 CH_4_:CO_2_) for the LN and L0.8N samples reduced at different temperatures; Figure S2: CH_4_ (a) and CO_2_ (b) conversions, H_2_/CO ratio (c) and carbon balance (d) during the DRM reaction at 700 °C (25:25 CH_4_:CO_2_) for the LN and L0.8N samples reduced at different temperatures; Figure S3: XRD patterns and average Ni particle size (D_Ni_) of the LN sample reduced at three different temperatures; Table S2: Average particle sizes of the LN sample reduced at different temperatures, measured after being used in the catalytic tests; Figure S4: FE-SEM images of the reduced LN sample at 500 °C; Figure S5: CH_4_ (a) and CO_2_ (b) conversions, H_2_/CO ratio (c) and carbon balance (d) during the DRM reaction at 700 °C (25:25 CH_4_:CO_2_) for the LxN series; Table S3: CH_4_ and CO_2_ conversions, CH_4_-specific activity (a_CH4_), H_2_/CO product ratio, H_2_ yield and amount of carbonaceous material deposited (C.D) during the DRM reaction at 700 °C (25:25 CH_4_:CO_2_) for the LxN series; Figure S6: TEM images of the LN (below) and L0.8N (above) samples used after the DRM tests at 700 °C under the 25:25 atmosphere; Figure S7: Deconvoluted H_2_-TPR profiles of the L0.8NM0.1 series; Figure S8: TEM images of the reduced L0.8NCo0.1 (left), L0.8NFe0.1 (right) and L0.8NMn0.1 (below) samples; Figure S9: TEM images of the L0.8NCo0.1 (left), L0.8NFe0.1 (right) and L0.8NMn0.1 (below) samples after the DRM tests at 700 °C under the 25:25 atmosphere; Figure S10: Deconvoluted H_2_-TPR profiles of the L0.8NCox series; Figure S11: TEM images of the reduced L0.8NCo0.25 (left) and L0.8NCo0.5 (right) samples; Figure S12: TEM images of the L0.8NCo0.25 (left) and L0.8NCo0.5 (right) samples after the DRM tests at 700 °C under the 25:25 atmosphere; Figure S13: TEM image of the L0.8NCo0.25 sample after the DRM test at 700 °C under the simulated biogas atmosphere; Figure S14: CH_4_ (a) and CO_2_ (b) conversions, H_2_/CO ratio (c) and carbon balance (d) for the ODRM reaction at 700 °C under a simulated biogas reactant atmosphere, with 0.5% O_2_, and under both operating regimes; Table S4: CH_4_ and CO_2_ conversions, CH_4_-specific activity (a_CH4_), H_2_/CO product ratio, H_2_ yield and amount of carbonaceous material deposited (C.D) during the ODRM reaction at 700 °C under a simulated biogas atmosphere, with 0.5% O_2_, and under both operating regimes; Figure S15: TEM images of the L0.8NCo0.25 sample after the first (left) and second (right) ODRM cycle at 700 °C under the simulated biogas atmosphere; Table S5: Difference between the reactant conversions at 5 h and at 30 min (ΔΧ_CH4_/ΔΧ_CO2_) observed in the different catalytic tests for all the samples, and the amount of carbonaceous material deposited, calculated using the molar carbon balance; Figure S16: Carbon balance profiles for three selected catalysts, calculated using the two δ values (2 and 1.33).

## Abbreviations

The following abbreviations are used in this manuscript:

GHGs	Greenhouse Gases
DRM	Dry Reforming of Methane
RWGS	Reverse Water Gas Shift
ODRM	Oxidative Dry Reforming of Methane
XRD	X-Ray Diffraction
JCPDS	Joint Committee on Powder Diffraction Standards
ICDD	International Centre for Diffraction Data
μ-XRF	X-Ray Microfluorescence
XPS	X-ray Photoelectron Spectroscopy
H_2_-TPR	Temperature-Programmed Reduction with H_2_
FE-SEM	Field Emission Scanning Electron Microscopy
TEM	Transmission Electron Microscopy
ICP-OES	Inductively Coupled Plasma Optical Emission Spectroscopy
GHSV	Gas Hourly Space Velocity
TCD	Thermal Conductivity Detector
LOD	Limit of Detection
