# Supplementary material for: Optimizing LaNiO3 Perovskite as Catalyst Precursor for the Revalorization of Biogas by Dry Reforming of Methane
Source: Molecules. 2026 Sep 16;31(18):3284. doi: 10.3390/molecules31183284 (PMC13610200; doi:10.3390/molecules31183284)
Supplement: Supplementary file 1 [file molecules-31-03284-s001.zip › molecules-4484778-supplementary.pdf]

Optimizing $LaNiO_3$ perovskite as catalyst precursor for the revalorization of biogas by Dry Reforming of Methane

Álvaro Díaz-Verde[a], Jonathan Cavazzani[b], Antonella Glisenti[b,c], Maria José Illán-Gómez[a,*]

[a] *Carbon Materials and Environment Research Group. Inorganic Chemistry Department. Materials Institute of the University of Alicante (IUMA). Faculty of Sciences. University of Alicante. Ctra. San Vicente del Raspeig s/n, San Vicente del Raspeig, 03690 Alicante, Spain.*

[b] *Innovative Materials and Processes for Advanced Environmental Clean Technologies Research Group. Department of Chemical Sciences. University of Padova. Via F. Marzolo, 1, 35131. Padova, Italy.*

[c] *National Council Research Institute of Condensed Matter Chemistry and Technology for Energy (CNR ICMATE). Via F. Marzolo, 1, 35131. Padova, Italy.*

Corresponding authors

illan@ua.es *(M. J. Illán-Gómez, +34 965903400 x 3544).*

H_2-TPR analysis of the LxN series

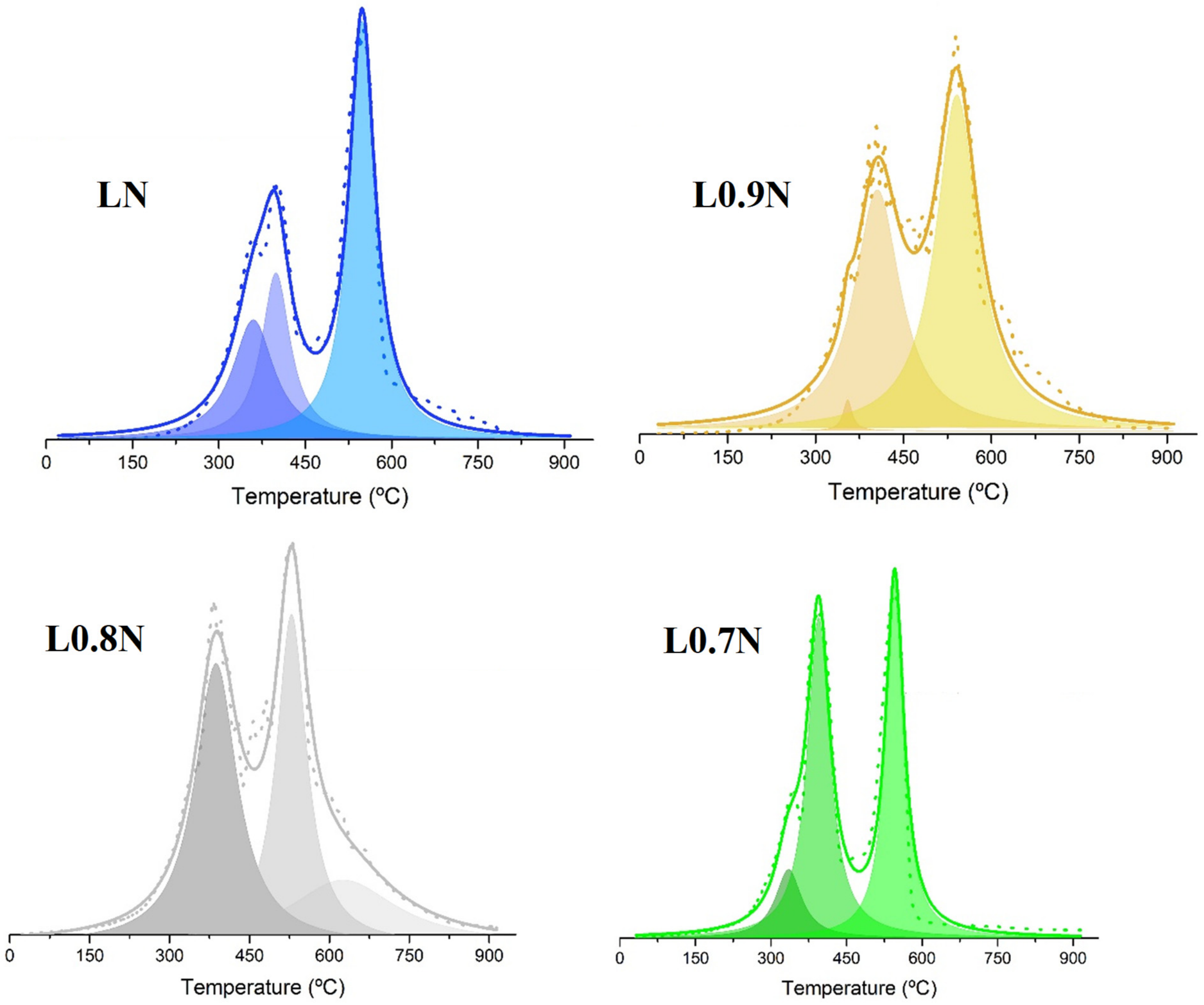

Figure S1. Deconvoluted H_2-TPR profiles of the LxN series.

Selection of the reduction temperature

To select the reduction temperature to be used before the DRM catalytic tests, three temperatures (500ºC, 600ºC and 700ºC) were evaluated using two representative samples: LN, as reference, and L0.8N, selected for its slightly lower average Ni particle size. The reduction temperature was included in the nomenclature of the samples: LxN 500, LxN 600 and LxN 700.

The data displayed in Table S1 and Figure S1 reveal that, overall, increasing the reduction temperature has little effect on reactant conversions, H_2 yields, CH_4 specific activity and H_2/CO ratios. However, the amount of carbonaceous material deposited increases with reduction temperature, suggesting that higher reduction temperatures favor CH_4 decomposition over the DRM pathway.

Table S1. CH_4 and CO_2 conversions, CH_4 specific activity (a_{CH4}), H_2/CO product ratio, H_2 yield and amount of carbonaceous material deposited (C.D) during the DRM reaction at 700ºC (25:25 $CH_4:CO_2$) for the LN and L0.8N samples reduced at different temperatures.

Sample	X_{CH4} (5 h, %)	X_{CO2} (5 h, %)	ΔX_{CH4} / ΔX_{CO2} (%)[1]	a_{CH4} (5 h)[2]	Y_{H2} (5 h, %)[3]	H_2/CO (5 h)	C.D (mg C g^{-1})
**LN 500**	72 ± 2	76 ± 1	12 ± 4 / 9 ± 2	1655 ± 47	44 ± 3	0.46 ± 0.01	580 ± 49
**LN 600**	66 ± 2	77 ± 1	6 ± 4 / 2 ± 2	1551 ± 49	41 ± 3	0.51 ± 0.01	982 ± 59
**LN 700**	68 ± 2	77 ± 1	7 ± 4 / 6 ± 2	1636 ± 50	44 ± 3	0.52 ± 0.01	1004 ± 60
**L0.8N 500**	74 ± 2	76 ± 1	9 ± 4 / 5 ± 2	1504 ± 42	45 ± 3	0.48 ± 0.01	1687 ± 70
**L0.8N 600**	72 ± 2	79 ± 1	3 ± 4 / 2 ± 2	1512 ± 44	47 ± 2	0.54 ± 0.01	2089 ± 81
**L0.8N 700**	74 ± 2	79 ± 1	6 ± 4 / 3 ± 2	1491 ± 42	49 ± 3	0.58 ± 0.01	2599 ± 87

[1] Calculated as the difference between the reactant conversions at 5 h and at 30 min of reaction.

[2] As mmol of CH_4 converted per mol of Ni and per minute.

[3] H_2 produced through the DRM reaction. The thermodynamic value at 700ºC is higher than 82 %.

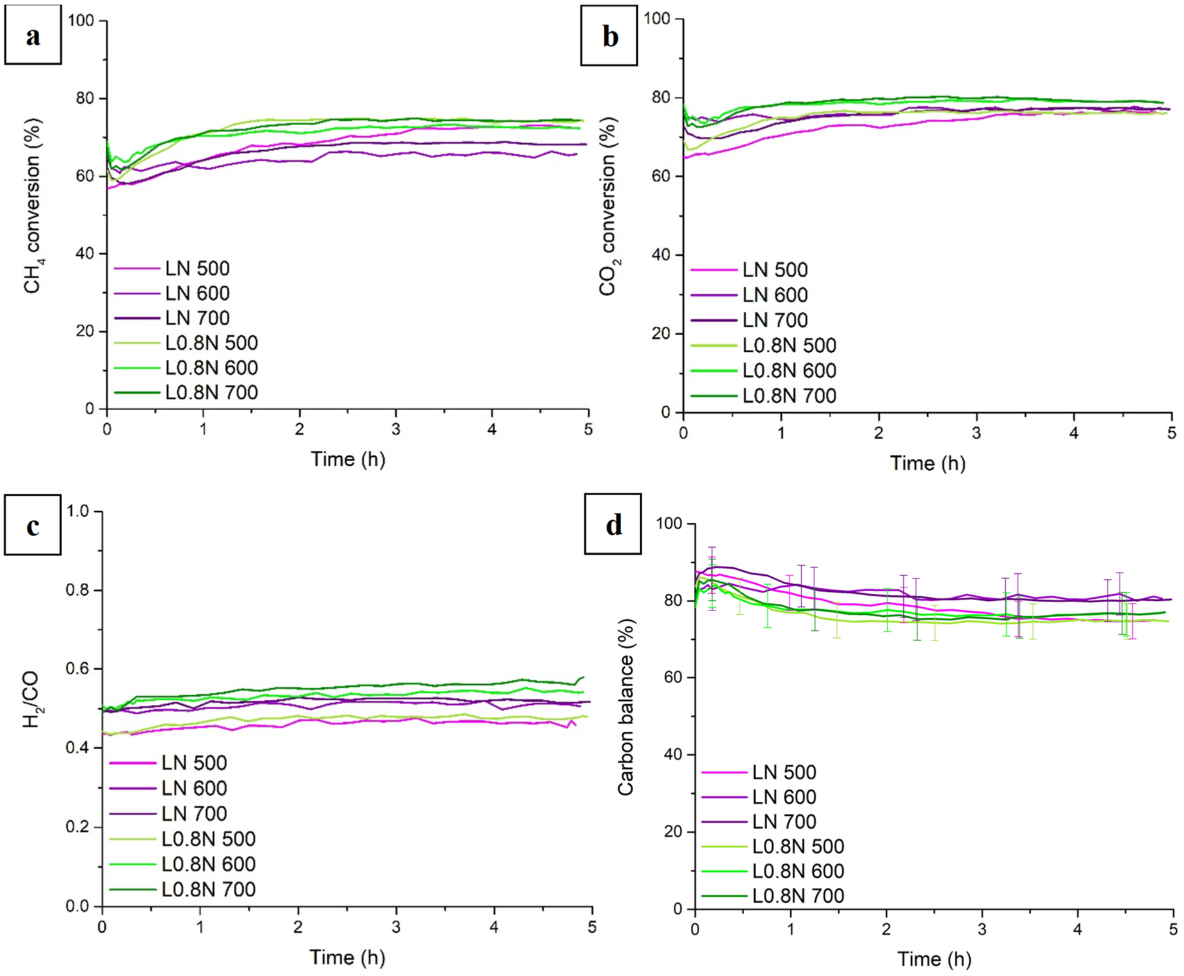

Figure S2. CH_4 (a) and CO_2 (b) conversions, H_2/CO ratio (c) and carbon balance (d) during the DRM reaction at 700ºC (25:25 CH_4:CO_2) for the LN and L0.8N samples reduced at different temperatures.

The XRD profiles of the LN sample reduced at different temperatures, featured in Figure S3, allow to confirm the formation of the Ni/La_2O_3 system after the reduction treatment, since Ni (JCPDS-ICDD 04-0850), La_2O_3 (JCPDS-ICDD 74-1144) and $La(OH)_3$ phases (JCPDS-ICDD 36-1481) were identified. Note that, the average Ni particle size of the reduced sample (included in Figure S3) seems not being significantly affected by the reduction temperature. However, according to the data including in Table S2, higher reduction temperatures lead to slight sintering during the catalytic tests which is not reflected in the amount of carbonaceous material deposited (Table S1). Thus, since reduction temperatures above 500ºC do not improve the catalytic performance (despite yielding more stable Ni particles), 500ºC was selected for developing the reducing step prior to the DRM tests.

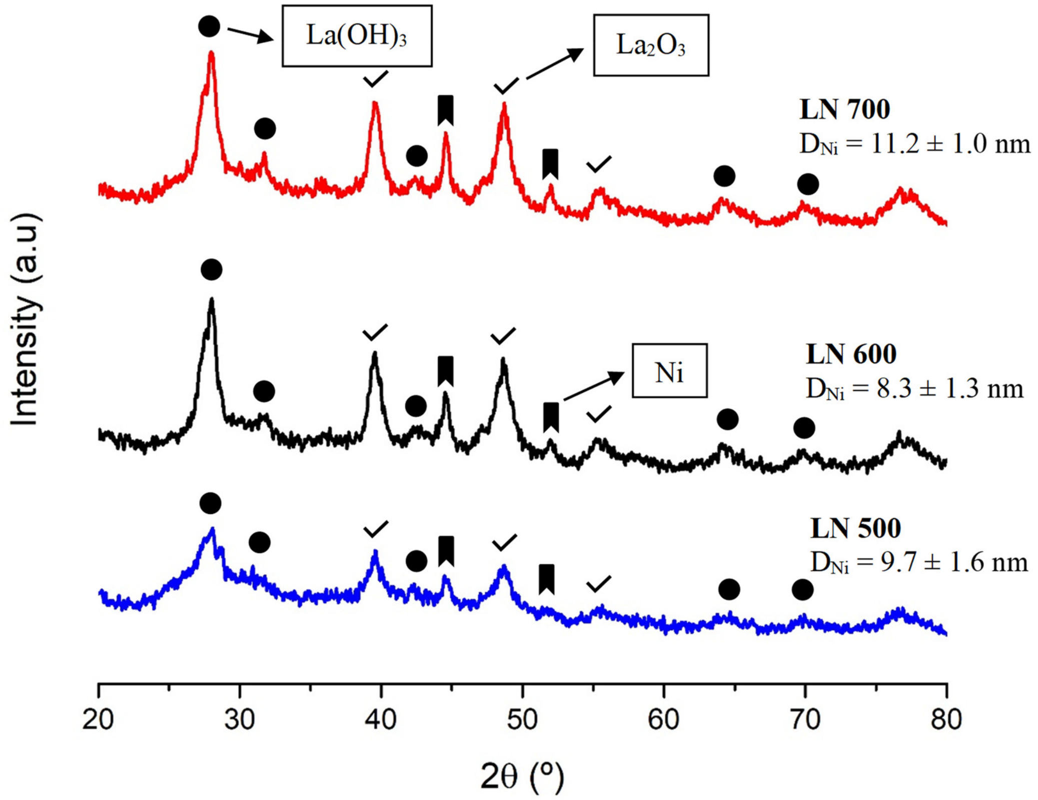

Figure S3. XRD patterns and average Ni particle size (D_{Ni}) of the LN sample reduced at the three different temperatures.

Table S2. Average particle sizes of the reduced LN sample at the different temperatures, measured after being used in the catalytic tests.

**Sample**	**D_{Ni} (nm)**		
	**500ºC**	**600ºC**	**700ºC**
**LN**	27.7 ± 1.4	11.0 ± 0.3	14.2 ± 0.5

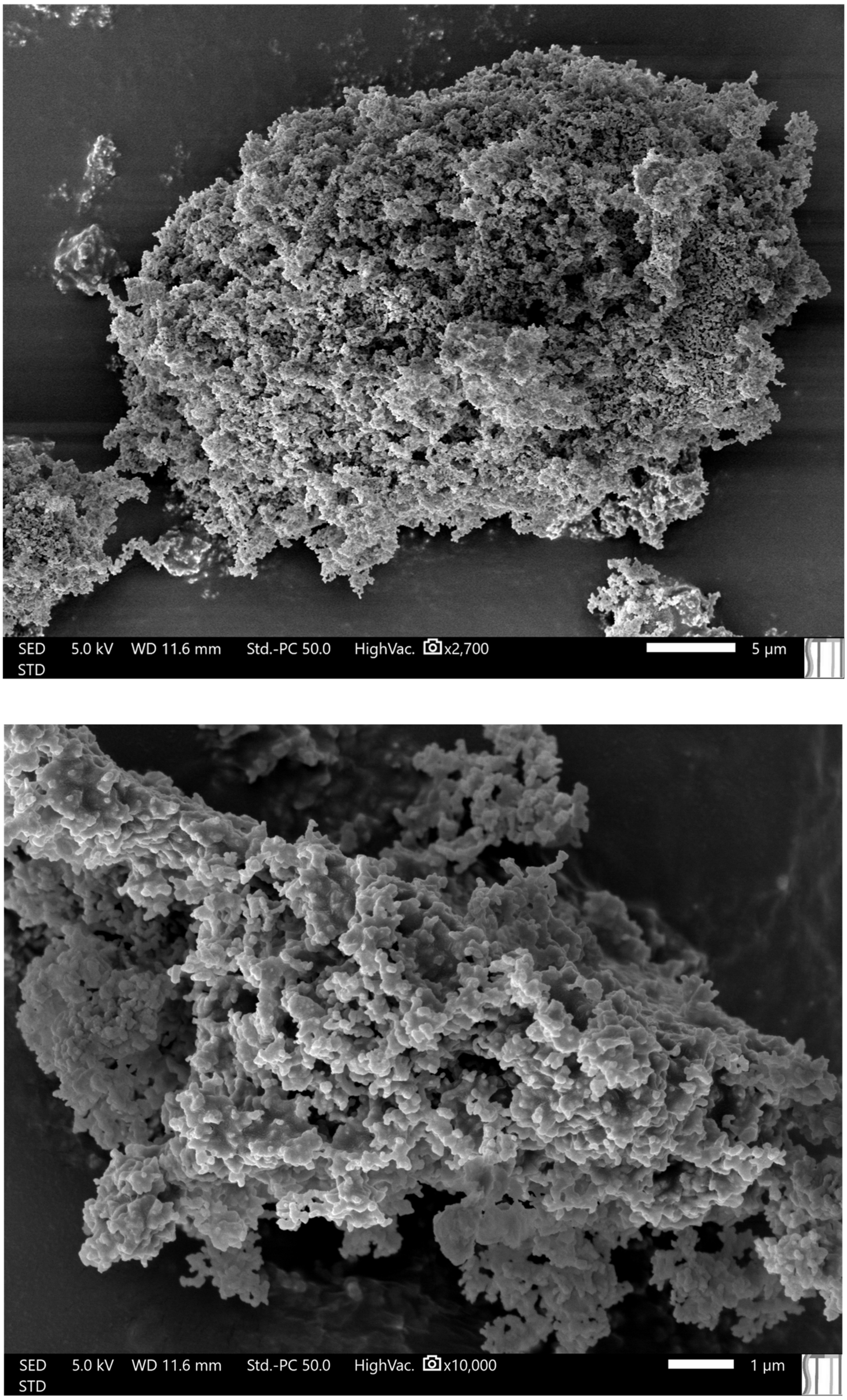

Figure S4. FE-SEM images of the reduced LN sample at 500ºC.

Catalytic tests for the LxN series

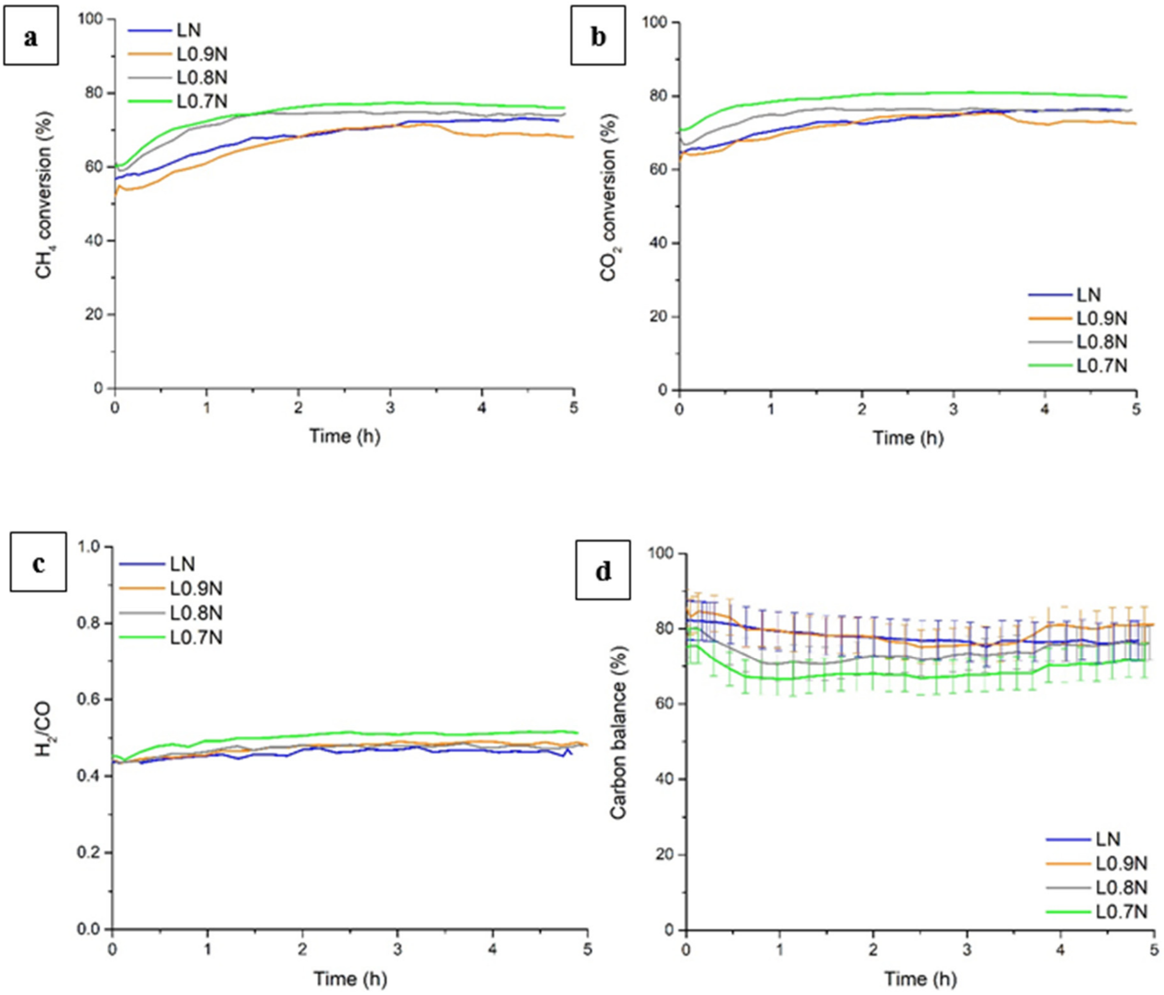

Figure S5. CH_4 (a) and CO_2 (b) conversions, H_2/CO ratio (c) and carbon balance (d) during the DRM reaction at 700ºC (25:25 CH_4:CO_2) for the complete LxN series.

Table S3. CH_4 and CO_2 conversions, CH_4 specific activity (a_{CH4}), H_2/CO product ratio, H_2 yield and amount of carbonaceous material deposited (C.D) during the DRM reaction at 700ºC (25:25 CH_4:CO_2) for the complete LxN series.

Sample	X_{CH4} (5 h, %)	X_{CO2} (5 h, %)	a_{CH4} (5 h)[1]	Y_{H2} (5 h, %)[2]	H_2/CO (5 h)	C.D (mg C g^{-1})
LN	72 ± 2	76 ± 1	1655 ± 47	44 ± 3	0.46 ± 0.01	580 ± 49
L0.9N	68 ± 2	73 ± 1	1456 ± 46	41 ± 3	0.48 ± 0.01	1698 ± 70
L0.8N	74 ± 2	76 ± 1	1504 ± 42	45 ± 3	0.48 ± 0.01	1687 ± 70
L0.7N	76 ± 2	80 ± 1	1514 ± 47	48 ± 3	0.51 ± 0.01	2320 ± 86

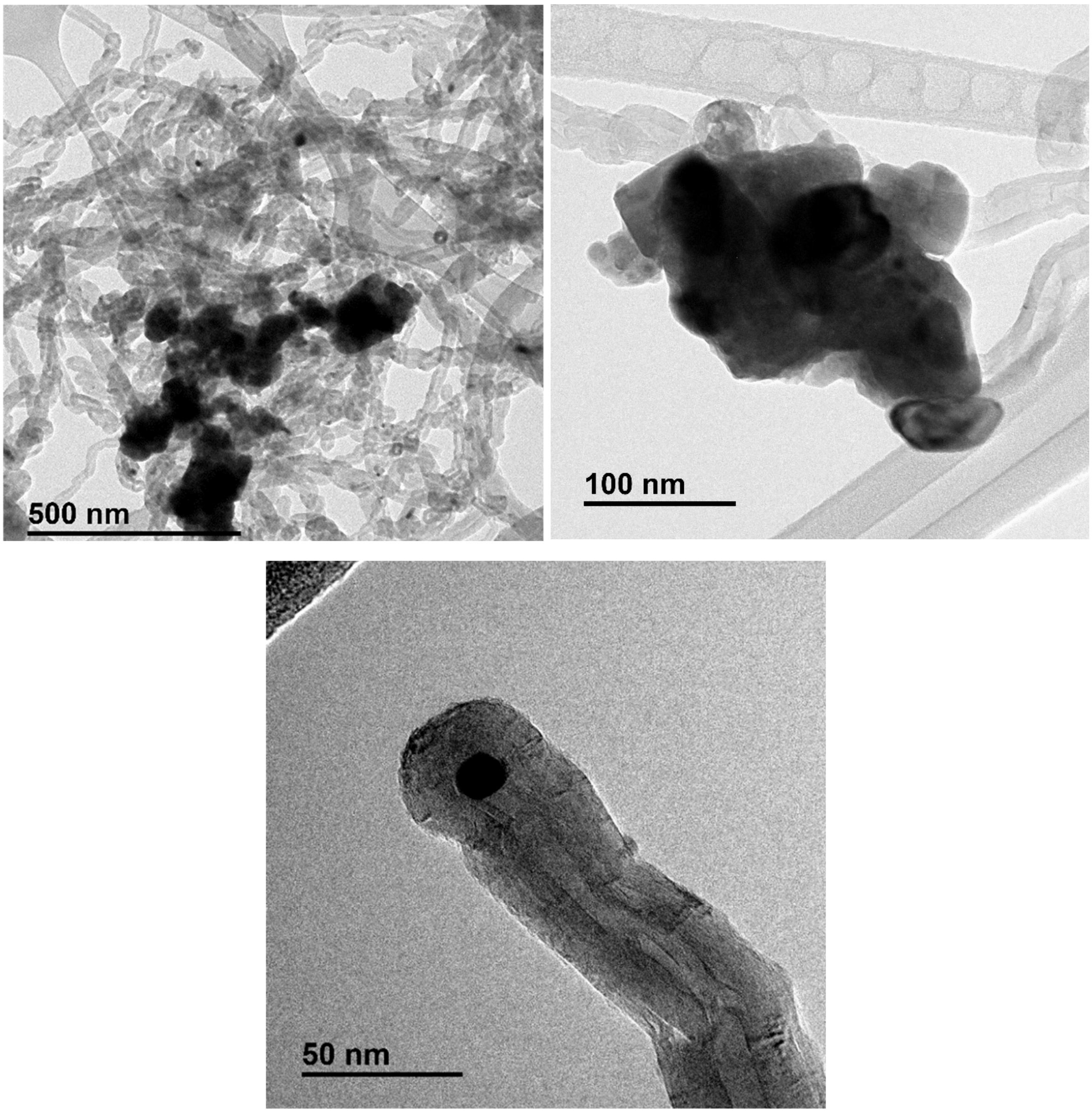

Figure S6. TEM images of the used LN (below) and L0.8N (above) samples after the DRM tests at 700ºC under the 25:25 atmosphere.

H_2-TPR analysis of the L0.8NM0.1 series

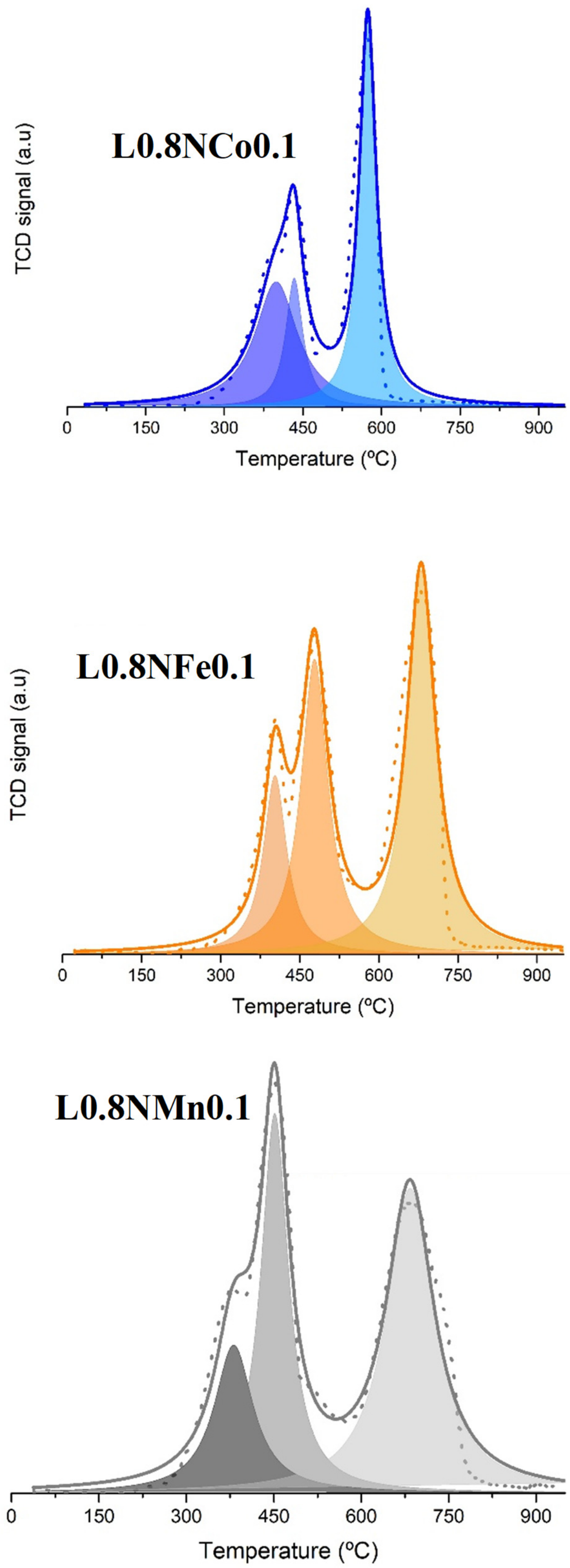

Figure S7. Deconvoluted H_2-TPR profiles of the L0.8NM0.1 series.

TEM analysis of the L0.8NM0.1 samples

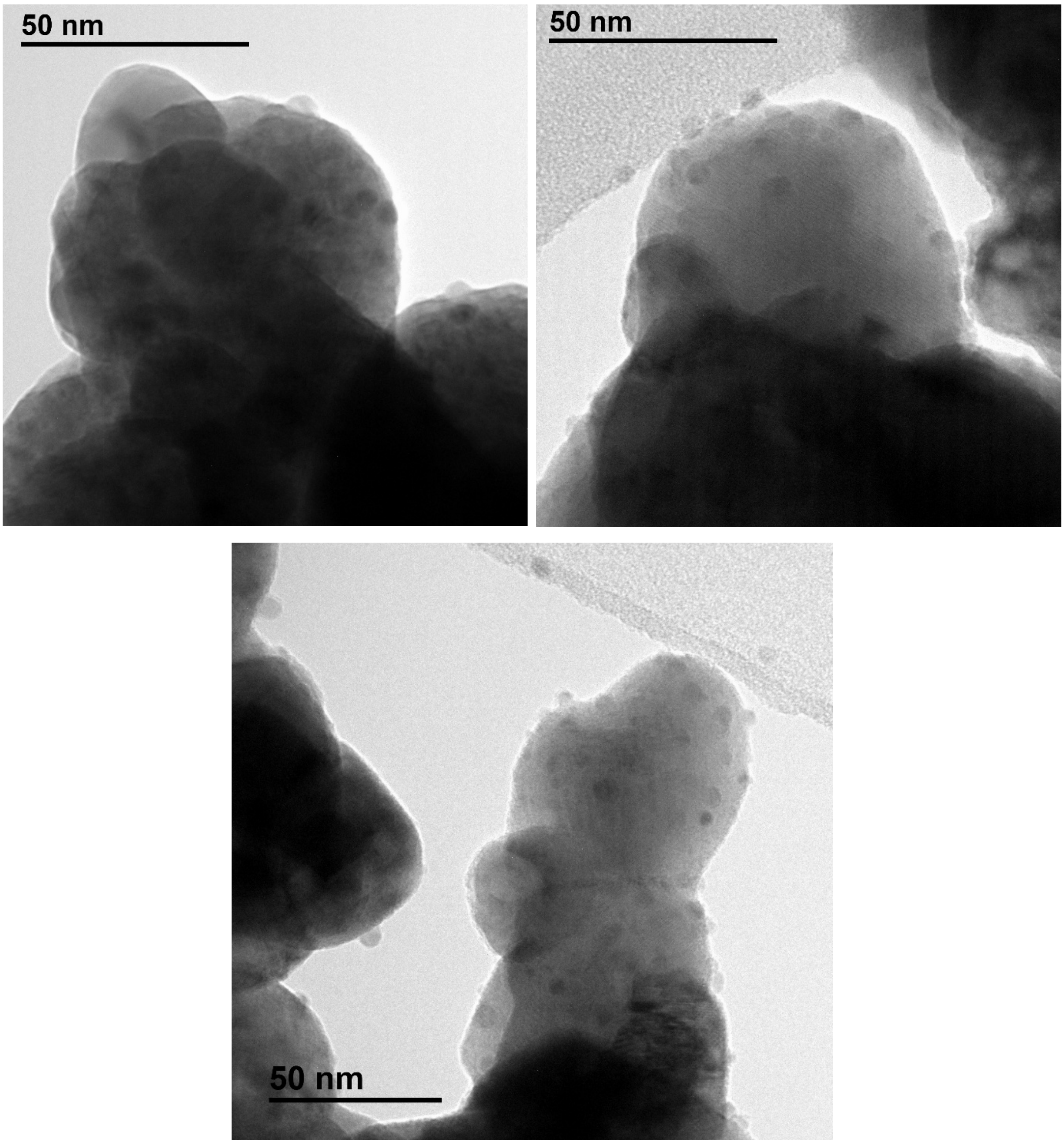

Figure S8. TEM images of the reduced L0.8NCo0.1 (left), L0.8NFe0.1 (right) and L0.8NMn0.1 (below) samples.

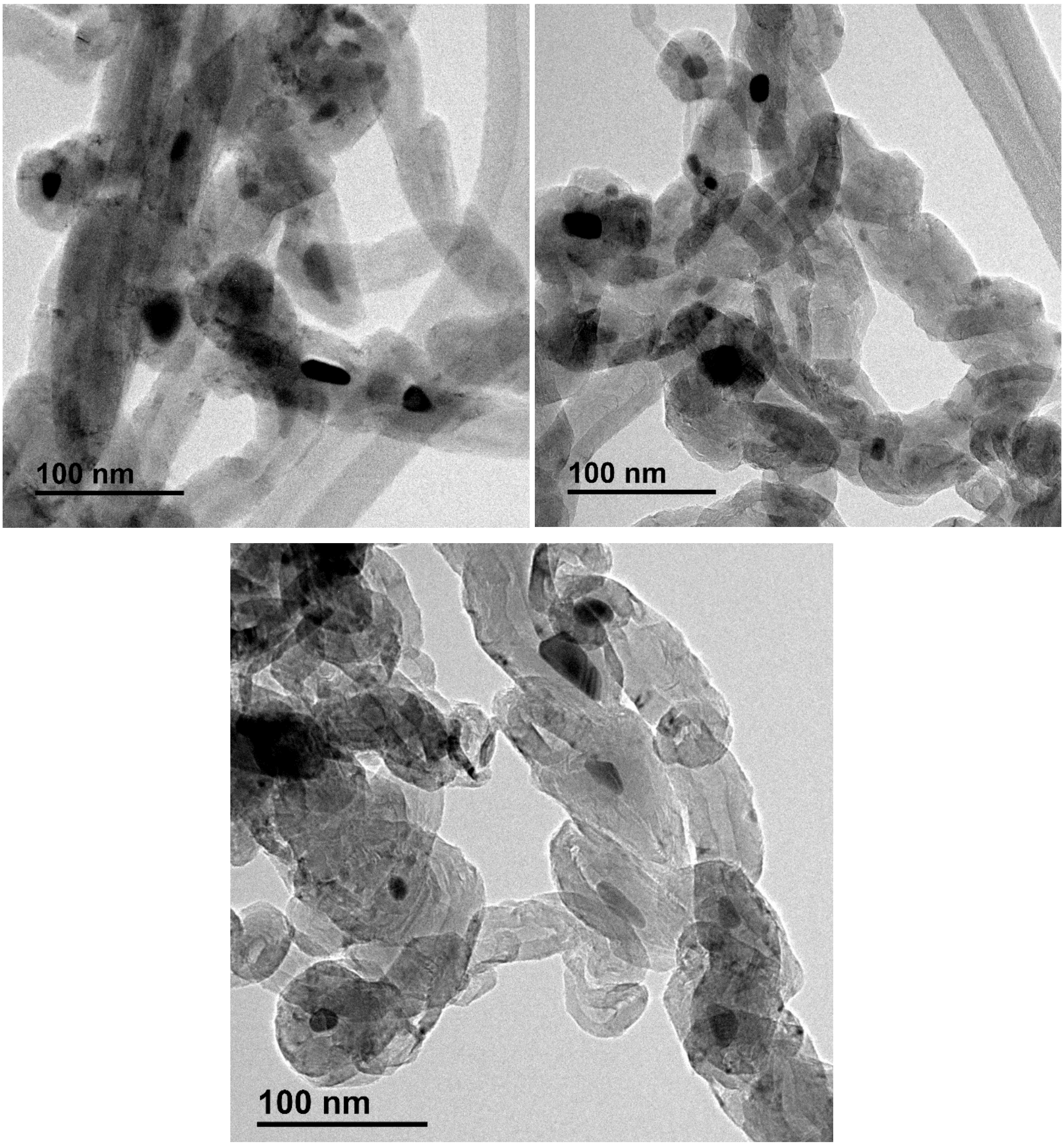

Figure S9. TEM images of the used L0.8NCo0.1 (left), L0.8NFe0.1 (right) and L0.8NMn0.1 (below) samples after the DRM tests at 700ºC under the 25:25 atmosphere.

H_2-TPR analysis of the L0.8NCox series

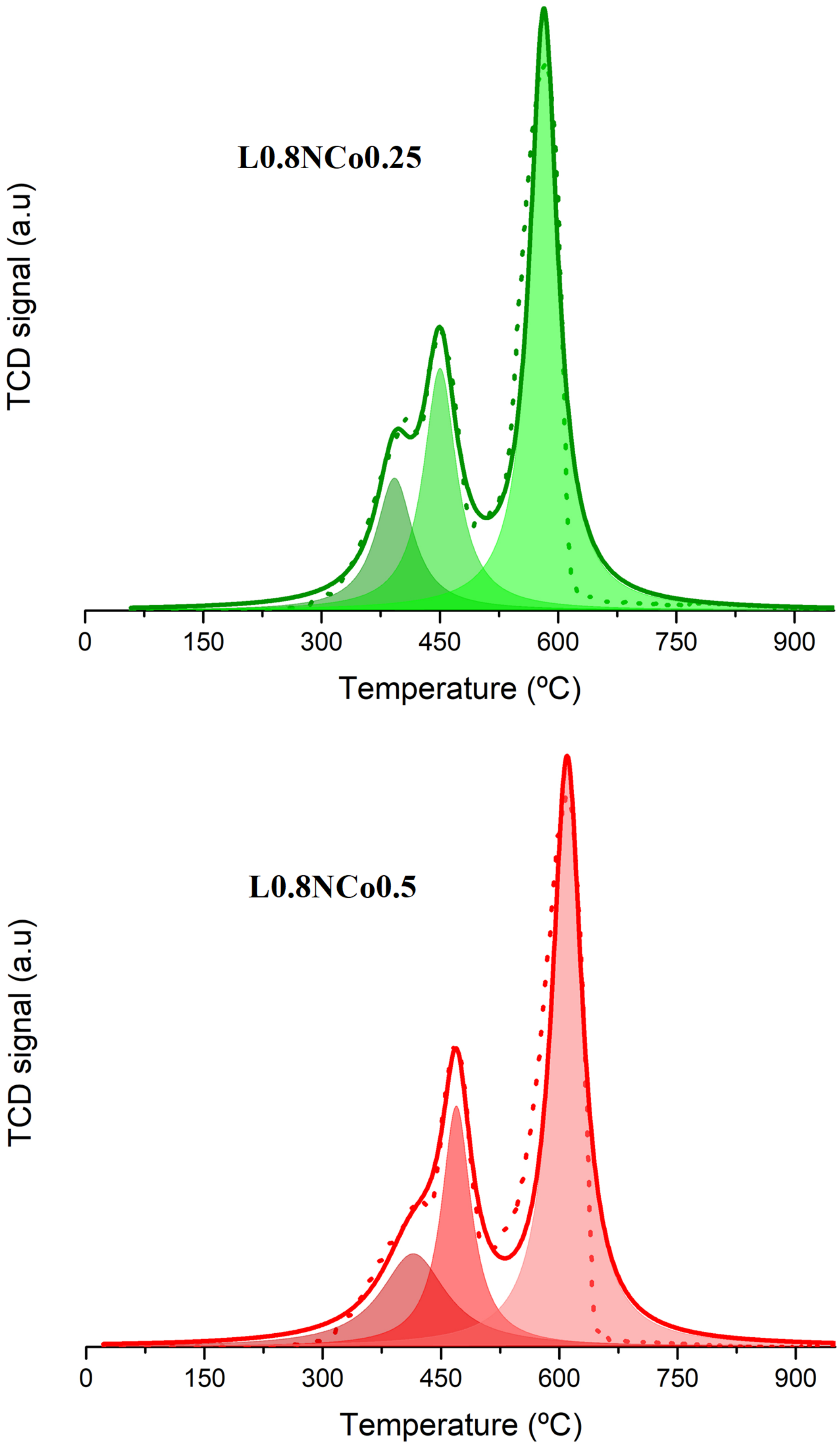

Figure S10. Deconvoluted H_2-TPR profiles of the L0.8NCox series.

TEM analysis of the L0.8NCox samples

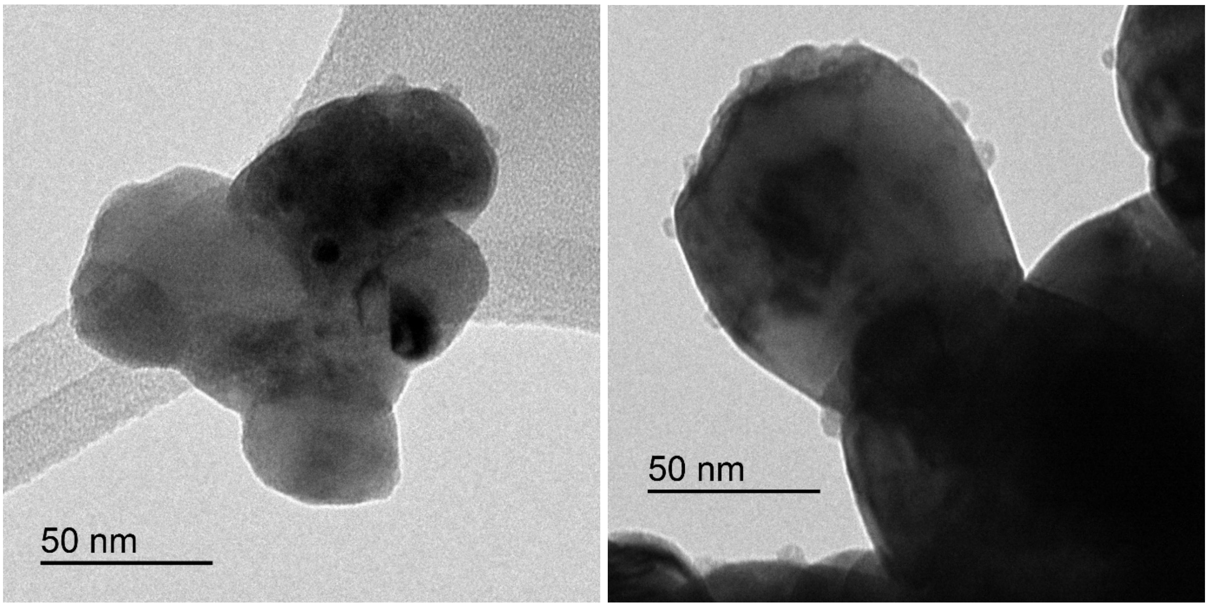

Figure S11. TEM images of the reduced L0.8NCo0.25 (left) and L0.8NCo0.5 (right) samples.

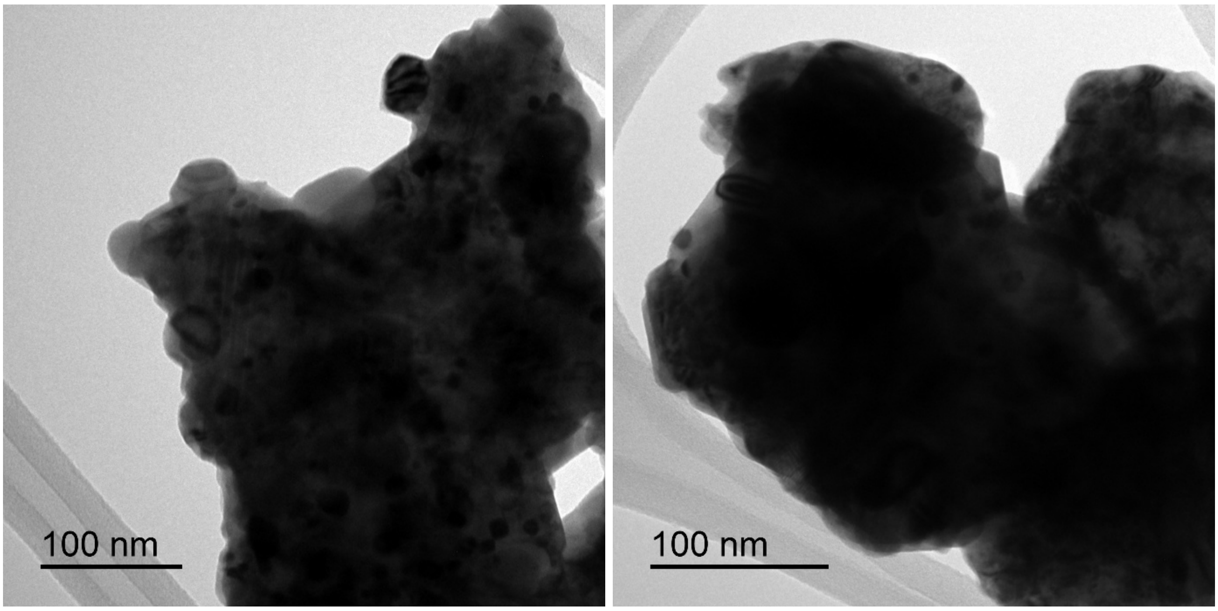

Figure S12. TEM images of the used L0.8NCo0.25 (left) and L0.8NCo0.5 (right) samples after the DRM tests at 700ºC under the 25:25 atmosphere.

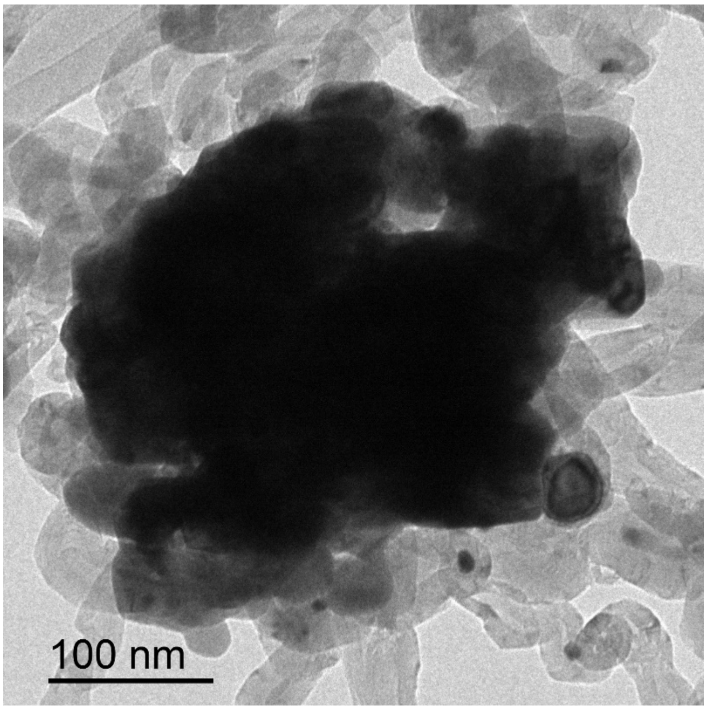

Figure S13. TEM image of the used L0.8NCo0.25 sample after the DRM test at 700ºC under the simulated biogas atmosphere.

ODRM catalytic tests under simulated biogas reactant atmosphere and using different operating regimes

The results presented in Figure S14 and Table S4 confirm that the effect of the operating regimes on ODRM is identical to that observed for DRM (under the 25:25 reactant atmosphere). Specifically: i) the relevance of the RWGS reaction increases in the high-GHSV configuration, as the CH_4 conversion remains almost unchanged while the CO_2 conversion notably increases; and ii) the amount of carbonaceous material deposited is also considerably higher.

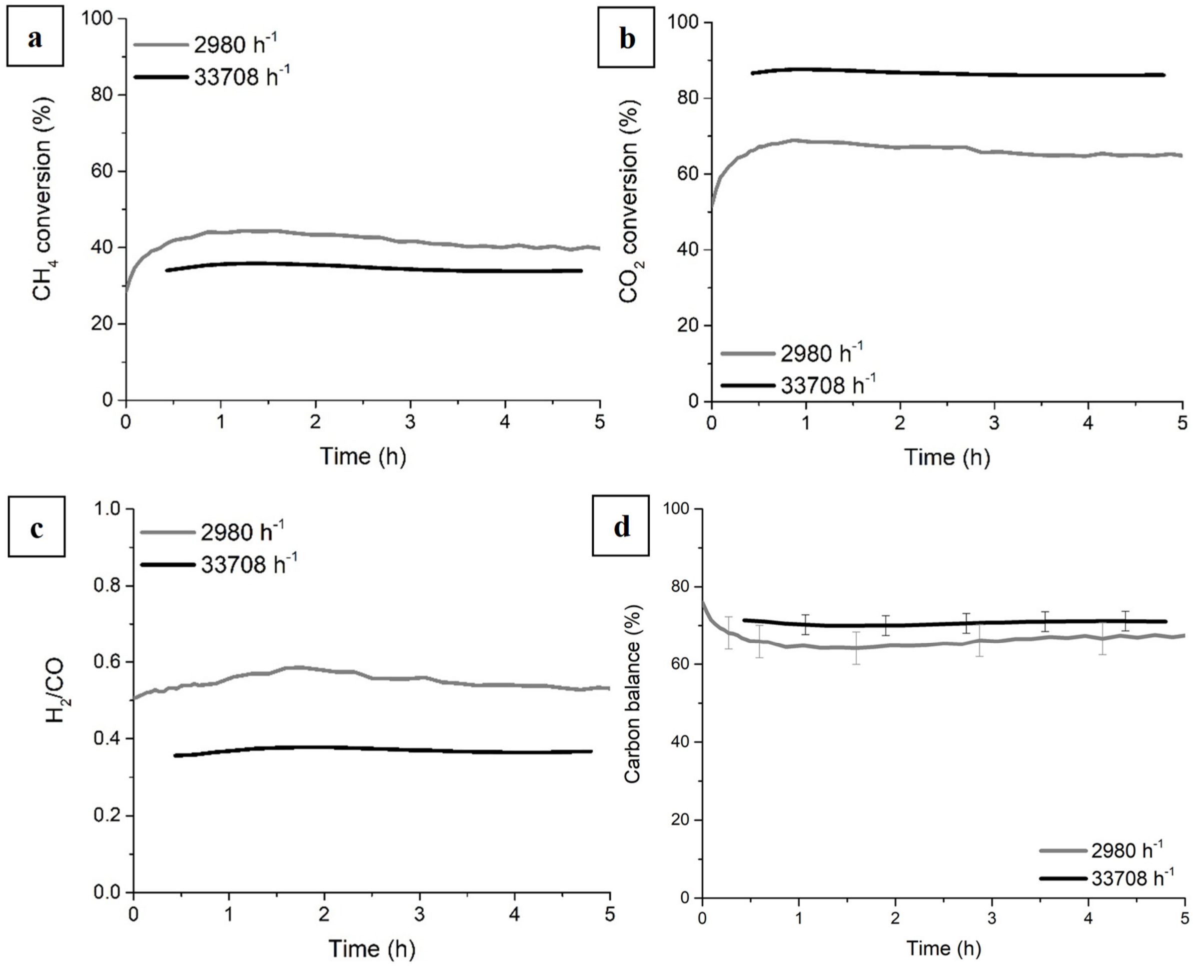

Figure S14. CH_4 (a) and CO_2 (b) conversions, H_2/CO ratio (c) and carbon balance (d) for the ODRM reaction at 700ºC under the simulated biogas reactant atmosphere, with 0.5 % O_2, and under both operating regimes.

Table S4. CH_4 and CO_2 conversions, CH_4 specific activity (a_{CH4}), H_2/CO product ratio, H_2 yield and amount of carbonaceous material deposited (C.D) during the ODRM reaction at 700ºC under the simulated biogas atmosphere, with 0.5 % O_2, and under both operating regimes.

GHSV (h^{-1})	X_{CH4} (5 h, %)	X_{CO2} (5 h, %)	ΔX_{CH4} / ΔX_{CO2} (%)[1]	a_{CH4} (5 h)[2]	Y_{H2} (5 h, %)	H_2/CO (5 h)	C.D (mg C g^{-1})
**2980**	40 ± 2	65 ± 1	-2 ± 4 / -2 ± 2	1721 ± 87	18 ± 1	0.53 ± 0.01	1195 ± 58
**33708**	34 ± 4	86 ± 4	0 / -1 ± 8	2918 ± 136	7 ± 1	0.37 ± 0.01	2117 ± 81

[1] Calculated as the difference between the reactant conversions at 5 h and at 30 min of reaction.

[2] As mmol of CH_4 converted per mol of Ni + Co and per minute.

TEM analysis of the L0.8NCo0.25 used sample after the ODRM + regeneration tests.

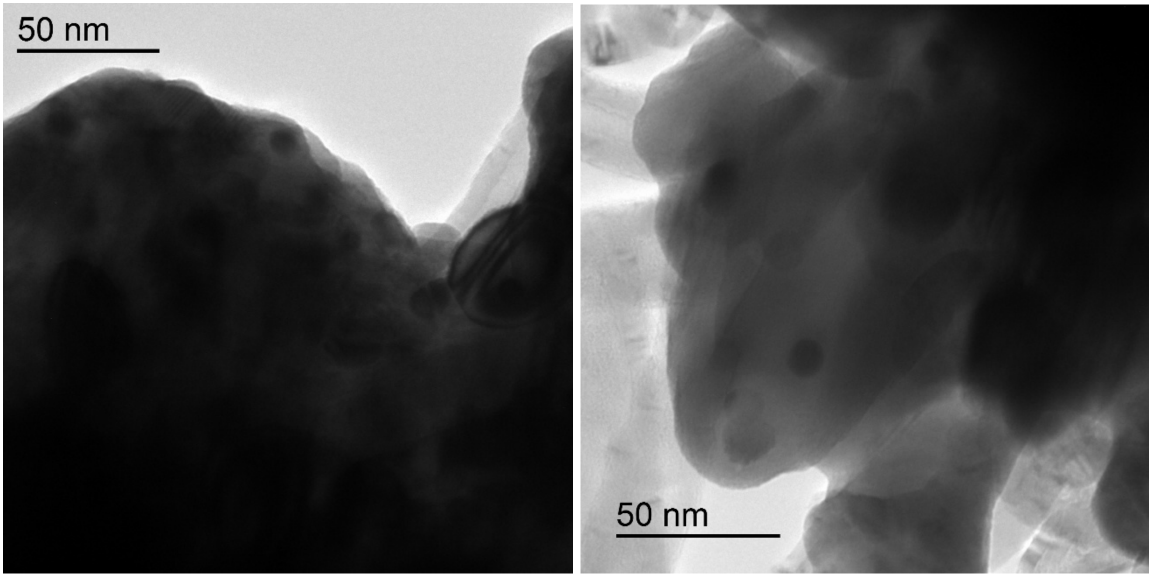

Figure S15. TEM images of the used L0.8NCo0.25 sample after the first (left) and second (right) ODRM cycle at 700ºC under the simulated biogas atmosphere.

Additional data

Table S5. Difference between the reactant conversions at 5 h and at 30 min (ΔX_{CH4} / ΔX_{CO2}) registered in the different catalytic tests for all the samples, and amount of carbonaceous material deposited calculated using the molar carbon balance.

Conditions (CH_4:CO_2:O_2, GHSV (h^{-1}))[1]	Sample	ΔX_{CH4} (%)	ΔX_{CO2} (%)	C.D (mg C g^{-1})
25:25:0, 2980	LN	12 ± 4	9 ± 2	373 ± 173
	L0.8N	9 ± 4	5 ± 2	1510 ± 532
	L0.8NCo0.1	12 ± 4	9 ± 2	387 ± 43
	L0.8NFe0.1	0	0	383 ± 37
	L0.8NMn0.1	12 ± 4	8 ± 2	347 ± 32
	L0.8NCo0.25	8 ± 4	7 ± 2	25 ± 7
	L0.8NCo0.5	5 ± 4	4 ± 2	20 ± 7
65:35:0, 2980	L0.8N	-13 ± 4	-8 ± 2	3739 ± 348
	L0.8NCo0.25	-5 ± 4	-	4230 ± 310
25:25:0, 33708	L0.8NCo0.25	21 ± 8	13 ± 8	3668 ± 216
25:25:0.5, 33708	L0.8NCo0.25	16 ± 8	12 ± 8	879 ± 138
25:25:0.9, 33708		19 ± 8	17 ± 8	1392 ± 133
65:35:0.5, 33708		0	-	2864 ± 166
65:35:0.9, 33708		4 ± 8	-	1107 ± 175
65:35:0.5, 33708 Cycle 1	L0.8NCo0.25	-	-	992 ± 234
65:35:0.5, 33708 Cycle 2		-5 ± 4	-5 ± 2	545 ± 77

[1] The missing values indicated with "-" present a high error and have been discarded from this data.

Probing the assumption of pure DRM for the calculation of the reaction extent

As described in the Materials and Methods section of the manuscript, the outlet molar flowrates were corrected for volumetric expansion using the standard extent of reaction approach (Equation E11), where v_i is the stoichiometric coefficient of species i (negative for reactants and positive for products), and ξ is the extent of reaction, which was calculated by Equation E12 ($\bar{F}_{lr,in}$ is the inlet molar flowrate of the limiting reactant, $\chi_{lr,out}$ is its outlet molar fraction, and δ is the net change in the total number of gaseous moles per mole of limiting reactant consumed (for pure DRM reaction, the total number of moles changes from 2 (inlet) to 4 (outlet), resulting in a net increase of 2 moles per mole of limiting reactant; therefore, δ is equal to 2).

However, the H_2/CO ratios experimentally determined evidence that RWGS reaction plays a significant role during the catalytic tests, so, this assumption could be considered an oversimplification. Thus, the assumption applied in the manuscript should be probed. The key fact is that the RWGS reaction is equimolar: it consumes 2 moles of gas and produces 2 moles of gas. Thus, it does not change the total number of gaseous moles and, consequently, the net change in the total gaseous molar flowrate (including water vapor) is governed by the DRM reaction. Thus, for the total wet gaseous stream, the net change in moles per mole of limiting reactant converted is still 2.

However, since the GC used analyzes the outlet stream after water condensation, the mole fractions determined correspond to the dry gas composition. Consequently, to properly use the dry mole fractions in the carbon balance, the total wet flowrate must be corrected by subtracting the water generated. So, assuming an average H_2/CO value of 0.5 (based in the experimental data presented in the manuscript), we can directly calculate the extents of DRM and RWGS and, after, the outlet molar flowrates of H_2 and CO are:

$$\bar{F}_{H_2,out} = 2\,\xi_{DRM} - \xi_{RWGS}$$

$$\bar{F}_{CO,out} = 2\,\xi_{DRM} + \xi_{RWGS}$$

So:

$$H_2/CO = \frac{\bar{F}_{H_2,out}}{\bar{F}_{CO,out}} = \frac{2\,\xi_{DRM} - \xi_{RWGS}}{2\,\xi_{DRM} + \xi_{RWGS}} = 0.5;$$

$$;\ 2\,\xi_{DRM} - \xi_{RWGS} = 0.5\,(2\,\xi_{DRM} + \xi_{RWGS}) = \xi_{DRM} + 0.5\,\xi_{RWGS};$$

$$; \xi_{DRM} = 1.5\ \xi_{RWGS}$$

Thus, the molar flowrate of water produced is:

$$\bar{F}_{H_2O,out} = \xi_{RWGS} = 0.67\ \xi_{DRM}$$

and the total dry outlet molar flowrate is:

$$\bar{F}_{total,dry} = \bar{F}_{total,wet} - \bar{F}_{H_2O,out}$$

Stablishing that $\bar{F}_{total,wet} = \bar{F}_{total,in} + \delta\ \xi_{DRM}$, with $\delta = 2$:

$$; \bar{F}_{total,dry} = \left(\bar{F}_{total,in} + 2\ \xi_{DRM}\right) - 0.67\ \xi_{DRM} = \bar{F}_{total,in} + 1.33\ \xi_{DRM}$$

The presented procedure shows that, if a dry basis is assumed, the resulting δ value would be 1.33. However, note that this is merely a mathematical transformation, and our original approach (δ = 2) is consistent with the observed H_2/CO ratio and does not require changing the corresponding equation. In this line, to show that no significant differences are found between the carbon balance plots calculated using the two δ values (2 and 1.33), in the Figure S16 the data for three selected catalytic are compared:

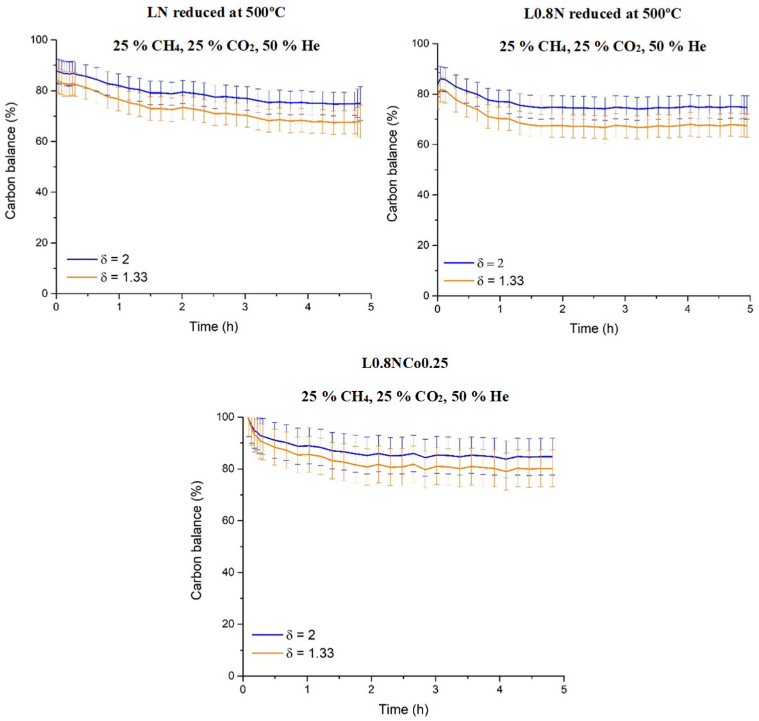

Figure S16. Carbon balance profiles for three selected catalysts calculated using the two δ values (2 and 1.33).

It can observe that the carbon balance profiles evaluated with both methodologies remained almost consistent, considered the error bars, thus confirming that the originally formulated equation (using $\delta = 2$) seems being adequate as does not introduce significant errors.

In a next approach, we could consider the inclusion of the methane decomposition reaction (thus using ξ_{MD} as the corresponding reaction extent), which is the source of the carbonaceous deposits. However, it would not be possible due to a mathematical limitation found. Thus, considering that the outlet molar flowrates (wet basis) are:

$$\bar{F}_{CH_4,out} = \bar{F}_{CH_4,in} - \xi_{DRM} - \xi_{MD}$$

$$\bar{F}_{CO_2,out} = \bar{F}_{CO_2,in} - \xi_{DRM} - \xi_{RWGS}$$

$$\bar{F}_{H_2,out} = 2\,\xi_{DRM} - \xi_{RWGS} + 2\,\xi_{MD}$$

$$\bar{F}_{CO,out} = 2\,\xi_{DRM} + \xi_{RWGS}$$

$$\bar{F}_{H_2O,out} = \xi_{RWGS}$$

Using the GC data, we determine three independent molar fractions, that is, χ_{CH4}, χ_{CO2} and χ_{CO}. Despite having detected four gaseous components, the χ_{H2} is not independent since:

$$\chi_{H_2} = 1 - \chi_{CH_4} - \chi_{CO_2} - \chi_{CO}$$

If we consider the total dry outlet molar flowrate, together with the contributions of water vapor and the carbonaceous deposited, the atomic balances are:

$$\mathbf{C}\text{: } \bar{F}_{total,dry}\left(\chi_{CH_4} + \chi_{CO_2} + \chi_{CO}\right) + \dot{n}_C = 2$$

$$\mathbf{O}\text{: } \bar{F}_{total,dry}\left(2\,\chi_{CO_2} + \chi_{CO}\right) + \bar{F}_{H_2O,out} = 2$$

$$\mathbf{H}\text{: } \bar{F}_{total,dry}\left(4\,\chi_{CH_4} + 2\,\chi_{H_2}\right) + 2\,\bar{F}_{H_2O,out} = 4$$

After, we examine the linear dependence of these equations and dividing the hydrogen atomic balance by 2:

$$\bar{F}_{total,dry}\left(2\,\chi_{CH_4} + \chi_{H_2}\right) + \bar{F}_{H_2O,out} = 2$$

And considering:

$$2\,\chi_{CH_4} + \chi_{H_2} = 2\,\chi_{CO_2} + \chi_{CO}$$

The combination of both expressions results in:

$$\bar{F}_{total,dry}\left(2\,\chi_{CO_2}+\chi_{CO}\right)+\bar{F}_{H_2O,out}=2$$

Which is the oxygen atomic balance. Therefore, hydrogen atomic balance is a linear combination of the latter. Consequently, the system is reduced to two independent equations with three unknown variables, resulting in an underdetermined system. Note that, the same conclusion would be expected for the Boudouard reaction.

Thus, these mathematical demonstrations justify the methodology used for carbon balance.
